# Microliter-scale reaction arrays for economical high-throughput experimentation in radiochemistry

**DOI:** 10.1038/s41598-022-14022-2

**Published:** 2022-06-17

**Authors:** Alejandra Rios, Travis S. Holloway, Philip H. Chao, Christian De Caro, Chelsea C. Okoro, R. Michael van Dam

**Affiliations:** 1 Physics and Biology in Medicine Interdepartmental Graduate Program, University of California Los Angeles (UCLA), Los Angeles, CA USA; 2 Department of Molecular & Medical Pharmacology, David Geffen School of Medicine, UCLA, Los Angeles, CA USA; 3 Department of Bioengineering, UCLA, Los Angeles, CA USA; 4 Department of Physics & Astronomy, UCLA, Los Angeles, CA USA; 5 Institute for Society and Genetics, UCLA, Los Angeles, CA USA; 6 Crump Institute for Molecular Imaging, UCLA, Los Angeles, CA USA

**Keywords:** High-throughput screening, Imaging, Lab-on-a-chip, Biomedical engineering, Nuclear chemistry, Chemical synthesis, Diagnostic markers

## Abstract

The increasing number of positron-emission tomography (PET) tracers being developed to aid drug development and create new diagnostics has led to an increased need for radiosynthesis development and optimization. Current radiosynthesis instruments are designed to produce large-scale clinical batches and are often limited to performing a single synthesis before they must be decontaminated by waiting for radionuclide decay, followed by thorough cleaning or disposal of synthesizer components. Though with some radiosynthesizers it is possible to perform a few sequential radiosyntheses in a day, none allow for parallel radiosyntheses. Throughput of one or a few experiments per day is not well suited for rapid optimization experiments. To combat these limitations, we leverage the advantages of droplet-radiochemistry to create a new platform for high-throughput experimentation in radiochemistry. This system contains an array of 4 heaters, each used to heat a set of 16 reactions on a small chip, enabling 64 parallel reactions for the rapid optimization of conditions in any stage of a multi-step radiosynthesis process. As examples, we study the syntheses of several ^18^F-labeled radiopharmaceuticals ([^18^F]Flumazenil, [^18^F]PBR06, [^18^F]Fallypride, and [^18^F]FEPPA), performing > 800 experiments to explore the influence of parameters including base type, base amount, precursor amount, solvent, reaction temperature, and reaction time. The experiments were carried out within only 15 experiment days, and the small volume (~ 10 μL compared to the ~ 1 mL scale of conventional instruments) consumed ~ 100 × less precursor per datapoint. This new method paves the way for more comprehensive optimization studies in radiochemistry and substantially shortening PET tracer development timelines.

## Introduction

The field of molecular imaging has created positron emission tomography (PET) and single-photon emission computed tomography (SPECT)^1^for the visualization and quantification of biochemical processes in living subjects. The use of biologically active molecules tagged with short-lived radionuclides enables such imaging to be performed non-invasively at the whole-body level. PET is used in a wide range of research in small animals and humans to help understand disease pathways^2,3^, measure pharmacokinetics, confirm the biological effects of new therapeutic compounds^4,5^, monitor disease progression, or monitor the response to treatment^6–8^. Common radioisotopes used in PET include C-11 (t_1/2_ = 20.4 min), F-18 (t_1/2_ = 109.8 min), and Ga-68 (t_1/2_ = 67.7 min), among others.

Radiopharmaceuticals are typically prepared using automated synthesizers to limit radiation exposure to personnel and improve reproducibility^9^. Preparing a batch of a radiopharmaceutical is expensive due to the high cost of the radiosynthesizer, radiation shielding, reagents, radioisotope, and skilled personnel. These costs are greatly multiplied for optimization studies, which require many syntheses to be performed under different conditions. Furthermore, most radiosynthesizers are designed for only one or a few consecutive radiosyntheses per day, thus optimization studies can take weeks or months which further increase labor cost, resource usage, and radioisotope cost.

Recently, multiple approaches have been described to significantly improve the throughput of radiochemistry experiments. Zhang et al*.* performed reactions without radioactivity but mimicked the ultra-low concentrations associated with radionuclides, and leveraged the high sensitivity of LC–MS/MS analysis to assess product yield^10^. While avoiding the use of radioactivity increased the throughput of experiments, the reliance on conventional reaction volumes still consumed significant time and reagents to obtain each data point. As an alternative, microfluidic platforms and miniaturized radiochemistry techniques provide promising avenues to increase throughput while minimizing reagent consumption^11–14^ by borrowing concepts from high-throughput experimentation in organic chemistry^15–17^. Several groups have shown that dozens of small-scale radiochemical reactions (i.e. 10s of µL each, compared to the ~ 0.5–2.0 mL used in conventional setups) can be sequentially performed using flow-chemistry capillary reactor platforms with crude products collected and analyzed offline^18–21^. While parameters like temperature and reagent flow rates can be readily studied in a high-throughput manner, others, such as reaction solvent or the conditions for the drying/activation of the [^18^F]fluoride, cannot. Another optimization platform used a polydimethylsiloxane (PDMS) microfluidic chip to prepare ultra-small batches (~ 100 nL each) for screening of aqueous protein radiolabeling conditions but was limited to varying reagent ratios and pH^22,23^.

Small-volume vial-based reactions have also been used for optimization^24^ and enable a wider range of parameters to be studied. Recently, Laube et al*.* reported the use of multi-vial heating blocks to perform up to ~ 50 radiofluorinations per day, each involving drying a small aliquot of [^18^F]fluoride eluted from a QMA cartridge, followed by reaction at the 25–50 µL scale^25^. While demonstrating parallelism and low reagent consumption, this technique required significant manual handling of vials, including installation and removal of vial caps. In addition, it is well known that the detailed heating characteristics of the system are essential to consider^26^, and after optimization in small vials the conditions may have to be adapted to a conventional synthesizer for routine automated production.

Our group recently pioneered a microfluidic platform, in which reactions are performed at an even smaller scale (i.e. 1–10 µL) in droplets confined in surface-tension traps patterned on a surface^27^. Under these conditions, droplet reactions typically have yields comparable to conventional methods but allow shorter synthesis time and up to ~ 100 × lower reagent consumption per reaction^28–30^. Of particular note, after optimization under low activity conditions, larger scale production (e.g. one or a few clinical doses) can be achieved under identical conditions using an automated droplet-based radiosynthesizer^31,32^ by a simple increase of starting activity. To increase throughput, we created chips with multiple reaction sites for performing up to 16 droplet-based syntheses in parallel, all with the same reaction temperature and time but with varying volumes or concentrations of reagents^33^. A preliminary study showed the possibility of optimizing several parameters in the synthesis of [^18^F]Fallypride, including the amount of base, precursor concentration, and droplet reaction volume. In this paper, throughput and flexibility are further increased by introducing an array of 4 independent heaters, enabling operation of 4 chips in parallel. This improved platform allows the parallel exploration of additional reaction variables (reaction temperature and time) that cannot be conveniently studied with a single chip at a time.

## Results

### Platform design

**Figure 1:**
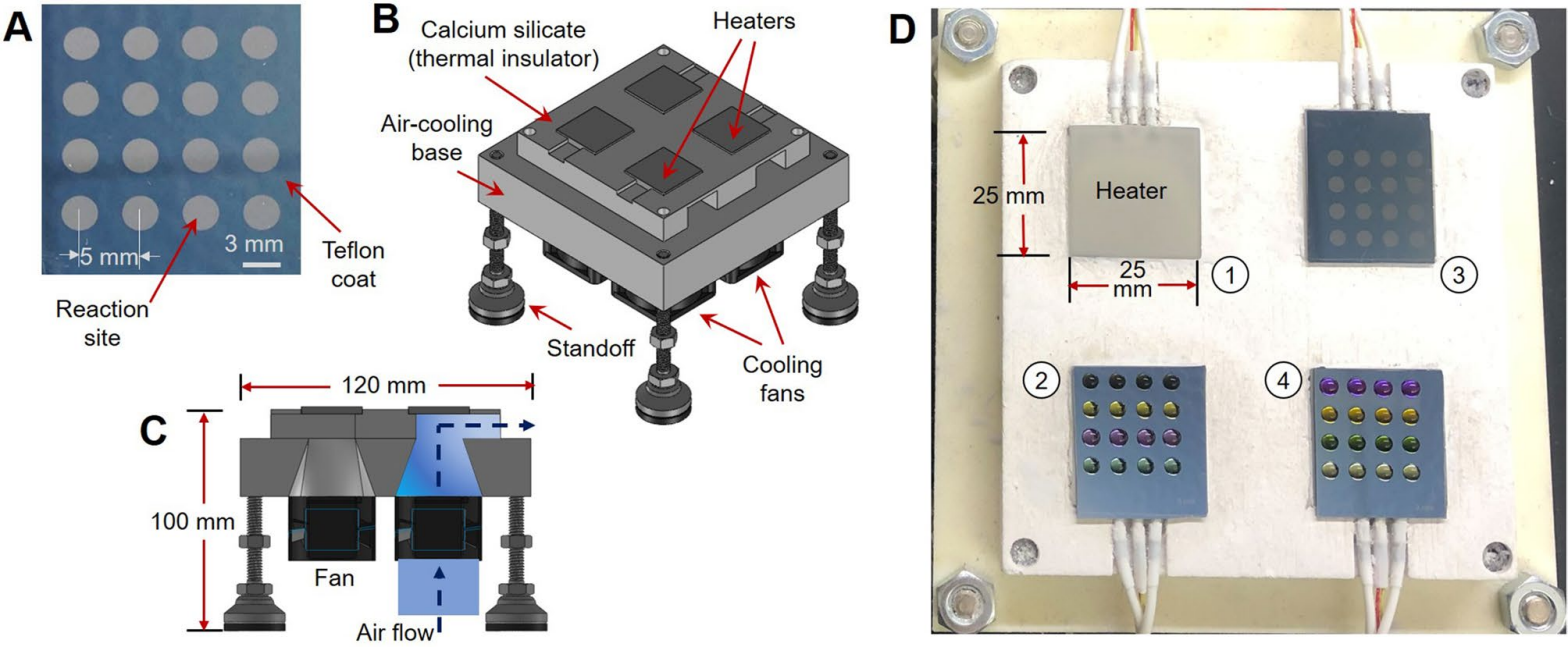
High-throughput reaction apparatus. (**A**) Photograph of multi-reaction chip. (**B**) CAD model showing platform components. (**C**) Cross-section view of the multi-heater platform showing the path of forced-air cooling. (**D**) Photograph of the platform (from above) with multi-reaction chips installed.

Arrays of reactions were performed in droplet format on 25.0 × 27.5 mm^2^ Teflon-coated silicon “chips” (Fig. 1A). Each reaction was confined to a 3 mm diameter circular hydrophilic site (made by etching away the Teflon coating) that acts as a surface tension trap. Details of the chip fabrication have been previously reported^33^.

Multiple chips were operated in parallel using a custom-built four-heater platform (Fig. 1B-D). To provide radiation protection, the platform was operated inside a hot cell, but the control system could be placed outside to minimize the footprint. The platform comprised four 25 x 25 mm^2^ ceramic heaters glued with epoxy on top of a thermally insulating frame CNC-machined from a calcium silicate composite, which was in turn affixed atop of a 3D-printed nylon piece to direct cooling air to each heater from a set of four 36 mm 12 V DC fans (Supplementary Sect. 1). Thermal simulations were performed to determine an appropriate heater spacing to avoid thermal crosstalk from neighboring heaters (Supplementary Sect. 2). The chips were affixed to the heaters with a thin layer of thermal paste.

Each heater (and fan) was independently controlled, enabling the set of reactions on each multi-reaction chip to be performed at a unique temperature or duration (Supplementary Sect. 1). For each heater, the signal from the integrated K-type thermocouple was amplified and connected to an analog input of a data acquisition module (DAQ). Heaters were powered with 120VAC switched by a solid-state relay driven by a digital output of the DAQ using an on–off controller implemented in LabView (National Instruments). After the desired heating time, forced-air cooling was activated using a digital output of the DAQ to power the corresponding fan via a Darlington driver circuit.

After calibrating the heaters (Supplementary Sect. 3), temperature stability was assessed by monitoring the integrated thermocouple with respect to time (Supplementary Figure S6). At each setpoint tested, heating took only ~ 5 s, and temperature exhibited < 1 °C fluctuation once stabilized (Supplementary Table S1). Forced-air cooling to 30 °C took ~ 3 min from 140 °C, ~ 2.5 min from 100 °C, and ~ 1.2 min from 50 °C. In addition, spatial temperature distribution of each heater was visualized via thermal imaging. All heaters exhibited uniform surface temperature (Supplementary Figures S7, S8, and Table S2), except near the edges (where deviation > 2% from the mean was observed). In all cases, the extent of this unusable region was limited to < 1.5 mm on each edge of the heater. Thus, the multi-reaction chips were designed with a 2.4 mm unused boundary, ensuring that all 16 reaction sites were entirely located within the uniform portion of the heater surface (Supplementary Figure S9)^33^. A previous study confirmed the consistency of reactions at different sites on the chip and the negligible degree of cross-contamination from one site to another^33^.

With the platform, up to 64 radiochemical syntheses could be performed in parallel, each reaction using ~ 100 × less reagents than conventional approaches. Because all steps, including [^18^F]fluoride drying, are performed on-chip, the conditions used in any part of the synthesis can be explored in a high-throughput fashion.

### Synthesis optimization

We used this new platform to perform extensive studies of the syntheses of several clinically relevant PET tracers: [^18^F]Flumazenil [^18^F]FMZ), [^18^F]PBR06, [^18^F]Fallypride, and [^18^F]FEPPA.

For each radiopharmaceutical, an extensive set of experiments was performed to compare the influence of different reaction conditions related to [^18^F]fluoride drying and the radiofluorination reaction. Our goals were to better understand the influence of various reaction parameters and to develop efficient microscale synthesis protocols for these tracers. Initial droplet reaction conditions were determined essentially by reducing volumes ~ 100 × from conventional macroscale protocols. In general, experiments were performed in batches of 64 simultaneous reactions (4 chips × 16 reactions each), exploring 16 different conditions, each with n = 4 replicates.

**Figure 2:**
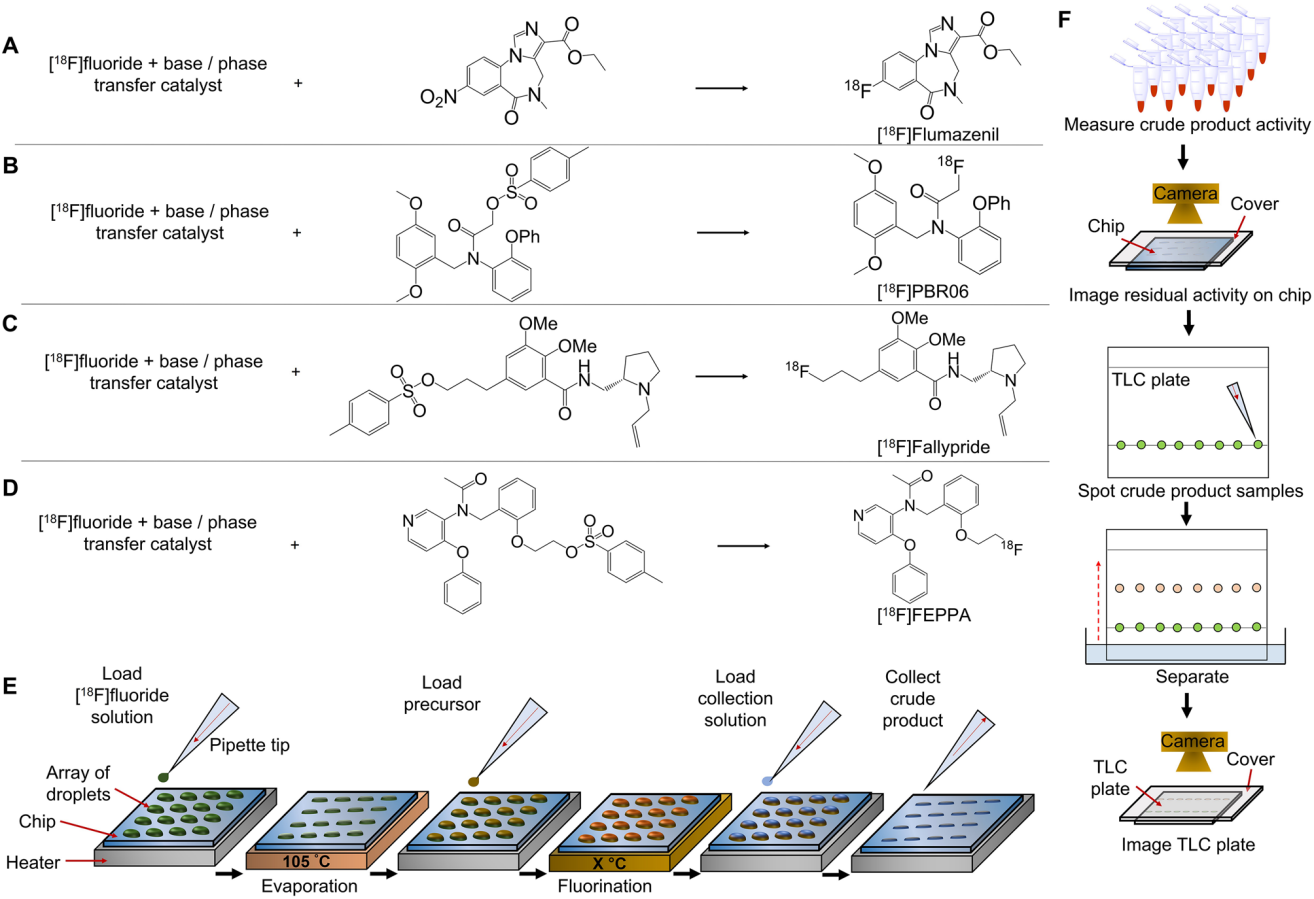
Optimization process. (**A**) Scheme for the radiosynthesis of [^18^F]Flumazenil. (**B**) Synthesis of [^18^F]PBR06. (**C**) Synthesis of [^18^F]Fallypride. (**D**) Synthesis of [^18^F]FEPPA. (**E**) Experimental procedure for performing parallel radiosyntheses using a (4 × 4) multi-reaction microdroplet chip. Concentrations, solvents, and volumes can be varied from site to site, and temperature and heating time can be varied from chip to chip. (**F**) Procedure for reaction performance analysis. Activities of collected crude samples are measured using a dose calibrator and compared with starting activity to determine collection efficiency. Residual activity on chip is analyzed via Cerenkov imaging. Crude samples are analyzed via radio-TLC to determine the fluorination efficiency.

Figure 2 illustrates the synthesis scheme for each tracer and the generalized process for one set of 16 reactions. At each site, an 8 μL droplet of [^18^F]fluoride stock solution ([^18^F]fluoride mixed with the desired amount and type of base and phase-transfer catalyst) is added to the reaction site and dried. (Though drying parameters to eliminate residual water could also be studied, drying was performed for 1 min at 105 °C in all experiments.) Next, 8 µL of precursor solution (6 µL for [^18^F]Fallypride) with the desired concentration and reaction solvent is added to the dried residue and reacted at elevated temperature for the desired time. Reaction volume could also be studied as a parameter but was not explored here. After the reaction is complete, crude product is collected. Though collecting parameters could be optimized to minimize residual activity on the chip, we performed product collection in all cases by dispensing 10 μL of collection solution to the reaction site and aspirating the volume and repeating these steps 4 × for a total of 40 μL of collected crude product. The reaction performance was determined by measuring both the conversion of [^18^F]fluoride to product via radio-TLC, as well as the recovered activity from each reaction (compared to starting activity, i.e. collection efficiency) to determine an overall crude radiochemical yield (RCY). TLC analysis was performed using recently reported multi-lane methods with 8 samples per plate^34^.

### Optimization of [^18^F]Flumazenil radiosynthesis

**Figure 3:**
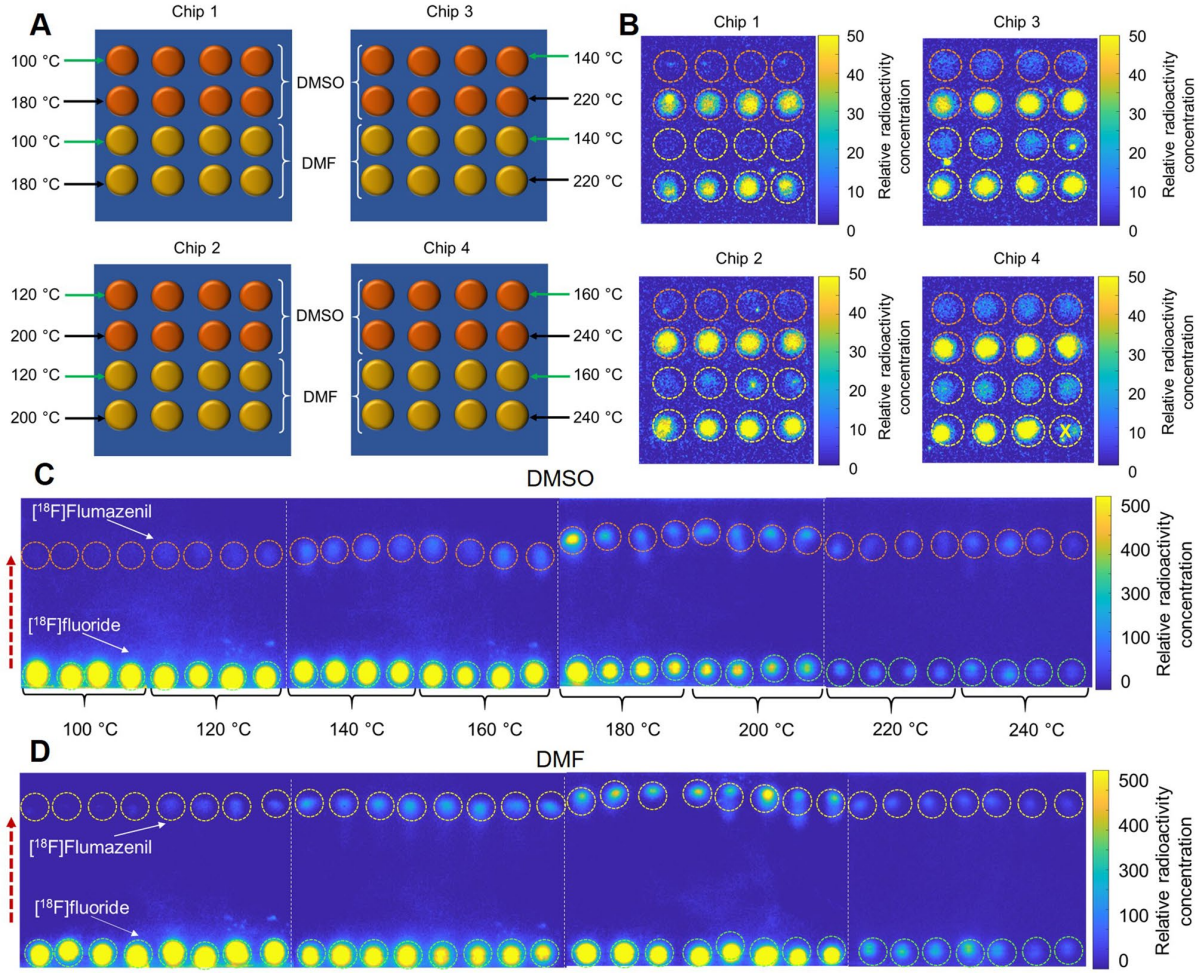
Experimental set up for one batch of experiments that explored the influence of reaction temperature (8 values) and solvent (2 types) for the synthesis of [^18^F]Flumazenil. (**A**) Assignment of 64 reaction sites. Half of the reaction sites were used first to explore 4 different temperatures in the first set of 32 simultaneous reactions. Then the other half of the sites were used for the remaining 4 temperatures. (**B**) Cerenkov images show the distribution of the residual activity on each chip after collecting the crude products. Radioactivity signal is decay-corrected to a common timepoint for all images. The reaction marked with an “X” was not analyzed (by mistake the precursor droplet was not added to reaction site). (**C**) Cerenkov images of developed TLC plates (each containing 8 samples) for reactions that used DMSO as the reaction solvent. (**D**) Separated crude samples using DMF as the reaction solvent. Dashed circles indicate the ROIs used for analysis. The dashed red arrow indicates the direction of solvent movement during development. White dotted lines represent the boundary of each multi-sample plate.

**Figure 4:**
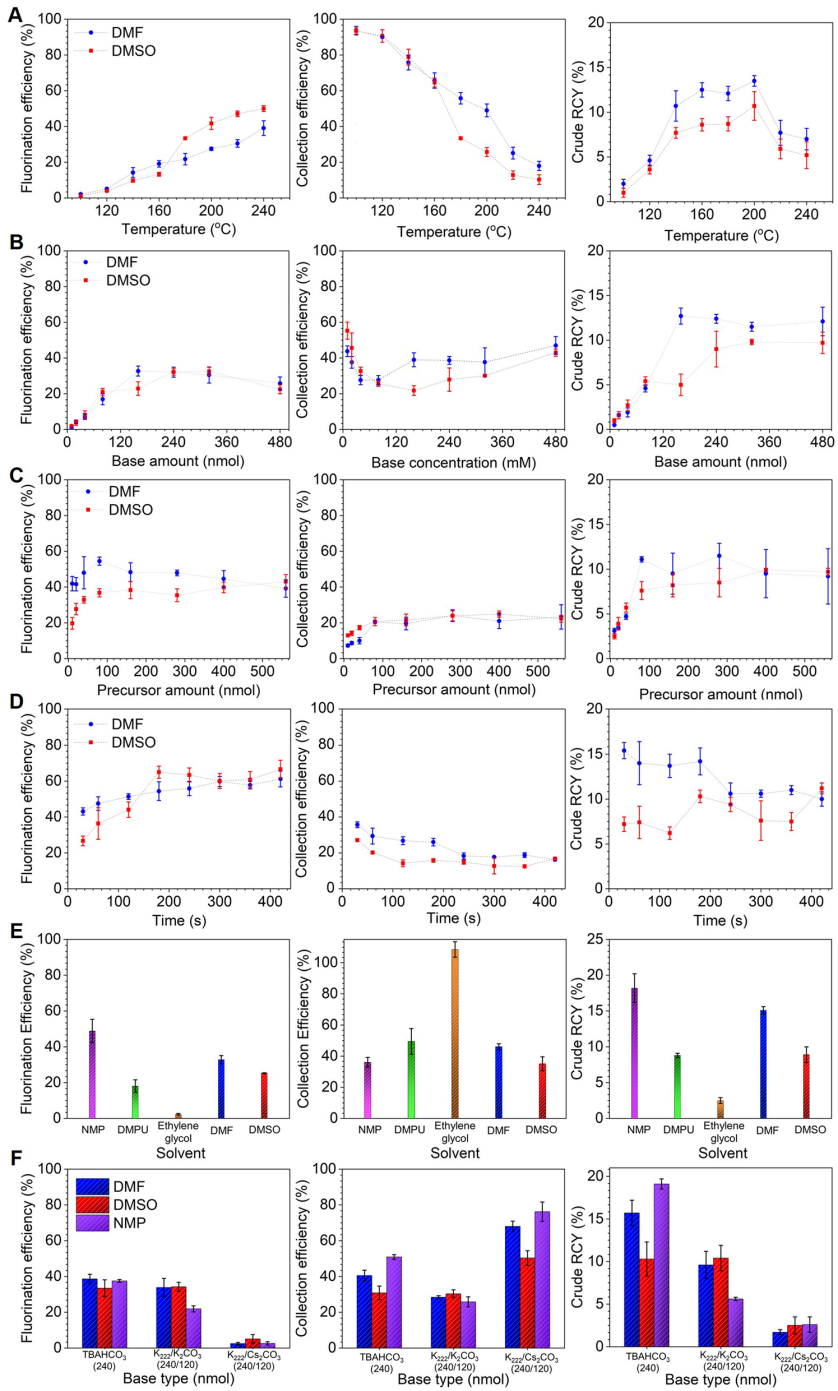
Influence of reaction parameters on the performance of the microdroplet radiosynthesis of [^18^F]Flumazenil. For each parameter, influence on fluorination efficiency, collection efficiency, and crude RCY are plotted individually. (**A**) Effect of temperature (and solvent). Precursor amount: 280 nmol. Reaction volume: 8 µL. Base amount: 480 nmol. Reaction time: 2 min. (**B**) Effect of amount of base (and solvent). Precursor amount: 280 nmol. Reaction volume: 8 µL. Reaction temperature: 200 °C. Reaction time: 2 min. (**C**) Effect of precursor concentration (and solvent). Reaction volume: 8 µL. Base amount: 240 nmol. Reaction time: 2 min. Reaction temperature 200 °C. (**D**) Effect of reaction time (and solvent). Precursor amount: 280 nmol. Reaction volume: 8 µL. Base amount: 240 nmol. Reaction temperature: 200 °C. (**E**) Effect of reaction solvent. Precursor amount: 280 nmol. Reaction volume: 8 µL. Base amount: 240 nmol. Reaction temperature: 200 °C. Reaction time: 0.5 min. (**F**) Effect of the base type (and solvent). Precursor amount: 280 nmol. Reaction volume: 8 µL. Base amount: 240 nmol. Reaction temperature: 200 °C. Reaction time: 0.5 min.

[^18^F]Flumazenil is used to quantify changes in the density of GABA_A_ receptors associated with Alzheimer’s disease, Schizophrenia, neuronal plasticity, and sensory processes^35^. We focused on the route from the commercially-available nitromazenil precursor^18,36–40^, for which reported isolated yields are in the range of 8 to 30%^36–40^. Though other synthesis routes have led to higher yields, they were not pursued here due to the lack of commercial availability of the diaryliodonium tosylate precursor^41^ or the very low molar activity (0.37 GBq/µmol [0.01 Ci/µmol]) of the isotopic exchange method^42^. Previous optimization studies using macroscale and flow chemistry approaches (Table 1) have typically compared just a couple of values for parameters studied and often with few if any replicates^18,36–40^. Leveraging the increased throughput of our platform, we performed a series of experiments to explore more comprehensive ranges of each parameter with finer granularity (typically 8 values each) and more replicates. Parameters explored included: (i) reaction temperature, (ii) amount of base, (iii) amount of precursor, (iv) reaction time, (v) reaction solvent, and (vi) type of base and phase-transfer catalyst. Full details and results for each set of experiments can be found in Supplementary Sect. 2. Since most literature reported the use of the solvents N,N-dimethylformamide (DMF) and dimethyl sulfoxide (DMSO)^18,38^, the studies we performed for parameters i – iv were carried out using each of these solvents. As an example of how each experiment was set up, Fig. 3 shows how four chips were used to explore reaction temperature. The figure also shows the images of residual radioactivity on the chips after synthesis, and the Cerenkov images of the TLC plates used to evaluate conversion. The resulting performance calculations for each condition are tabulated in Supplementary Table S3 and the performance is plotted in Fig. 4A. The fluorination efficiency increased strongly with temperature. However, unlike other droplet-based reactions, volatile losses during the fluorination reaction, as well as residual activity stuck to the chip after collection, led to declining collection efficiency with increasing temperature. (Generally, the amount of volatile loss dominated and was about 15—10 × higher than the residual loss.) The resulting crude RCY exhibited a peaking behavior with a maximum of 13.5 ± 0.6 (n = 4) at 200 °C (with DMF). Consistent with these trends, Wong et al*.* found temperature of a flow reactor to be an essential factor with fluorination efficiency increasing from ~ 0% at 120 °C to ~ 20% at 160 °C using DMF as the solvent, and from ~ 0% to ~ 5% using DMSO^18^. Mandap et al*.*, using a microwave reactor, also found that fluorination efficiency increased substantially with temperature to a maximum value and then declining somewhat at higher temperatures^38^.

**Table 1:** Summary of parameters and conditions tested in reports of optimization of [^18^F]Flumazenil using nitromazenil as precursor in microscale and macroscale platforms.

	This work	Wong et al^18^ (2012)	Nasirzadeh et al^37^. (2016)	Mandap et al^38^. (2009)	Massaweh et al^39^. (2009)	Ryzhikov et al^40^. (2005)
Synthesizer type	Microscale (droplet format)	Microscale (flow format)	Macroscale	Macroscale (microwave)	Macroscale	Macroscale
Solvents	DMSO, DMF, NMP, DMPU, ethylene glycol	DMSO, DMF, MeCN	DMF	DMSO, DMF, MeCN	DMF	DMSO, DMF
Reaction times (min)	0.5, 1, 2, 3, 4, 5, 6, 7	2.5	15, 30	2, 5, 10	30	15, 30
Temperatures (°C)	100, 120, 140, 160, 180, 200, 220, 240	110, 120, 130, 140, 160	150	90, 140, 160, 180, 200*	160	130, 160
Base types	TBAHCO_3_, K_222_/K_2_CO_3_, K_222_/Cs_2_CO_3_	K_222_/KHCO_3_	K_222_/K_2_CO_3_	K_222_/K_2_CO_3_	K_222_/K_2_CO_3_	K_222_/K_2_CO_3_
Base amounts (nmol)	480, 320, 240, 160, 80, 40, 20, 10 and 240/120	2850/2590	25,000/12,000	12,000/6000^#^	2800/ 1200	25,000/12,000^┼^
Precursor amounts (nmol)	560, 400, 280, 160, 80, 40, 20, 10	1500	24,220, 12,000, 5100, 3030	24,000, 15,000, 12,000, 51,000, 3000	18,000, 21,000	6000, 12,000, 13,000, 13,000, 19,000, 24,000, 25,000, 25,000, 25,400, 36,000
Total number of different conditions tested	85	13	3	19	1	14
Total number of experiments performed	335	13	23	52	15	14

^#^Not reported but the amount of K_222_ was computed based on the amount of precursor and an indicated precursor to K_222_/K_2_CO_3_ molar ratio of 0.5:1. Ratio of K_222_ to K_2_CO_3_ needed to calculate K_2_CO_3_ was inferred from a paper they referenced^72^.

^┼^Based on 1:1 precursor to K_222_/K_2_CO_3_ molar ratio. Note: different volumes of solvent were used as an additional parameter (0.5, 1.0, 1.5, and 2.0 mL).

*In the microwave reactor, the pressure was also varied (0, 100, and 200 kPa).

We should point out that typically only the radiofluorination efficiency (as determined by radio-HPLC or radio-TLC) and/or radiochemical yield is reported in optimization experiments, making it difficult to make detailed comparisons with droplet reactions. Reporting only radiofluorination efficiency can be misleading as many potential losses (e.g. volatile losses or residual activity stuck on vials or tubing, which can be significant^43^) are not accounted for. Reporting only radiochemical yield accounts for losses, but all the losses (from various synthesis steps or purification) are lumped together. Significant discrepancies have been reported between radiochemical conversional and radiochemical yield for [^18^F]Flumazenil^36,37,39^. For example, Vaulina et al*.* observed fluorination efficiency (TLC) of 25% but obtained only a 2% isolated yield after HPLC purification and SPE formulation, or 9% after SPE-based purification/formulation^36^. Massaweh et al*.* found that despite a fluorination efficiency (TLC) of 27–35%, isolated yield was only 2–5% ^40^, though it improved to 15–20% after mobile phase optimization^39^. While these discrepancies may reflect high losses during the purification/formulation step^36^, these reports do not contain sufficient details or data to rule out other losses (e.g. residual activity on reaction vessel or tubing, volatile losses, etc.)

The temperature was fixed at 200 °C for subsequent experiments. With increasing base amount (Fig. 4B), we observed the fluorination efficiency to increase from near zero and plateau at a maximum value when base amount reached ~ 150–200 nmol. Collection efficiency exhibited an inverse behavior, and the overall crude RCY for DMF (the higher performing solvent) exhibited a sharp increase and then plateau starting at ~ 160 nmol of base. 240 nmol (where the crude RCY was only marginally lower) was selected as the optimal amount to provide robustness against pipetting errors. Study of increasing precursor amount (Fig. 4C) showed rapid increases up to ~ 80 nmol and then a plateau, for fluorination efficiency, collection efficiency and crude RCY. The highest crude RCY (with DMF, the higher performing solvent) occurred at a precursor amount of 280 nmol, which was selected as the optimal condition. The strong impact of precursor amount below the plateau is consistent with Mandap et al*.*, who reported low fluorination efficiency (< 3%, n = 1) for 1 mg of precursor in DMF at 160 °C, and high values (~ 30%) with 2–8 mg of precursor. Ryzhikov et al*.* also found marked differences in fluorination efficiency in pairwise comparisons of precursor amounts^40^. Unfortunately, the reaction volume is given as a range in both papers, making it impossible to compare the concentration values. In many reactions, the ratio of base to precursor is a relevant parameter and we thus plotted the reaction performance as a function of this ratio in Supplementary Sect. 5.4. Ratios in the range ~ 1–3 gave the highest crude RCY, with a rapid drop for lower ratio values and a gradual drop for higher values. When exploring increasing reaction time (Fig. 4D), fluorination efficiency exhibited a gradual increase and the collection efficiency exhibited an inverse trend (mostly due to volatile activity loss). The resulting crude RCY in DMF (the better performing solvent) exhibited a decrease with time, with a maximum of 15.4 ± 0.9% (n = 4) for a 0.5 min reaction. Though reaction time has not been extensively studied in the literature, longer times seemed to improve the synthesis performance in closed reactors. Ryzhikov et al*.* observed an increase in fluorination efficiency from 39% (n = 1) to 80% (n = 1) when increasing the time from 15 to 30 min^40^.

Considering the high volatile losses at high temperatures and longer reaction times, we explored additional high boiling point reaction solvents (Fig. 4E), including N-methyl-2-pyrrolidone (NMP), 1,3-dimethyl-3,4,5,6-tetrahydro-2(1H)-pyrimidinone (DMPU), and ethylene glycol, which have been used in other radiosyntheses^44,45^. Fluorination efficiency and crude RCY were significantly improved using NMP compared to DMF. As a final test we compared the influence of the type of base and phase-transfer catalyst (Fig. 4F) in the reaction solvents DMF, DMSO, and NMP. The best combination was NMP with TBAHCO_3_; much lower performance was observed with K_222_/K_2_CO_3_ and K_222_/Cs_2_CO_3_. The optimized conditions (NMP reaction solvent, 240 nmol base (TBAHCO_3_), and 280 nmol precursor in an 8 µL droplet at 200 °C for 0.5 min) resulted in fluorination efficiency of 37.5 ± 0.8 (n = 4), collection efficiency of 51 ± 1 (n = 4) and crude RCY of 19.1 ± 0.6% (n = 4). Purification via analytical HPLC (Supplementary Sect. 9.1 or a batch that had a crude RCY of 18.0% gave an isolated yield of 11.6% (n = 1). Further optimization of purification may lead to modest improvements but was not investigated. Notably, the droplet-based synthesis could achieve useful isolated yields that are only slightly below the isolated yields reported by others (Supplementary Table S11) while offering multiple advantages, including completion within only ~ 35 min (20 min for synthesis and HPLC purification, with an estimated ~ 15 min needed for formulation^46^) instead of 55–80 min ^38–40^, and 100 × reduced precursor consumption^38–40^.

### Optimization of [^18^F]PBR06 radiosynthesis

To demonstrate versatility of the high-throughput approach, we next used the platform to perform an optimization of the radiosynthesis of [^18^F]PBR06. This tracer detects microglial activation by targeting the translocator protein (TSPO) and is used for monitoring treatment response in Huntington’s disease^47^, imaging neuroinflammation, and monitoring tumor progression^48^. Using the commercially-available tosylate precursor for the radiosynthesis, isolated yields of [^18^F]PBR06 in the range 30–60% have been reported in literature^48,49^; however, to our knowledge, no studies have been reported on the influence of different reaction conditions on the radiosynthesis performance.

**Figure 5:**
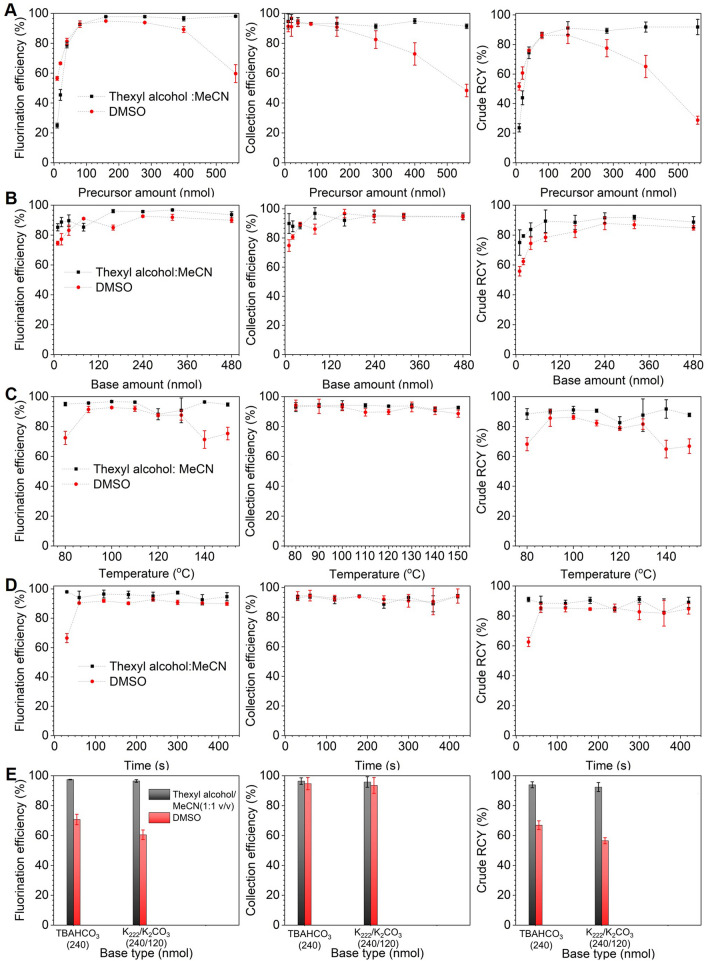
Influence of reaction parameters on the performance of the microdroplet radiosynthesis of [^18^F]PBR06. For each parameter, influence on fluorination efficiency, collection efficiency, and crude RCY are plotted individually. (**A**) Effect of precursor concentration (and solvent). Reaction volume: 8 µL. Base amount: 240 nmol. Reaction time: 5 min. Reaction temperature 100 °C. (**B**) Effect of amount of base (and solvent). Precursor amount: 160 nmol. Reaction volume: 8 µL. Reaction temperature: 100 °C. Reaction time: 5 min. (**C**) Effect of temperature (and solvent). Precursor amount: 160 nmol. Reaction volume: 8 µL. Base amount: 240 nmol. Reaction time: 5 min. (**D**) Effect of reaction time (and solvent). Precursor amount: 160 nmol. Reaction volume: 8 µL. Base amount: 240 nmol. Reaction temperature: 100 °C. (**E**) Effect of the base type. Precursor amount: 160 nmol. Reaction volume: 8 µL. Base amount: 240 nmol. Reaction temperature: 100 °C. Reaction time: 0.5 min.

Full details of all parameters we explored (precursor amount, base amount, temperature, reaction time, and type of base/phase transfer catalyst) are included in the Supplementary Sect. 6. Similarly to [^18^F]Flumazenil, studies of each parameter were performed in the following two different reaction solvents: DMSO (commonly reported in literature^48,49^), and a 1:1 (v/v) mixture of thexyl alcohol and MeCN (used in aliphatic radiofluorinations of other tosylate precursors^33^). In the study of precursor amount (Fig. 5A), reactions in the mixed solvent showed a rapidly increasing fluorination efficiency with increasing precursor amount, reaching a plateau of ~ 100% at ~ 100–200 nmol of precursor, and the collection efficiency was consistently high. The resulting crude RCY increased rapidly as precursor amount was increased, reaching a plateau of 91 ± 4% (n = 4) at 160 nmol of precursor. Interestingly, for reactions performed in DMSO, the trends were similar for precursor amounts below ~ 100–200 nmol precursor, but for higher precursor amounts, the fluorination efficiency, collection efficiency, and crude RCY showed gradual to moderate decrease instead of leveling off. Still, the maximum crude RCY using DMSO (86 ± 6%, n = 4) was similar to that obtained using the mixed solvent.

In the studies of base amount (Fig. 5B), fluorination efficiency, collection efficiency, and crude RCY were relatively unaffected, showing only modest reductions when the amount of base was lower than ~ 150 nmol. The crude RCY was maximal at 240 nmol of base. In the study of reaction temperature (Fig. 5C), the fluorination efficiency was relatively independent of temperature when using the mixed reaction solvent. When using DMSO, the fluorination was highest from 90 – 130 °C. Collection efficiency was consistently high across all temperatures (and for both solvents), and the crude RCY mirrored the fluorination efficiency. A temperature of 100 °C was selected. The reaction time (Fig. 5D) had almost no impact, with high crude RCY in all cases except for DMSO at 0.5 min, where the crude RCY was substantially lower. Finally, we found no significant difference when using TBAHCO3 compared to the typically reported phase transfer catalyst K_222_/K_2_CO_3_ when using the mixed solvent; however, when using DMSO as the reaction solvent, the fluorination efficiency and crude RCY were slightly lower when using K_222_/K_2_CO_3_ compared to TBAHCO_3_ (Fig. 5E). The results for DMSO were lower overall due to the suboptimal reaction time used in this comparison.

Overall, the optimal conditions (240 nmol of TBAHCO_3_, 160 nmol of precursor in 8 µL of thexyl alcohol: MeCN (1:1 v/v), 100 °C, 0.5 min) gave a fluorination efficiency of 97.4 ± 0.2% (n = 4) and crude RCY of 94 ± 2% (n = 4). Compared to conventional methods (Supplementary Table S18 our optimal conditions were significantly quicker (0.5 vs 15 min reaction time)^48^ and milder (100 vs 140 °C)^48^. We performed purification via analytical-scale radio-HPLC (Supplementary Sect. 9.2) and obtained an isolated yield of 75.8% (n = 1). Though we did not perform formulation to determine the overall RCY, this compares favorably with the overall RCY values (30–60%) reported in literature^48,49^, consumed 10–30 × less precursor^48,49^, and was a shorter synthesis process (~ 35 min, i.e. 20 min for synthesis and HPLC purification, plus an estimated ~ 15 min needed for formulation^46^), compared to 50 min reported in literature^49^.

### Optimization of [^18^F]Fallypride radiosynthesis

[^18^F]Fallypride is used to study diseases associated with the dopaminergic system, such as Parkinson’s, Huntington’s, and Alzheimer’s diseases^50,51^. We previously performed a preliminary study of the droplet synthesis of [^18^F]Fallypride from the tosylate precursor exploring the impact of the base amount, precursor amount, and reaction volume^33^. However, with only a single heater operating a single chip, reaction temperature and time could not previously be studied conveniently. Using the expanded capabilities of the multi-heater platform described here, we studied the impact of temperature and reaction time in combination with precursor concentration. Full details are in Supplementary Sect. 7. As a function of increasing precursor concentration, the fluorination efficiency and crude RCY exhibit a rapid increase from near zero and reach a plateau, while the collection efficiency is consistently high (Supplementary Figure S41). Surprisingly, nearly identical behavior was observed for reaction temperatures of 95, 110, and 125 °C; but at 80 °C it was evident that a higher precursor concentration was needed to achieve the maximal fluorination efficiency and crude RCY (Supplementary Figure S41A). Maximum crude RCY occurred at 110 °C and 39 mM precursor. When examining the combined effect of precursor concentration and reaction time (Supplementary Figure S41B), the impact of reaction time was nearly negligible, only leading to discernable differences when the precursor concentration was below ~ 20 mM. The highest crude RCY (93 ± 5%, n = 2) was obtained when running the reaction at 110 °C for 1.0 min, 240 nmol of TBAHCO_3_, and 39 mM precursor in 6 µL of thexyl alcohol: MeCN (1:1 v/v). After purification by analytical HPLC (Supplementary Sect. 9.3) the isolated yield was 74% (n = 1). The ability to perform a kinetic study like this in one set of simultaneous experiments is a significant advantage compared to the typical lengthy series of sequential studies with conventional instruments or microfluidic flow chemistry systems^52–54^. This approach likely also provides more reliable reaction times and temperatures than when repeatedly cooling and opening a single reaction to extract samples at different timepoints^55^.

### Optimization of [^18^F]FEPPA radiosynthesis

As a final example, we performed a very limited optimization of the synthesis of [^18^F]FEPPA, a radiopharmaceutical that has been used in several pre-clinical and clinical settings in recent years^56–60^ to look at the overexpression of TSPO, which is known to be associated with a variety of neurodegenerative disorders. With the aid of the high-throughput platform to explore the influence of temperature (Supplementary Sect. 8), we translated the synthesis into droplet format to leverage the advantages of small-volume reactions. We started with conditions similar to past droplet studies for other tracers using tosylate precursors. Since literature reports include a range of 9 to 45 mM precursor concentration^56–58,61^, we chose an initial value of 30 mM. As a function of increasing temperature (Supplementary Figure S44), the fluorination efficiency was ~ 10% at 60 °C and sharply increased to reach a plateau after 90 °C. The collection efficiency was consistently high at all temperatures, and the resulting crude RCY showed a similar trend to the fluorination efficiency. The highest crude RCY (77 ± 2%, n = 4) was observed at a temperature of 110 °C for 2.0 min, 30 mM precursor in 8 µL of thexyl alcohol: MeCN (1:1 v/v) solvent and 240 nmol of base (TBAHCO_3_). Compared to literature methods (Supplementary Table S22), the reaction time is shorter (2 min vs 10 min^56–58,61^), the droplet reaction consumes 40–50 × less precursor, and the overall synthesis is shorter and has higher yield^56–58,61^. A batch was purified by analytical-scale HPLC (Supplementary Sect. 9.4) and the collected fraction was diluted (1:3, v/v) with 9 mM NaHCO_3_ to produce an isotonic solution appropriate for injection containing 440 MBq [12 mCi], sufficient for multiple preclinical studies. The overall 30 min synthesis had an RCY of 67% (n = 1).

### Clinical-scale radiosynthesis

The optimization experiments in this work were performed with ~ 14 MBq [0.38 mCi], where each reaction often yielded enough product for multiple mouse scans^62,63^. Nevertheless, we wanted to explore whether one of the optimized compounds ([^18^F]PBR06) could be scaled to clinically-relevant levels without changing any reaction conditions other than the amount of starting radioactivity. We’ve previously reported that significant scale-up is possible for [^18^F]Fallypride (7.2 GBq demonstrated)^31^, as well as O-2-[^18^F]fluoroethyl-L-tyrosine ([^18^F]FET) and [^18^F]Florbetaben (up to 0.8 GBq demonstrated for each)^32^. Experiments with increasing starting activity up to 3.2 GBq (86 mCi) are described in the Supplementary Sect. 10. While the crude RCY showed a slight reduction as starting activity increased due to a decrease in fluorination efficiency, the final quantities after purification and formulation would still be sufficient for several clinical doses.

These results reinforce the ability to optimize small-scale reactions in a high-throughput fashion using the platform described here, and then scale up the starting activity to increase the output of a droplet radiosynthesis. In fact, the starting activity itself could be varied as a reaction parameter and studied with high throughput using the platform described in this paper. Studies are currently underway in our laboratory to explore in more detail how scale-up influences the performance.

### Comparison to other optimization approaches

With the platform presented here for performing parallel radiosyntheses in droplet format, we were able to rapidly and conveniently study the influence of various reaction parameters to obtain a detailed map of conditions that influence the synthesis performance. Each radiopharmaceutical synthesis could be extensively investigated (100s of data points) in a few days, requiring only a few batches of radioisotope. In total, for the four example compounds, 820 experiments were completed in 15 experiment days, with an average of 55 reactions per day. While the maximum number of experiments completed in a single day was 64, it is probably feasible to increase this number to ~ 96. The limiting factor is the tedious manual adding of reagents, collecting crude products, and performing TLC analysis. An automated platform for high-throughput experimentation is currently being developed, which could address these issues and perhaps increase reaction throughput further while also reducing radiation exposure and the chance for human error^64^. Performing many reactions per day saves on total time (and thus labor and other costs) for optimization and requires far fewer batches of radioisotope, significantly reducing radioisotope production and/or purchase and shipping costs. Importantly, since day-to-day variation such as radioisotope quality or reagent preparation can sometimes also affect results^65^, reducing the total number of experimental days (and radioisotope batches) also reduces the confounding effects of this variability. Furthermore, using small-scale droplet reactions (i.e., 6–8 µL) compared to conventional reactors (0.5–2.0 mL), reagent usage per datapoint was reduced by ~ 10-100x. The total amount of precursor consumed was only 30 mg for 355 data points for [^18^F]Flumazenil, 20 mg for 296 data points for [^18^F]PBR06, 6 mg for 128 data points for [^18^F]Fallypride, and 4 mg for 32 data points for [^18^F]FEPPA. These amounts are equivalent to just 12 macroscale reactions for [^18^F]Flumazenil (5 mg each), 6–7 for [^18^F]PBR06 (3 mg each), 3 for [^18^F]Fallypride (2 mg each), and one for [^18^F]FEPPA. Moreover, the amount of product activity in some cases is sufficient for in vitro or pre-clinical in vivo imaging studies. This could be a tremendous advantage for new radiotracer development where the precursor is in short supply. The droplet platform allows the possibility of performing both optimization and initial preliminary biological studies in the shortest time using only a few mg of precursor.

Aside from conventional radiosynthesizers, rapid and economical optimizations have also been performed using continuous-flow microfluidic platforms. Small boluses of reagents (10s of µL) are reacted sequentially under different conditions^20,66^ (up to 25 experiments per day have been reported^67^). While convenient for studying the influence of residence time, reactant concentrations and ratios (via changes in relative flow rates), and reaction temperature, varying other conditions (e.g. solvent) is cumbersome, requiring manual intervention and cleaning procedures for each change. In addition, some aspects (e.g. [^18^F]fluoride drying conditions) cannot be explored in a high-throughput fashion since they are performed outside the flow-chemistry workflow. Droplet reactors are suitable for studying all of these variables and can perform reactions in parallel rather than sequentially. An additional advantage of optimization using droplet reactions is that the multi-heater platform is compact (120 × 120 × 100 mm^3^), allowing operation in a small part of a hot cell or mini-cell. Its low weight (~ 900 g) makes the system portable and easy to move in and out of a hot cell and occupies space only when optimization efforts are needed. In contrast, conventional radiosynthesizers and flow chemistry systems are typically much larger and integrated into infrastructure (gases, vacuum) and cannot easily be moved.

A unique feature of the open microdroplet system is the convenience of visualizing and quantifying the radioactivity distribution on the chip surface via Cerenkov imaging at different stages of the synthesis process. This information enables a more comprehensive assessment of the performance of each step. While some macroscale systems include radiation detectors near reaction vessels and cartridges that enable straightforward activity measurements on these components to help pinpoint losses^43^, other systems require removing/disassembling components to make activity measurements, which may be infeasible or inconvenient and increases radiation exposure. In comparison, this data can be readily gathered in parallel for many reactions at once in our high-throughput approach, saving significant time, reducing radiation exposure, and reducing the chance for errors.

On the other hand, a limitation of this approach is that the open droplet format had significant volatile losses for some syntheses. While volatile losses were very low for [^18^F]PBR06, [^18^F]Fallypride, and [^18^F]FEPPA in droplet format (as well as many other tracers^28,46,68^), losses were significant for [^18^F]Flumazenil and were found to occur during the radiofluorination step. In contrast, in macroscale systems, the reactor is usually closed for the duration of the reaction, and losses during this step are generally likely to be lower. Of course, both droplet systems and conventional systems can exhibit volatile losses at other stages of the radiosynthesis process, such as during solvent evaporation steps. Despite the volatile loss, meaningful and repeatable experiments could still be performed. Moreover, the isolated yield for [^18^F]Flumazenil (which had substantial volatile loss) was only slightly below the range of isolated yields (before formulation) reported by others, suggesting that loss of the volatile species was not very detrimental to the overall reaction performance, or perhaps that the reaction loss was offset by other improvements (e.g. perhaps the use of an analytical instead of semi-preparative HPLC column reduced the degree of purification loss). Of course, the volatile losses present a hazard that needs to be mitigated by operation of the system within an appropriate hot cell.

While studies here were performed using a one variable at a time (OVAT) method, further optimization efficiency improvements might be achieved by integrating concepts like the design of experiments (DoE)^65^ and reaction modeling. In addition to the reactions optimized here, the droplet format is compatible with other ^18^F-labeled radiopharmaceuticals^28,29,46,69^. It can likely be used with other isotopes, including radiometals. Although designed for operation in a radiochemistry laboratory, the platform could possibly also be used for reagent-economical optimization of a wide range of chemical reactions outside the field of radiochemistry. Recently, several new platforms and techniques have been reported for performing screening of organic reactions in volumes of 1.5–100 µL^15,16^, and our platform could provide an enhanced ability to vary reaction temperatures and times for different simultaneous reactions.

## Conclusion

In this work, we have developed a platform for radiosynthesis optimization relying on droplet-based reaction arrays that enables many reactions (up to 64) to be performed in parallel, each with minimal reagent consumption. Combined with high-throughput analysis methods^34^, it is practical to perform hundreds of experiments in a matter of days. While similar in throughput to flow-chemistry-based optimization methods^70^, this platform allows studies of all stages of the synthesis process, including [^18^F]fluoride drying/activation, and it has been shown in previous work that the chips are also compatible with optimization of reactions having at least 2 synthetic steps^29,46^. It also allows reaction solvent and reagent amounts to be readily varied without the constraints of flow-based systems. Finally, product amounts can be scaled up after optimization by varying the starting activity.

As examples, we used the platform to perform the rapid optimization of the production of [^18^F]Flumazenil, [^18^F]PBR06, [^18^F]Fallypride, and [^18^F]FEPPA from their commercially-available precursors. Using the platform, a series of syntheses using different conditions (85 for [^18^F]Flumazenil, 74 for [^18^F]PBR06, 64 for [^18^F]Fallypride, and 8 for [^18^F]FEPPA), spanning 6 different reaction parameters, were performed. Replicate studies were performed for each condition and the small standard deviation computed for each set of replicates indicated that the platform has high reproducibility. For [^18^F]Flumazenil, the observed trends were comparable to optimization studies performed using conventional radiosynthesizers. For other tracers there is limited optimization data in the literature.

This platform conveniently brings the power and efficiencies of high-throughput experimentation to the field of radiochemistry. It could find use in: (i) rapid refinement and optimization of radiosynthesis protocols for existing or novel radiopharmaceuticals, (ii) translation of known macroscale protocols into droplet format, and (iii) studies of novel labeling methods. The high throughput platform allows exploration of many more reaction conditions within the available parameter space, which can potentially lead to discovery of favorable reaction conditions that might not otherwise be attempted with conventional methods due to time, cost, and low throughput. The small amount of precursor required for each reaction is a crucial advantage, particularly in the early stages of novel radiopharmaceutical development where only a small amount of the starting material may be available; the high-throughput platform enables the development of syntheses within a short timeframe at low cost.

## Materials and methods

### Materials

Anhydrous N,N-dimethylformamide (DMF, 99.8%) purchased from Fisher Scientific, anhydrous dimethyl sulfoxide (DMSO, ≥ 99.9%), anhydrous acetonitrile (MeCN, 99.8%), 2,3-dimethyl-2-butanol (thexyl alcohol,98%), 4,7,13,16,21,24-hexaoxa-1,10-diazabicyclo[8.8.8]hexacosane (K_222_, 98%), triethylamine (TEA, 99%), trifluoroacetic acid (TFA, > 99%), tetrahydrofluran (THF, > 99.9%, inhibitor-free), hexanes (95%), dichloromethane (DCM, > 99.8%), acetone (99.5%), ammonium formate (NH_4_HCO_2_: 97%) N-methyl-2-pyrrolidone (NMP, 99.5% anhydrous), 1,3-dimethyl-3,4,5,6-tetrahydro-2(1H)-pyrimidinone (DMPU, 98%), ethylene glycol (99.8%) and potassium carbonate (K_2_CO_3_, 99.995%) were purchased from Sigma-Aldrich (St. Louis, MO, USA). n-butanol (nBuOH, 99%) was purchased from Alfa Aesar (Ward Hill, MA, USA). Tetrabutylammonium bicarbonate (TBAHCO_3_, 75 mM in ethanol), ethyl-5-methyl-8-nitro-6-oxo-5,6-dihydro-4*H*-benzo[f]imidazo[1,5-*a*][1,4]diazepine-3-carboxylate (nitromazenil; precursor for [^18^F]Flumazenil, > 97%) and Flumazenil (reference standard, > 99%), 2-((2,5-dimethoxybenzyl)(2-phenoxyphenyl)amino)-2-oxoethyl-4-methylbenzenesulfonate ([^18^F]PBR06 precursor, > 95%), 2-fluoro-N-(2-methoxy-5-methoxybenzyl)-N-(2-phenoxyphenyl)acetamide (reference standard for [^18^F]PBR06, > 95%), (*S*)-2,3-dimethoxy-5-[3-[[4-methylphenyl)-sulfonyl]oxy]-propyl]-N-[[1-(2-propenyl)-2-pyrrolidinyl]methyl]benzamide ([^18^F]Fallypride precursor, > 90%), Fallypride (reference standard, > 95%), 2-(2-((*N*-4-phenoxypyridin-3-yl)acetamido)methyl)phenoxy)ethyl-4-methylbenzenesulfonate ([^18^F]FEPPA precursor, > 90%), and *N*-[[2-(2-fluoroethoxy)phenyl]methyl]-*N*-(4-phenoxypyridin-3-yl)acetamide (reference standard for [^18^F]FEPPA, > 95%) were purchased from ABX Advanced Biochemical Compounds (Radeberg, Germany). DI water was obtained from a Milli-Q water purification system (EMD Millipore Corporation, Berlin, Germany). No-carrier-added [^18^F]fluoride in [^18^O]H_2_O was obtained from the UCLA Ahmanson Biomedical Cyclotron Facility and Crump Cyclotron Facility.

1% Teflon AF 2400 solution was purchased from Chemours. Positive photoresist (MEGAPOSIT SPR 220–7.0) and developer (MEGAPOSIT MF-26A) were purchased from MicroChem (Westborough, USA). Additional solvents and chemicals used for microfluidic chip fabrication, including methanol (MeOH, Cleanroom LP grade), acetone (Cleanroom LP grade), isopropanol (IPA, Cleanroom LP grade), sulfuric acid (96%, Cleanroom MB grade) and hydrogen peroxide (30%, Cleanroom LP grade), were purchased from KMG Chemicals (Fort Worth, USA).

The following stock solutions were prepared daily to carry out droplet reactions. The [18F]fluoride stock solution contained either 60 mM TBAHCO3 and 1.8 MBq/μL (48 μCi/μL) of [18F]fluoride in water (i.e. for [18F]Flumazenil, [18F]PBR06, [18F]Fallypride, and [18F]FEPPA), or 60 mM of K222 with 30 mM of K2CO3 and 1.8 MBq/μL (48 μCi/μL) of [18F]fluoride in water (i.e. for [18F]Flumazenil and [18F]PBR06), or 60 mM of K222 with 30 mM of Cs2CO3 and 1.8 MBq/μL (48 μCi/μL) of [18F]fluoride in water (i.e. for [18F]Flumazenil). [18F]Flumazenil precursor stock solution contained 70 mM precursor in either DMSO, DMF, NMP, DMPU, or ethylene glycol. [18F]PBR06 precursor stock solution contained 70 mM precursor in either DMSO or a 1:1 v/v mixture of thexyl alcohol and MeCN. [18F]Fallypride stock solution contained 77 mM of precursor in a 1:1 v/v mixture of thexyl alcohol and MeCN. [18F]FEPPA stock solution contained 30 mM of precursor in a 1:1 v/v mixture of thexyl alcohol and MeCN. For [^18^F]Flumazenil, the collection stock solution was a 2:1 v/v mixture of reaction solvent and water when using DMSO or DMF as the reaction solvent, or a 9:1 v/v mixture of MeOH and H_2_O collection stock solution when using NMP, DMPU, or ethylene glycol as the reaction solvent. For [^18^F]PBR06, [^18^F]Fallypride, and [^18^F]FEPPA, the collection stock solution was a 9:1 v/v mixture of MeOH and H_2_O.

### Analytical methods

#### Analysis of reaction performance

Radioactivity measurements were made using a calibrated dose calibrator (CRC-25R, Capintec, Florham Park, NJ, USA). To calculate the starting activity on each reaction site, we measured the activity on the chip after loading the initial [^18^F]fluoride solution to each individual spot (via dose calibrator) and subtracted the previous measurement of chip activity. All measurements were decay-corrected to a common timepoint. Collection efficiency was determined by dividing the activity of the collected crude sample from an individual spot by the starting activity used in that same reaction site (corrected for decay). Fluorination efficiency was analyzed using radio-TLC. Crude radiochemical yield (crude RCY) was calculated by multiplying the collection efficiency by the fluorination efficiency. The total residual activity left on the chip was measured by placing the chip in a dose calibrator after collection of the crude products from each reaction site. To compute the residual activity left on the chip at each individual reaction site, the activity distribution on the chips was first determined via Cerenkov imaging^33,68,71^. For Cerenkov imaging, a glass microscope slide (76.2 mm × 50.8 mm, 1 mm thick; C&A Scientific; Manassas, VA, USA) was placed on top of the chip, and the acquisition time was 5 min. Raw images were corrected as previously described^34^. Residual activity for a particular reaction site on the chip was computed with the aid of a custom region-of-interest (ROI) analysis software written in MATLAB (MathWorks, Natick, MA). For each reaction site, an ROI was drawn and the integrated Cerenkov signal was computed from the image. To quantify the amount of residual activity at a particular reaction site, the corresponding ROI integrated signal was divided by the sum of integrated signal for all ROIs and multiple by the measured total residual radioactivity on the chip. This value could then be expressed as a fraction of starting radioactivity by dividing the residual activity for a particular reaction site by the starting activity used at that particular reaction site (correcting for decay).

#### Thin-layer chromatography

Performing 64 simultaneous reactions presents a significant challenge for analysis. Typical methods of spotting one sample per TLC plate for typically used TLC plates lengths and conditions require 2–7 min per sample separation and readout and cannot be practically scaled to 64 samples. To accelerate the analysis, TLC plates (silica gel 60 F254; Merck KGaA, Darmstadt, Germany) were spotted with multiple samples (8 samples at 0.5 mm pitch), with all samples separated in parallel and read out simultaneously via Cerenkov imaging using methods we have previously reported^34^. Briefly, 8 samples (0.5 μL each) were spotted onto a 50 mm × 60 mm (W x L) TLC plate, with adjacent spots separated by 5 mm. Developed TLC plates were read out by covering the TLC plate with a scintillator plate (50 mm × 35 mm, 1 mm thick, BC-400, Saint-Gobain, OH, USA) or a glass microscope slide (76.2 mm × 50.8 mm, 1 mm thick, A&C Scientific) to obtain images of the emitted light. The solvent front took ~ 2 min to travel 45 mm (corresponding to 30 mm separation distance). The mobile phase to separate the [^18^F]Flumazenil crude sample was 100% MeCN, for [^18^F]PBR06 crude samples 13:10:24:54 (v/v) dichloromethane:chloroform:acetone:hexanes as the mobile phase, for [^18^F]FEPPA crude samples 25.6:37.5:36.5:0.4 (v/v) nBuOH:THF:hexanes:TEA as the mobile phase, and [^18^F]Fallypride crude samples were separated using 60% MeCN in 25 mM HN_4_HCO_2_ with 1% TEA (v/v), as previously reported^33^. More information on R_f_ values and TLC separation studies can be found in Supplementary Sect. 4.

#### High-performance liquid chromatography (HPLC)

Analytical radio-HPLC was used to identify the product of each synthesis (via co-injection with reference standard) and to isolate pure products to confirm the R_f_ value of the product bands in radio-TLC. The radio-HPLC system setup comprised a Smartline HPLC system (Knauer, Berlin, Germany) equipped with a degasser (Model 5050), pump (Model 1000), UV detector (254 nm; Eckert & Ziegler, Berlin, Germany) and gamma-radiation detector, and counter (B-FC- 4100 and BFC-1000; Bioscan, Inc., Poway, CA, USA). All HPLC separations used a C_18_ Gemini column (Kinetex, 250 × 4.6 mm, 5 µm, Phenomenex, Torrance, CA, USA). Using a mobile phase of 3:1 H_2_O:MeCN with 0.1% TFA (v/v) and a flow rate of 1.0 mL/min, the retention time of [^18^F]Flumazenil was 11 min. For [^18^F]PBR06, the retention time was 8 min using a mobile phase of 60:40 (v/v) MeCN:20 mM sodium phosphate buffer (pH = 5.8) with a flow rate of 1.5 mL/min. [^18^F]Fallypride samples were separated with a mobile phase of 60% MeCN in 25 mM HN_4_HCO_2_ with 1% TEA (v/v) and a flow rate of 1.5 mL/min resulting in a retention time of 4.5 min. [^18^F]FEPPA samples were separated with a mobile phase of 70:30 v/v H_2_O:EtOH with 0.1% H_3_PO_4_ at 0.8 mL/min, giving a retention time of 15.5 min.

## Supplementary Information

Supplementary Information.
