# Supplementary material for: Microliter-scale reaction arrays for economical high-throughput experimentation in radiochemistry
Source: Sci Rep. 2022 Jun 17;12:10263. doi: 10.1038/s41598-022-14022-2 (PMC9205965; doi:10.1038/s41598-022-14022-2)
Supplement: Supplementary file 1 — Supplementary Information. [file 41598_2022_14022_MOESM1_ESM.pdf]

Supplemental Information

Microliter-scale reaction arrays for economical, high-throughput experimentation in radiochemistry

Alejandra Rios[1,6], Travis S. Holloway[2,6], Philip H. Chao[3,6], Christian De Caro[4,6], Chelsea C. Okoro[5,6], and R. Michael van Dam[1,2,3,6*]

[1] Physics and Biology in Medicine Interdepartmental Graduate Program, University of California Los Angeles (UCLA), Los Angeles, CA, USA
[2] Department of Molecular & Medical Pharmacology, David Geffen School of Medicine, UCLA, USA
[3] Department of Bioengineering, UCLA, USA
[4] Department of Physics & Astronomy, UCLA, USA
[5] Institute for Society and Genetics, UCLA, USA
[6] Crump Institute for Molecular Imaging, UCLA, USA
[*] Corresponding author: mvandam@mednet.ucla.edu

Table of Contents

1. Multi-heater platform 3
2. Thermal simulations 3
3. Heater calibration and characterization 7
4. Radio-TLC Methods 11
5 Optimization of [^{18}F]Flumazenil synthesis 12
 5.1 Reaction temperature and solvent 12
 5.2 Base amount and solvent 13
 5.3 Precursor amount and solvent 15
 5.4 Effect of base to precursor ratio 18
 5.5 Reaction time and solvent 19
 5.6 Further studies of reaction solvent and temperature 21
 5.7 Base type and solvent 24
 5.8 Comparison to literature methods 27
6 Optimization of [^{18}F]PBR06 synthesis 28
 6.1 Precursor amount and solvent 28
 6.2 Base amount and solvent 30
 6.3 Reaction temperature and solvent 32
 6.4 Reaction time and solvent 34
 6.5 Base type and solvent 36
 6.6 Additional studies of reaction temperature 38

6.7 Comparison to literature methods 39
7 Optimization of [^{18}F]Fallypride synthesis 40
7.1 Precursor concentration and reaction temperature 40
7.2 Precursor concentration and reaction time 42
8 Optimization of [^{18}F]FEPPA synthesis 44
8.1 Reaction temperature 44
8.2 Comparison to literature methods 47
9 Representative chromatograms 48
9.1 [^{18}F]Flumazenil 48
9.2 [^{18}F]PBR06 48
9.3 [^{18}F]Fallypride 49
9.4 [^{18}F]FEPPA 49
10 Clinical-scale radiosynthesis 50
11 References 51

1. Multi-heater platform

The platform was assembled as described in the manuscript from the following components: ceramic heaters (Ultramic CER-1-01-00093, Watlow, St. Louis, MO, USA), , epoxy glue (JB weld, Sulphur Springs, TX, USA), calcium silicate thermal insulation material (McMaster-Carr; Atlanta, GA), 3D-printed nylon piece (Fictiv Inc., San Francisco, CA, USA), DC fans (Sanyo Denki model 9GV3612G301), thermocouple amplifier board (AD8495 Breakout Board, Adafruit, New York, NY, USA), data acquisition module (DAQ; NI USB-6003, National Instruments, Austin, TX, USA), and solid-state relay (SSR, Model 240D05, Sensata-Crydom). Thermal paste (OT-201-2, OMEGA, Norwalk, CT, USA) was used to enhance thermal conductivity between the heaters and the multi-reaction chips.

Figure S1 shows a photograph of the entire platform composed of the high-throughput microdroplet apparatus and a separate control box (250 mm x 204 mm x 200 mm) that can be placed outside the shielding or hot cell. CAD models of the platform will be provided upon request.

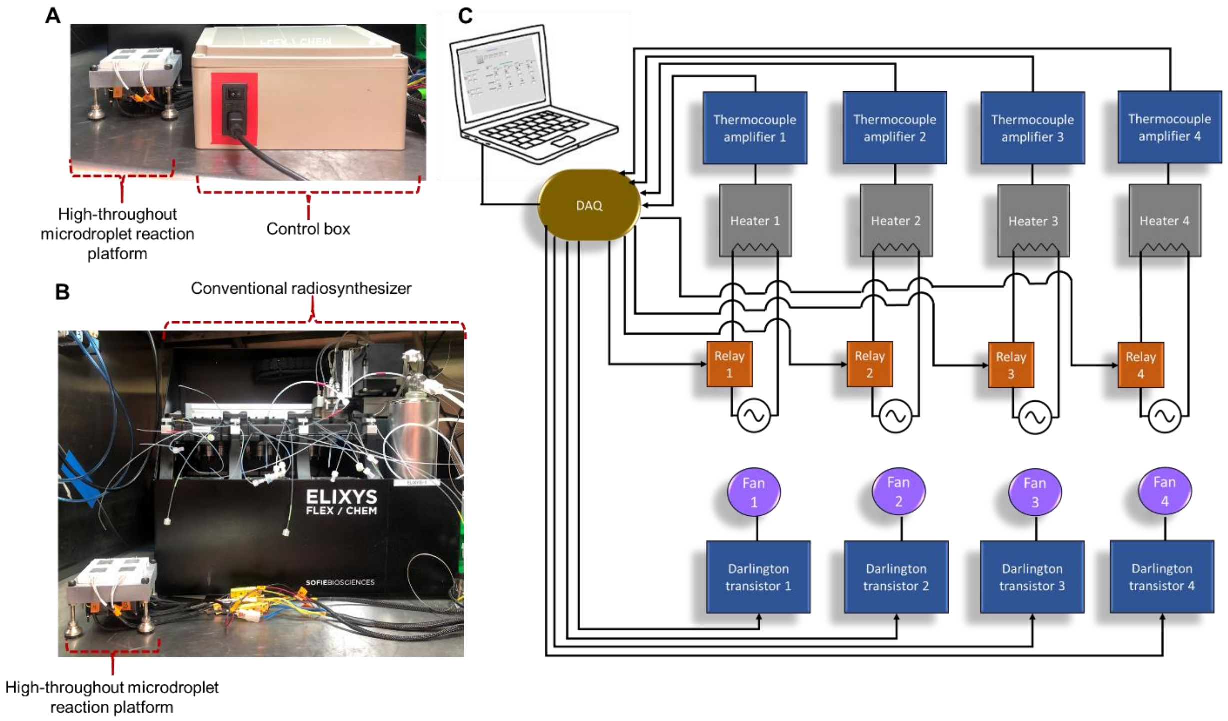

Figure S1. (A) Photograph of fully assembled high-throughput apparatus with control box inside a hot cell. (B) Photograph with control box located outside the cell to illustrate the small system footprint compared to a conventional radiosynthesizer (ELIXYS, Sofie, Inc., Culver City, CA, USA). C) Electronic wiring diagram of the high-throughput platform.

2. Thermal simulations

To prevent thermal crosstalk between heaters (i.e., one heater affecting the temperature of a neighboring heater), the heaters were mounted to a frame made of thermally insulating material. Two different types of thermal insulation were explored: Thermo-12 Gold (Johns Manville, Brunswick, GA, USA) and Firetemp

– X (Johns Manville). We chose to use Firetemp-X as it could be readily machined, in contrast to Thermo-12 Gold, which we found too flaky and fibrous. To understand if the spacing of the heaters (19.3 mm gap) was enough to prevent thermal crosstalk, thermal modeling was performed on a simplified CAD model of the platform using Solidworks (Dassault Systems, Vélizy-Villacoublay France) with the Solidworks Simulation add-in. The model includes the thermal insulation frame and the heaters. The heaters were modeled as aluminum nitride blocks having a thermal conductivity of 285 W/m-K. The frame was modeled as Firetemp–X; according to manufacturer specifications, the thermal conductivity of this material depends on operating temperature, so we chose a value of 0.094 W/m-K, which corresponds to an estimated operating temperature range of 60-150°C. The model included a thin layer (200 μm) of JB Weld epoxy between the heaters and insulating material, with thermal conductivity of 0.2 W/m-K. A thermal resistivity value of the interface between the heater and the insulation was set to be 0.001 m^2K/W. This value is derived from both the thermal conductivity of the epoxy and the contact area (allowing for heat transfer) of the heater and insulating material. Bulk ambient temperature and starting temperatures in the model were set to 298 K, and the convective coefficient for stagnant air was modeled as 25 W/m^2-K. A convection boundary condition using the convective coefficient listed above was applied to all outward-facing surfaces. Each heater was defined to have a heater power of 150 W. A high-quality mesh was applied to the model resulting in 17239 nodes and 9796 total elements.

A simple steady-state thermal simulation was performed to analyze the overall temperature distribution of the construct with each of the four heaters set to different temperatures. The resulting temperature distribution from the simulation can be seen in **Figure S2**.

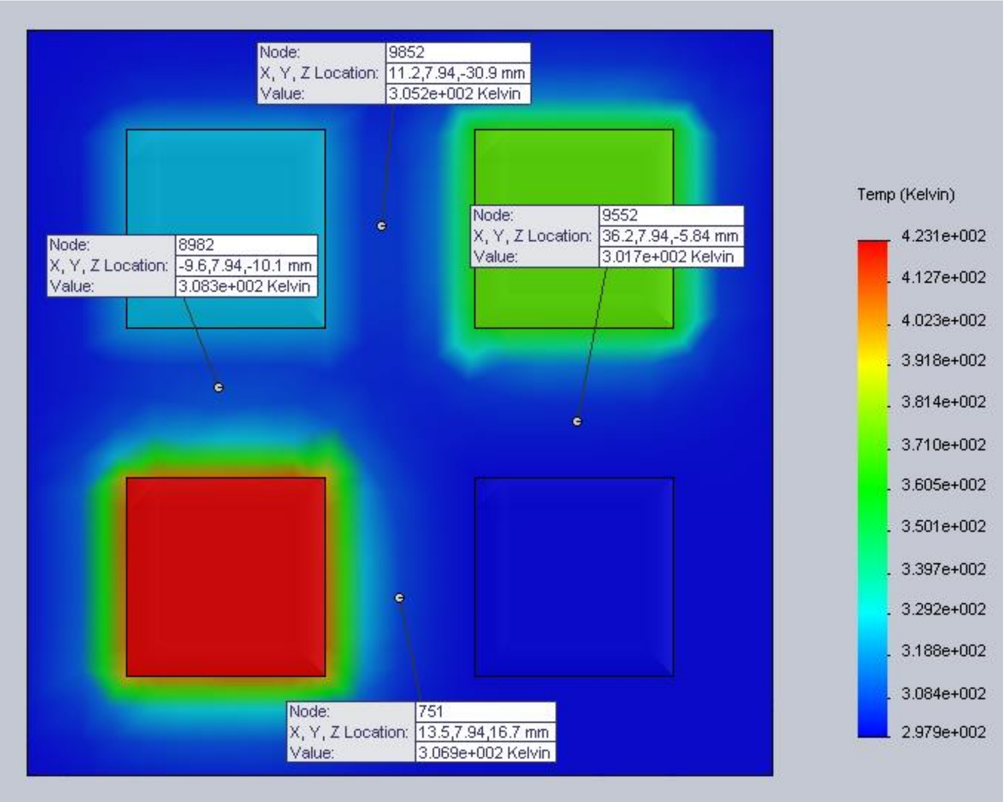

Figure S2: Steady-state temperature distribution of the four-square heaters mounted in the insulating construct, with setpoints of 323, 373, 298, and 423 K (clockwise, starting at top left). The annotated probe points represent approximate midpoints between adjacent heaters.

The simulation shows that with the designed spacing, heaters are not affected by their neighbors, even for the room temperature (298 K) heater operated adjacent to the hottest (423 K) heater. When probing the insulating material between different pairs of heaters, the highest temperature increase at the midpoint was 10.3 K (between 423 K and 323 K heaters), and the lowest was 3.7 K (between 373 K and 298 K heaters). The temperature change of the insulating material right near the edge of the heater set to 298K was zero confirming adequate insulation of the heater from neighboring heaters

The model results were verified empirically on the heating platform through thermal imaging. The heaters on the heating platform were set to different temperatures, and the system was allowed to reach a steady-state. In this study, heaters 1 to 4 (i.e., counterclockwise, starting from the top right) were set to 373K, 323K, 413K, and 305K, respectively. The thermal IR plot can be seen in **Figure S3**. Positions probed between adjacent heaters on the insulating material show a maximum increase of 5.1 K compared to the temperature of the heater set to the lowest temperature (305 K), suggesting that the insulating material can adequately prevent thermal crosstalk.

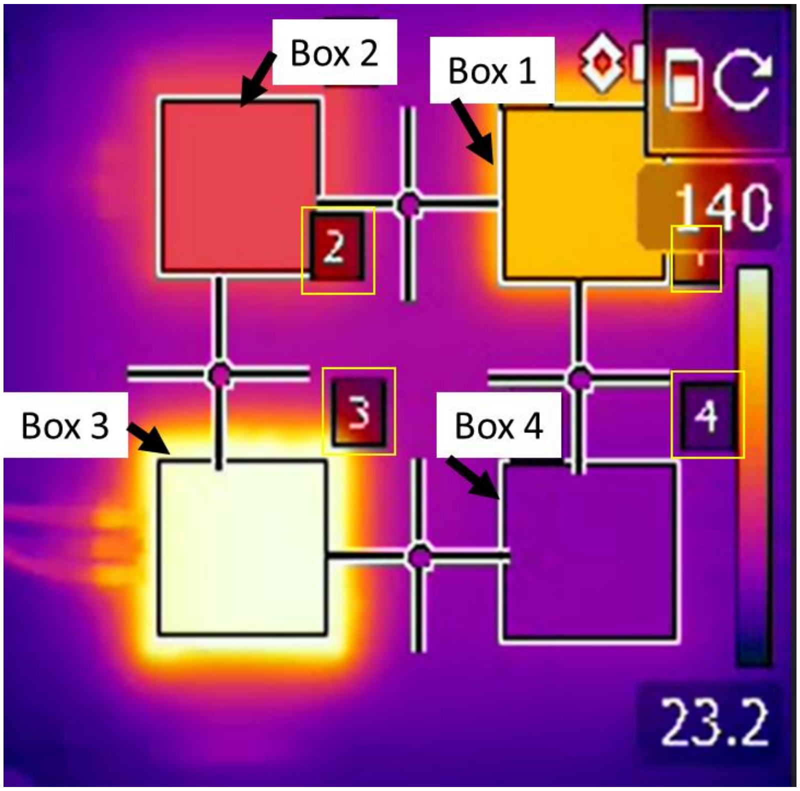

Figure S3: Thermal image of the heater platform. Box 1 through 4 corresponds to areas within heaters 1 through 4. Locations of spot probes between heaters are also shown.

Following the initial steady-state simulation, a transient simulation was performed to estimate the cooling time required for the system to reach room temperature. The model was set up similar to that of the static heating model mentioned above with some minor modifications. The transient model was performed over 600 s with a 1 s step size. Different simulations were performed using different starting temperatures (413 K, 373 K, 323 K) applied to all heaters. The bulk ambient temperature was set to 298 K. To mimic active cooling using fans, a convective coefficient of 200 W/m^2-K was applied to all surfaces in contact with the cooling airflow. The surfaces are highlighted in **Figure S4** by the green cones**.** All the

other exposed surfaces were set with a convective coefficient for stagnant air modeled as 25 W/m^2-K. A solid mesh was applied consisting of 21594 nodes and 12641 elements.

For each simulation, temperature readings positioned at the center of each heater were measured as a function of cooling time. Due to symmetries in the geometry, all heaters behaved identically. Cooling temperature profiles for heater 1 as a function of different starting temperatures can be seen in **Figure S5**. The required cooling time (to 303K) decreases as we decrease the starting temperature. For starting temperatures of 413K, 373K, and 323K, the cooling times were 108 s, 93 s, and 55 s, respectively. Empirical performance (presented in the main paper) was found to have a slower cooling time than the simulation.

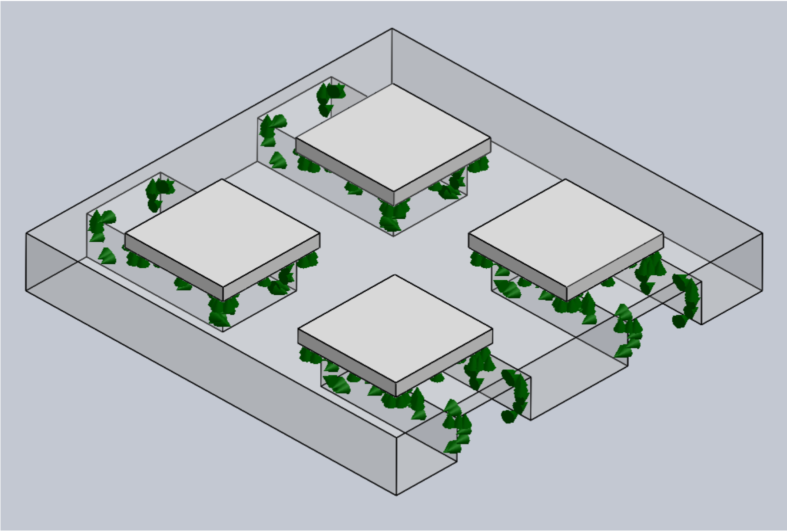

Figure S4: CAD model of the insulating material with embedded heaters. The green cones indicate the surfaces affected by active fan cooling.

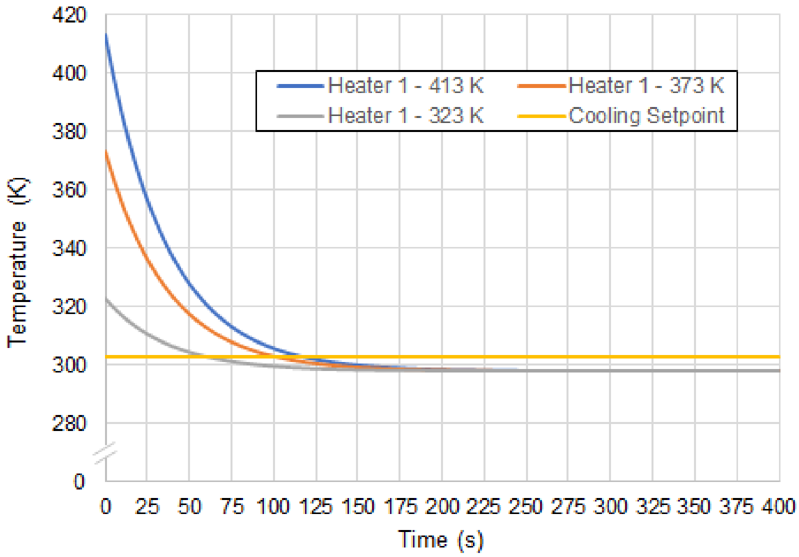

Figure S5: Simulated cooling temperature profiles as a function of time for different starting temperatures. The cooling setpoint temperature is plotted as a yellow line to visualize better when this setpoint temperature has been reached.

3. Heater calibration and characterization

We initially performed a 2-point linear calibration of thermocouple signal versus temperature by submerging each heater in 2 different water baths (ice water: 0°C; boiling water: 100°C) and measuring the output voltage from the corresponding thermocouple amplifier. Water baths were prepared in 500 mL glass beakers with stir bars, and the temperature was measured independently with a calibrated digital thermometer (53 II B, Fluke, Everett, WA, USA). The temperature stability of the heaters was assessed by setting each heater to a set temperature (50, 100, and 140 °C) and observing the integrated thermocouple measurement for 5 min (**Figure S6**). Temperature data were recorded every 0.5 s using the DAQami program (National Instruments) and plotted to examine the heating rate, cooling rate, and temperature stability at the setpoint.

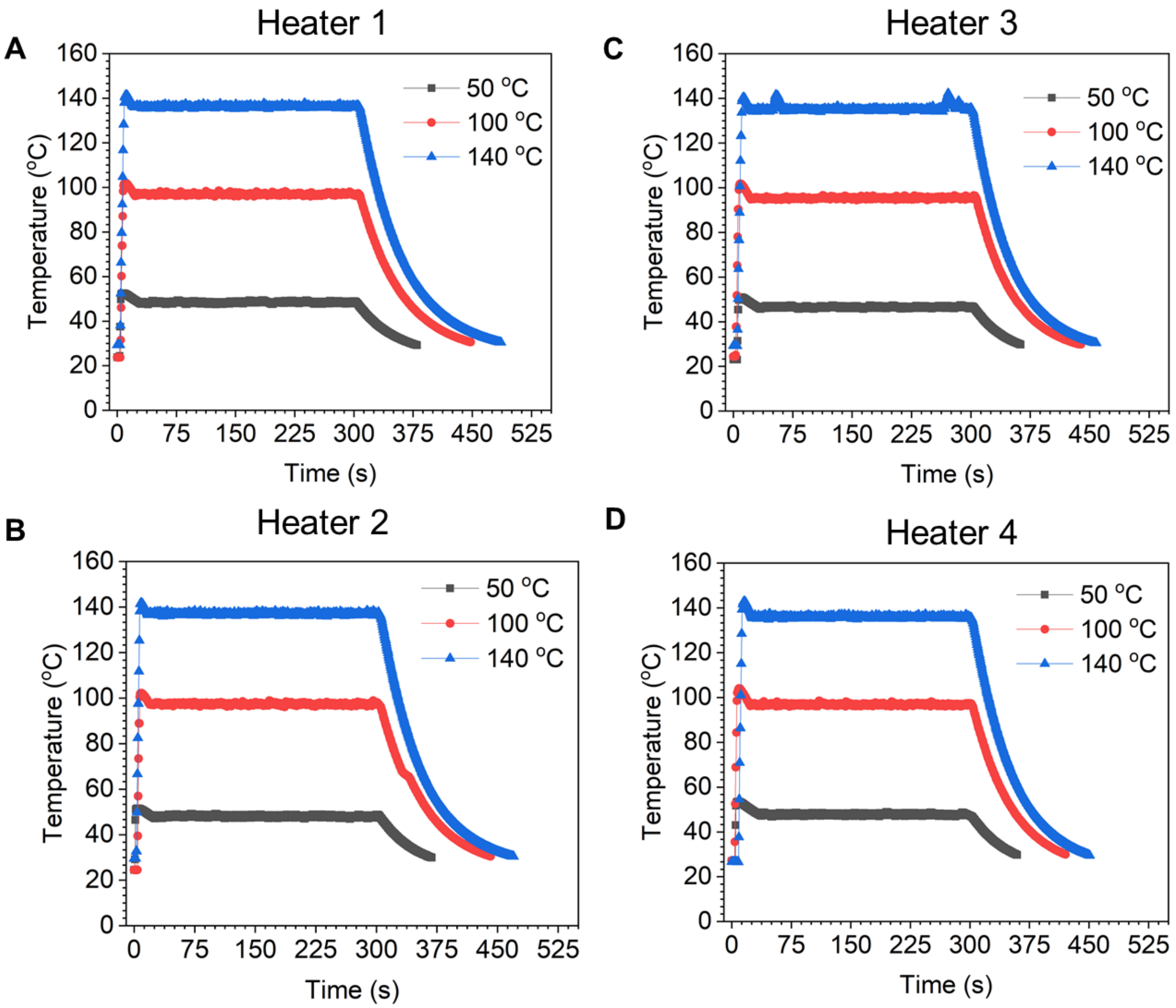

Figure S6 Temperature stability of the four heaters at three different temperatures. In each case, the heater was activated, and once it reached the setpoint, it was maintained at that temperature for 5 min, followed by forced-air cooling. (A) Heater 1; (B) Heater 2; (C) Heater 3; (D) Heater 4.

To improve accuracy, a 3-point linear calibration was later performed using a thermal camera (T440-25, FLIR, Wilsonville, OR, USA) to measure the average temperature on the heater surfaces (set at 50, 100,

140 °C, using the original calibration). For each thermal image, the temperature was allowed to stabilize for 5 min before recording the image. The spatial uniformity of temperature distribution was assessed via thermal imaging after this final calibration. **Table S1** shows the average thermocouple reading and standard deviation for the plots in **Figures S7** and **S8**. Average and standard deviation were computed from the 5 min region where the temperature had stabilized. All heater temperatures exhibited a standard deviation of <1 °C over time.

Table S1. Average heater temperatures during 5 min heating

	**Average ± standard deviation (°C)**				
**Setpoint (°C)**	**Heater 1**	**Heater 2**	**Heater 3**	**Heater 4**	**All Heaters**
**50**	48.5 ± 0.4	48.1 ± 0.5	46.6 ± 0.5	47.9 ± 0.6	47.8 ± 0.8
**100**	97.0 ± 0.5	97.4 ± 0.5	95.4 ± 0.4	96.9 ± 0.5	96.7 ± 0.9
**140**	136.3 ± 0.5	137.1 ± 0.5	135.5 ± 1.4	136.0 ± 0.5	136.2 ± 0.7

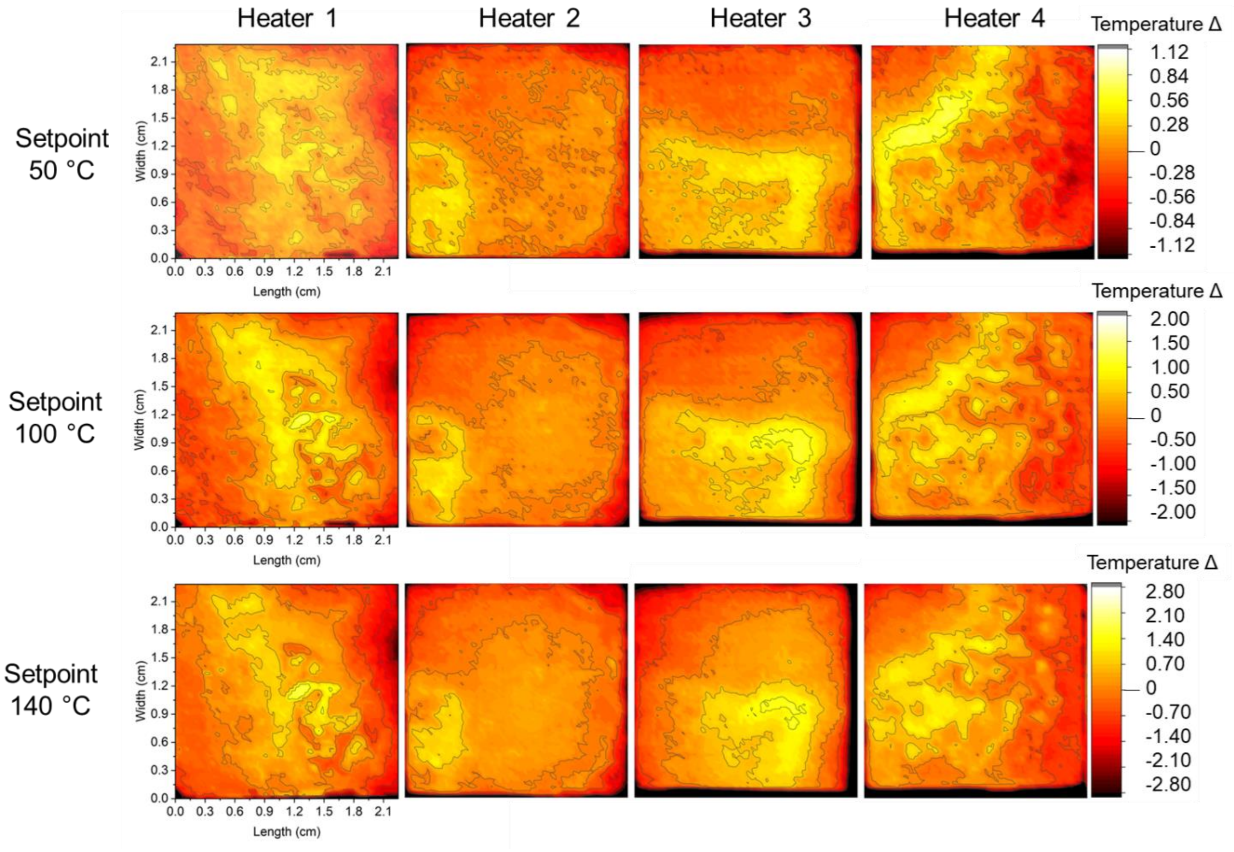

Figure S7 Thermal images of all four ceramic heaters (columns) surface at three different temperature setpoints (rows). The color represents the deviation of each pixel from the mean temperature. The dark areas show the pixels that deviate by >2% from the mean.

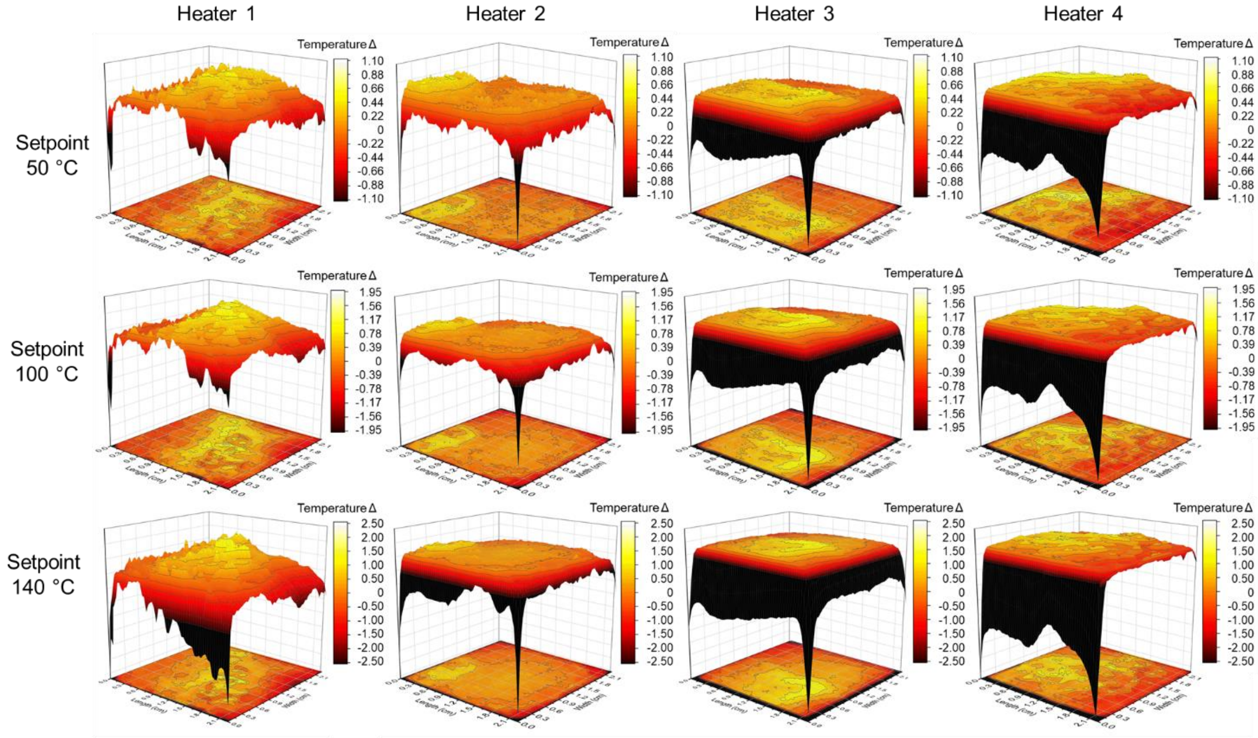

Figure S8. The same data as **Figure S7** was replotted in 3D to provide a different illustration of uniformity.

Regions of the heater with deviations >2% were considered to be unusable. **Table S2** summarizes the size of unusable regions of each heater at different set temperatures. The maximum width of the unusable region was 1.5 mm across all heaters and temperature setpoints. The maximum fraction of unusable heater surface was 8.3% (always at the edges).

Table S2. Summary of unusable regions of heaters, defined as areas where temperature value deviated >2% above or below the mean temperature of each heater (computed from thermal images).

	**Unusable fraction of heater area (%)**				**Max. width of unusable area (mm)**			
**Setpoint (°C)**	**Heater 1**	**Heater 2**	**Heater 3**	**Heater 4**	**Heater 1**	**Heater 2**	**Heater 3**	**Heater 4**
**50**	0.27	0.43	3.88	4.01	0.38	0.38	0.76	1.15
**100**	0.33	0.98	6.85	3.90	0.38	0.38	1.15	1.14
**140**	1.01	2.81	8.27	4.89	0.38	0.76	1.52	1.52

Since the maximum width of the unusable region was 1.5 mm, we designed the multi-reaction chips such that the outermost 2.4 mm border was unused, and all reaction sites were entirely located within the usable portion of the heater surface. **Figure S9** shows the detailed chip design. Chips are installed onto the heater platform in the orientations shown in **Figure S10**.

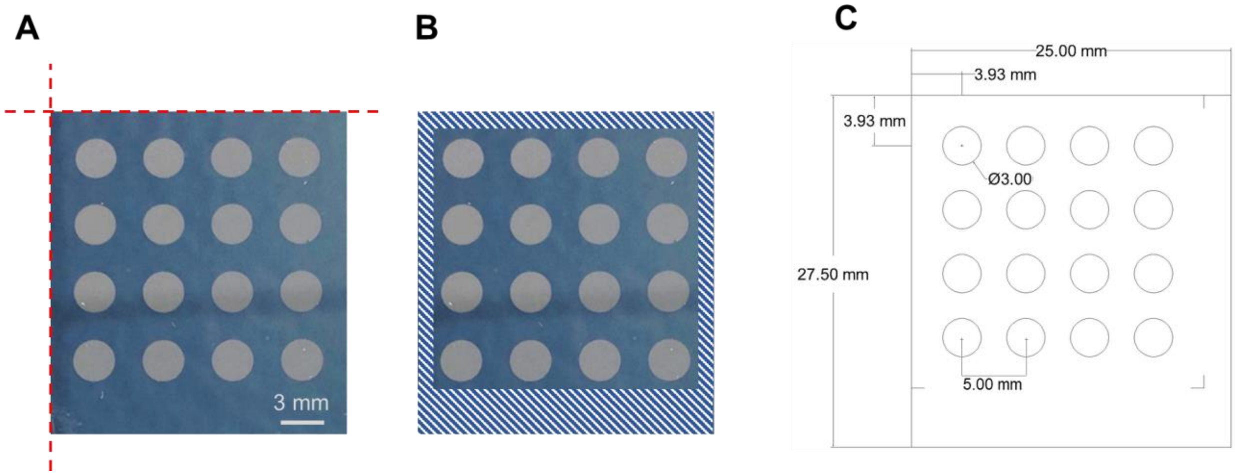

Figure S9: (A) Photograph of the chip with 16 reactions sites. The chip is made from silicon coated with Teflon AF, and then the Teflon AF layer is etched away in the circular regions. (B) The hatched region shows the unusable region of the chip (due to >2% temperature deviation from the mean temperature of the heater in these regions). (C) Detailed chip design.

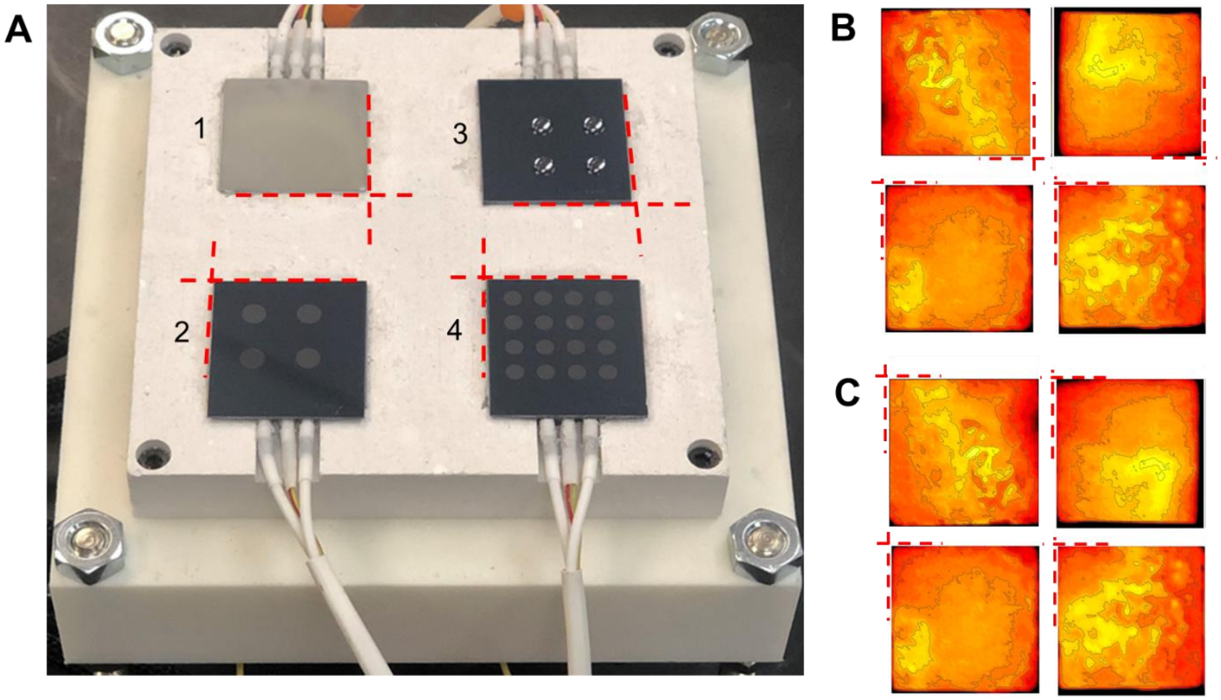

Figure S10: (A) Orientation of heaters. Red dashed lines indicate the reference corner for the alignment of chips to heaters. (B) Example thermal images from heater surfaces, shown in the actual heater orientation. (C) Thermal images in this paper are all rotated such that reference corner is located at the top left of each image.

4. Radio-TLC Methods

In order to use high-throughput radio-TLC for analysis of [^{18}F]Flumazenil, we first investigated different types of TLC plates (normal and RP-18 versions of silica gel 60 F_{254}, Merck KGaA Darmstadt, Germany) and mobile phases from literature[1–4] (**Figure S11**). Crude samples were prepared in DMSO:water (2:1 v/v), and 1 μL samples were deposited on the TLC plates. Bands were identified by comparing their R_f values with the R_f values for samples of isolated (via HPLC purification) product. The best separation between [^{18}F]fluoride, [^{18}F]Flumazenil, and an unknown impurity was achieved using (normal) silica gel 60 F_{254} plates with 100% MeCN as the mobile phase. Due to the proximity of the impurity to the product under several conditions, it is possible that the bands might not be well resolved using some readout methods such as conventional radio-TLC scanners. When using DMSO as the reaction solvent, [^{18}F]Flumazenil samples exhibited up to 3 bands: [^{18}F]fluoride (R_f = 0), [^{18}F]Flumazenil (R_f = 0.7), and an unknown impurity (R_f = 0.9), but while using DMF, only 2 bands were observed: [^{18}F]fluoride (R_f = 0) and [^{18}F]Flumazenil (R_f = 0.8), with no impurity evident (also not observed using radio-HPLC).

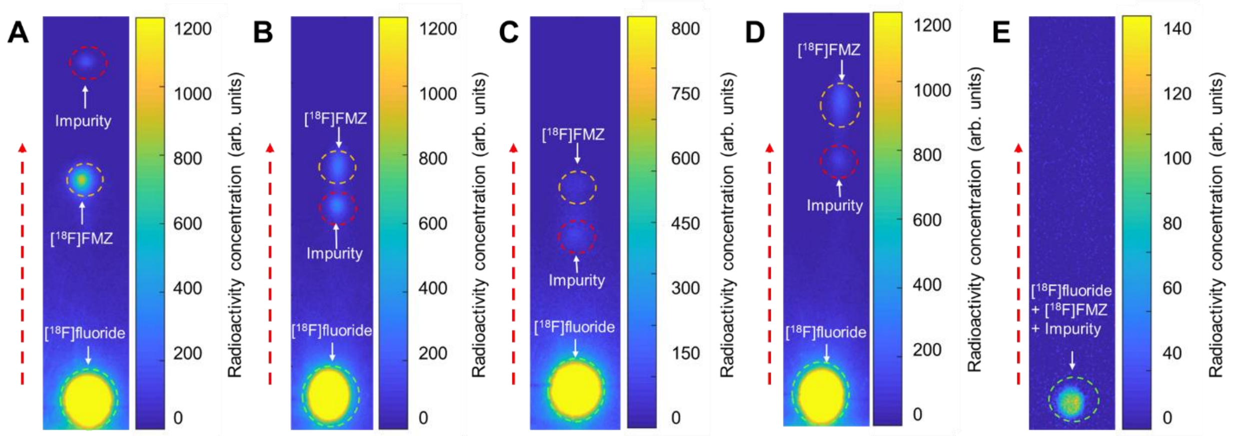

Figure S11. Development of radio-TLC separation method for crude [^{18}F]Flumazenil ([^{18}F]FMZ). (A) Silica gel 60 F_{254} plate with mobile phase of 100% MeCN. (B) Silica gel 60 F_{254} plate with mobile phase of ethyl acetate: ethanol (80:20 v/v) as reported in Ryzhikov *et al.*[1]. (C) Silica gel 60 F_{254} plate with mobile phase ethyl acetate: ethanol: water (80:15:5 v/v) as reported in Vaulina *el al*.[2] and Nasirzadeh *et al*.[3]. (D) RP-18 silica gel 60 F_{254} plate with mobile phase ethyl acetate: ethanol (95:5 v/v) as reported in Mandap *et al.*[4]. (E) RP-18 silica gel 60 F_{254} plate with mobile phase MeCN: water (90:10 v/v). Dashed circles represent the ROIs for analysis. The dashed arrow represents the direction of solvent flow during development.

[^{18}F]PBR06 samples were spotted on silica gel 60 F_{254} plates and separated using 13:10:24:54 (v/v) dichloromethane:chloroform:acetone:hexanes as the mobile phase. Chromatograms exhibited up to 2 bands: [^{18}F]fluoride (R_f = 0) and [^{18}F]PBR06 (R_f = 0.4). [^{18}F]FEPPA samples were spotted on silica gel 60 F_{254} plates and separated using 25.6:37.5:36.5:0.4 (v/v) nBuOH:THF:hexanes:TEA as the mobile phase. Chromatograms exhibited up to 2 bands: [^{18}F]fluoride (R_f = 0) and [^{18}F]FEPPA (R_f = 0.6). **Figure S12** shows example TLC images confirming the separation.

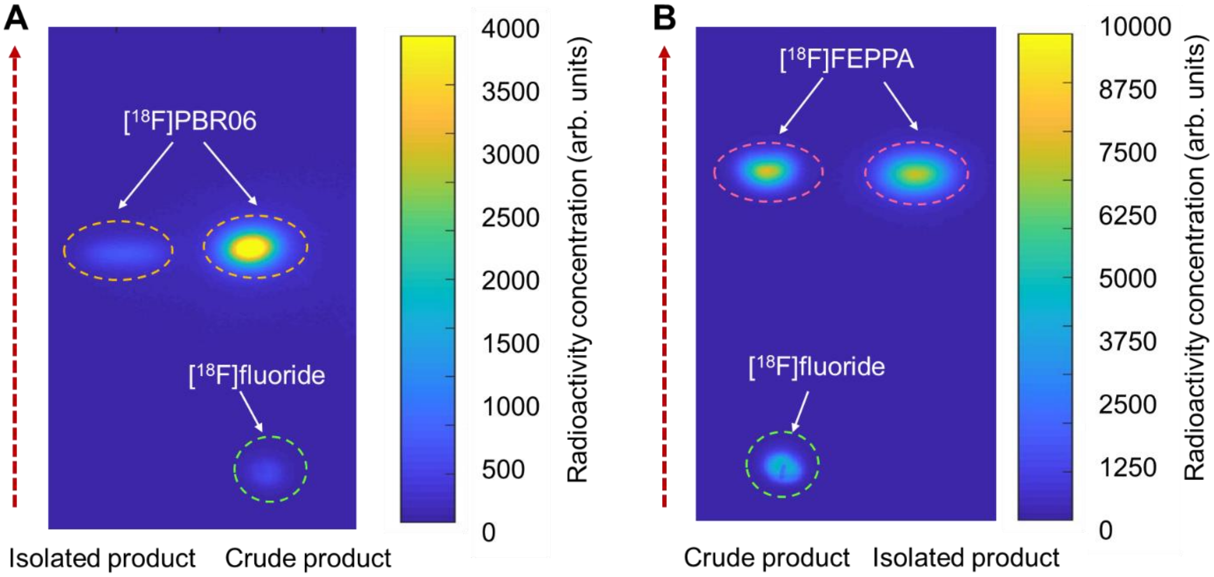

Figure S12. Cerenkov images of TLC plates confirming separation for [^{18}F]PBR06 and [^{18}F]FEPPA. (A) Isolated [^{18}F]PBR06 (left lane) and the crude product after the end of synthesis (right lane). (B) Crude [^{18}F]FEPPA (left lane) and isolated product (right lane). The dashed red arrows indicate the direction of solvent movement during development. (To show more details of bands, images are cropped and do not include the full extent of the TLC plate.)

[^{18}F]Fallypride samples were spotted on silica gel 60 F_{254} plates and separated using 60% MeCN in 25 mM HN_4HCO_2 with 1% TEA (v/v), as previously reported[5]. [^{18}F]Fallypride chromatograms exhibited up to 3 bands: [^{18}F]fluoride (R_f = 0), [^{18}F]Fallypride (R_f = 0.8), and an impurity was previously reported when using high base amounts for the reaction (R_f = 0.9)[5].

5 Optimization of [^{18}F]Flumazenil synthesis

5.1 Reaction temperature and solvent

Experiments were performed by drying of [^{18}F]fluoride/[^{18}O]H_2O under identical conditions (13-15 MBq [0.35-0.40 mCi], mixed with 480 nmol of $TBAHCO_3$), and then performing fluorination at different temperatures and in different solvents. The first batch of experiments was performed with heaters 1 – 4 set at 100, 120, 140, and 160 °C, respectively, and a second batch was performed with heaters 1 – 4 set at 180, 200, 220, and 240 °C, respectively (**Figure 3A**). Other parameters in the reaction were chosen to be similar to other syntheses we have adapted to droplet format, i.e., 8 µL reaction volume, 480 nmol of $TBAHCO_3$, and 280 nmol of precursor[5–7]. The initial reaction time was chosen to be 2 min, matching the condition reported for a flow microreactor[8]. The crude [^{18}F]Flumazenil product was then collected with 40 µL of 2:1 v/v solvent/water mixture (i.e., the same solvent as used in the reaction). The collection solution loading and collecting were 10 µL at a time and were repeated a total of 4 times to minimize the residue left behind at the reaction. Cerenkov images of chips showing residual activity after collection are shown in **Figure 3B** of the main paper and radio-TLC data are shown in **Figure 3C & D.** of the main paper.

Detailed analyses for each individual reaction (collection efficiency, fluorination efficiency, crude RCY, and activity left on-chip) are tabulated in **Table S3**.

Table S3. Summary of data acquired when exploring the effect of temperature and solvent in the radiosyntheses of [^{18}F]Flumazenil.

Solvent	Temperature (°C)	Collection efficiency (%) n=4	Fluorination efficiency (%) n=4	Crude RCY (%) n=4	Activity left on chip (%) n=4
DMSO	100	93.4 ± 1.7	1.1 ± 0.5	1.0 ± 0.5	0.6 ± 0.1
	120	90.6 ± 3.5	4.0 ± 0.4	3.6 ± 0.5	1.1 ± 0.2
	140	78.8 ± 4.4	9.7 ± 0.9	7.7 ± 0.6	1.6 ± 0.1
	160	64.6 ± 2.5	13.3 ± 1.1	8.6 ± 0.7	1.8 ± 0.2
	180	33.4 ± 0.7	26.1 ± 2.0	8.7 ± 0.8	1.7 ± 0.2
	200	25.7 ± 2.5	41.7 ± 3.4	10.7 ± 1.6	2.2 ± 2.0
	220	12.8 ± 2.3	47.1 ± 1.6	5.9 ± 1.1	8.9 ± 2.6
	240	10.3 ± 2.7	50.0 ± 1.6	5.2 ± 1.5	8.9 ± 2.5
DMF	100	93.6 ± 2.4	2.1 ± 0.5	2.0 ± 0.5	1.0 ± 0.2
	120	90.0 ± 0.3	5.1 ± 0.6	4.6 ± 0.6	2.0 ± 0.4
	140	75.8 ± 4.2	14.2 ± 2.8	10.7 ± 1.7	2.3 ± 0.5
	160	65.7 ± 4.3	19.1 ± 1.8	12.5 ± 0.8	2.5 ± 0.2
	180	55.8 ± 3.2	21.7 ± 0.9	12.1 ± 0.8	2.9 ± 0.2
	200	49.0 ± 3.6	27.5 ± 0.9	13.5 ± 0.6	3.1 ± 0.5
	220	25.1 ± 3.3	30.5 ± 2.1	7.7 ± 1.4	5.4 ± 1.0
	240	17.9 ± 2.6*	39.1 ± 4.1*	7.0 ± 1.2*	5.7 ± 1.5 *

*One reaction was performed incorrectly, and so only n=3 repeats are summarized

5.2 Base amount and solvent

Experiments to explore the effect of base amount were conducted by mixing [^{18}F]fluoride/[^{18}O]H_2O (13-15 MBq [0.35-0.40 mCi]) with different amounts of the base $TBAHCO_3$ for the drying step as shown in **Figure S13A**. Chip 1 and 2 explored different base amounts using DMF as the solvent, and chips 3 and 4 explored different base amounts using DMSO as the solvent. All reactions were performed using 280 nmol of precursor in 8 μL of solvent and reacting for 2 min at 200°C. Cerenkov images of chips showing residual activity after collection are shown in **Figure S13B** and radio-TLC data from reactions is shown in **Figure S14**. Detailed analyses for each reaction are tabulated in **Table S4**.

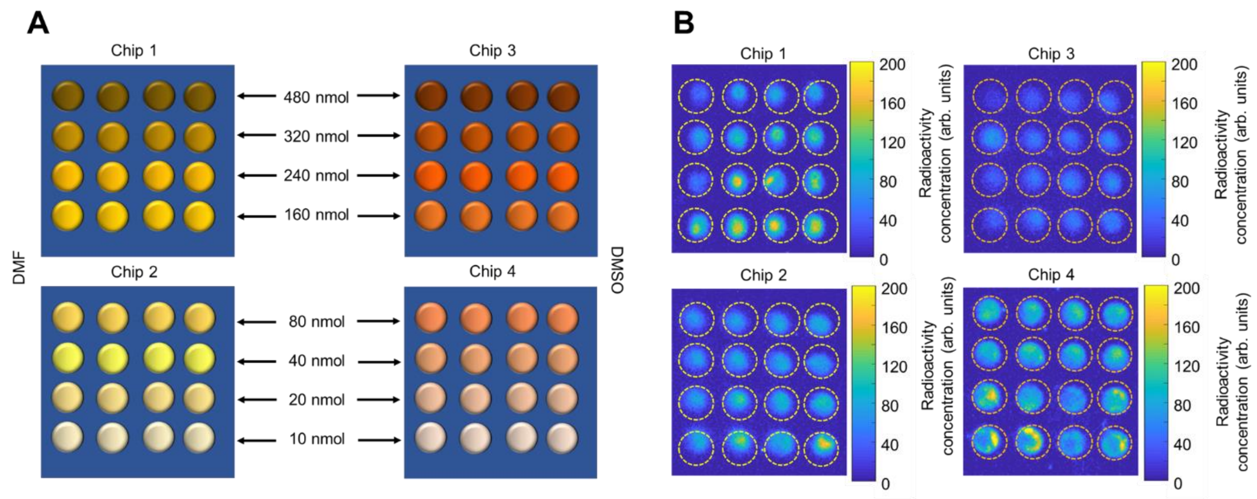

Figure S13. (A) Experimental setup for one batch of experiments that explored the influence of base amount (8 values) and solvent (2 types) on the synthesis of [^{18}F]Flumazenil. All 64 reactions were run simultaneously. (B) Cerenkov images showing the distribution of the residual activity on each chip after the collection of all the crude samples. Brightness is decay-corrected to a common timepoint for all images.

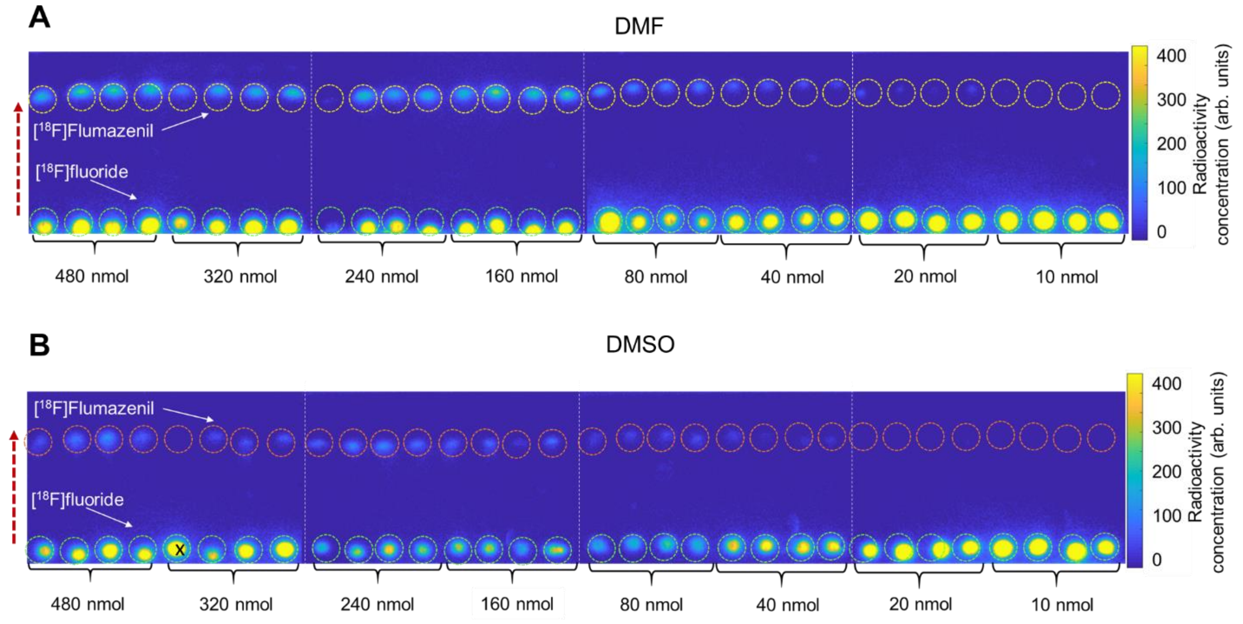

Figure S14. Cerenkov images of TLC plates (each containing 8 samples) after developing. In this case, each TLC plate contains samples from two different base amounts in one reaction solvent (n=4 replicates each). White dotted lines show the edges of each separate multi-sample TLC plate. (A) Spotted crude samples using DMF as the reaction solvent. (B) Spotted crude samples using DMSO as the reaction solvent. Dashed circles indicate the ROIs used for analysis. The dashed red arrow indicates the direction of solvent movement during development. The "X" represents an experimental error and was excluded from further analysis.

Table S4. Summary of data acquired when exploring the effect of the base amount in the radiosyntheses of [^{18}F]Flumazenil in two different solvents.

Solvent	Base amount (nmol)	Collection efficiency (%) n=4	Fluorination efficiency (%) n=4	Crude RCY (%) n=4	Activity left on chip (%) n=4
DMF	480	47.0 ± 5.0	25.8 ± 3.6	12.1 ± 1.6	3.7 ± 1.9
	320	37.7 ± 8.0	30.5 ± 4.5	11.5 ± 0.5	5.4 ± 0.7
	240	38.7 ± 2.2	32.1 ± 2.8	12.4 ± 0.5	6.0 ± 2.0
	160	39.0 ± 3.9	32.7 ± 2.8	12.7 ± 0.9	7.9 ± 1.0
	80	27.7 ± 2.5	16.9 ± 3.1	4.6 ± 0.4	4.6 ± 0.2
	40	27.6 ± 2.5	7.0 ± 1.4	1.9 ± 0.5	4.9 ± 0.4
	20	37.6 ± 3.3	4.3 ± 0.7	1.6 ± 0.1	5.7 ± 0.7
	10	43.8 ± 3.0	1.1 ± 0.4	0.5 ± 0.1	6.8 ± 1.0
DMSO	480	43.0 ± 2.0	22.5 ± 2.5	9.7 ± 1.2	3.9 ± 1.0
	320	30.1 ± 0.5	32.5 ± 2.3	9.8 ± 0.3	5.2 ± 0.3
	240	27.9 ± 6.5	32.2 ± 1.8	9.0 ± 2.0	5.3 ± 0.8
	160	21.8 ± 2.7	22.9 ± 3.8	5.0 ± 1.2	5.2 ± 0.6
	80	25.6 ± 1.5	21.0 ± 2.0	5.4 ± 0.5	5.1 ± 0.3
	40	32.7 ± 2.2	8.2 ± 2.2	2.7 ± 0.6	5.3 ± 0.4
	20	45.6 ± 8.4	3.8 ± 1.5	1.6 ± 0.4	5.9 ± 1.3
	10	55.3 ± 4.8	1.9 ± 0.5	1.0 ± 0.2	6.9 ± 1.5

*One reaction was performed incorrectly, and so only n=3 repeats are summarized

5.3 Precursor amount and solvent

Effect of precursor amount experiments were conducted as depicted in **Figure S15A**. Drying of [^{18}F]fluoride/[^{18}O]H_2O was performed under identical conditions (13-15 MBq [0.35-0.4 mCi], mixed with 240 nmol of $TBAHCO_3$), and the subsequent fluorination reactions were performed with different amounts of precursor dissolved in the 8 µL droplet and reacted at 200°C for 2 min. Chips 1 and 2 used DMF as the solvent, while chips 3 and 4 used DMSO. Cerenkov images of chips showing residual activity after collection are shown in **Figure S15B** and radio-TLC data is shown in **Figure S16**. Detailed analyses for each individual reaction are tabulated in **Table S5**.

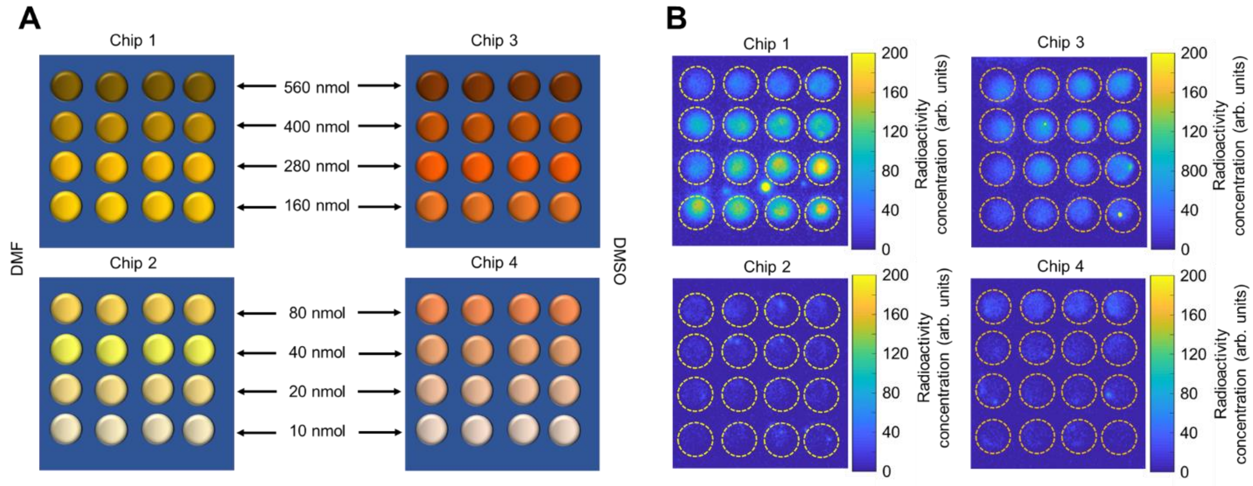

Figure S15. (A) Experimental setup for one batch of experiments that explored the influence of precursor amount (8 values) and solvent (2 types) on the synthesis of [^{18}F]Flumazenil. All 64 reactions were performed simultaneously. (B) Cerenkov images show the distribution of the residual activity on each chip after collecting all the crude samples. Brightness is decay-corrected to a common timepoint for all images.

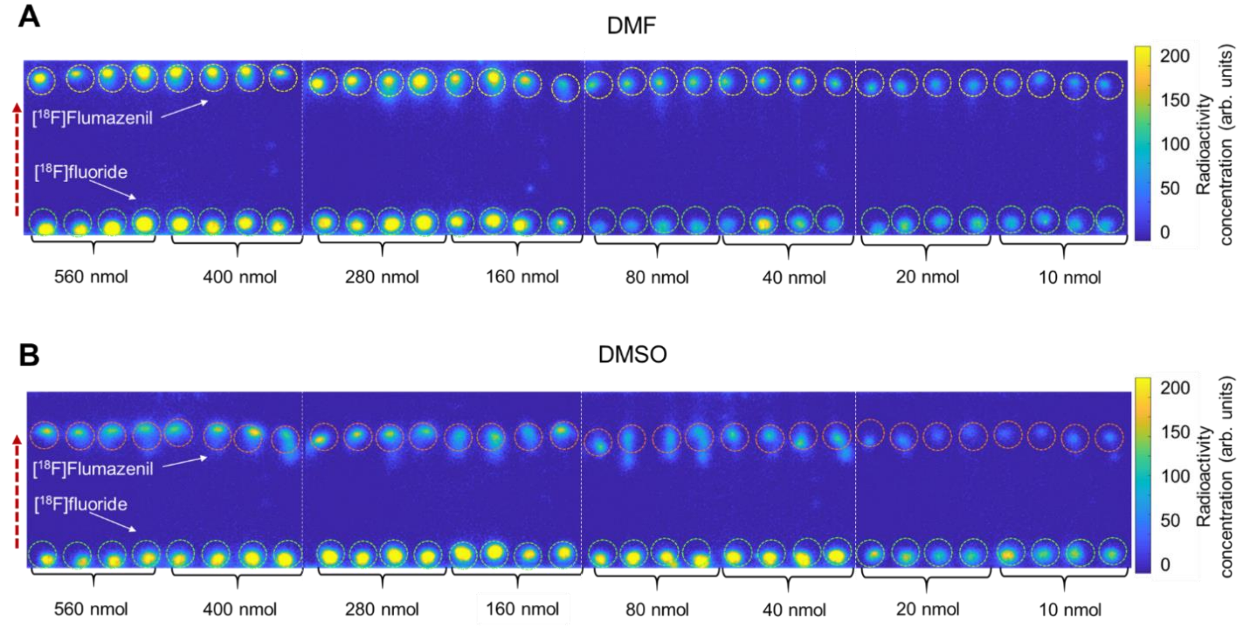

Figure S16. Cerenkov images of TLC plates (each containing 8 samples) after developing in the mobile phase. In this case, each TLC plate contains samples from two different precursor amount conditions in one reaction solvent (n=4 replicates each). White dotted lines show the edges of each separate multi-sample TLC plate. (A) Spotted crude samples using DMF as the reaction solvent. (B) Spotted crude samples using DMSO as the reaction solvent. Dashed circles indicate the ROIs used for analysis. The dashed red arrow indicates the direction of solvent movement during development.

Table S5. Summary of data acquired when exploring the effect of precursor amount in the radiosyntheses of [^{18}F]Flumazenil.

Solvent	Precursor amount (nmol)	Collection efficiency (%) n=4	Fluorination efficiency (%) n=4	Crude RCY (%) n=4	Activity left on chip (%) n=4
DMF	560	23.3 ± 6.8	39.2 ± 4.9	9.2 ± 3.1	2.7 ± 0.5
	400	21.0 ± 4.2	44.6 ± 4.6	9.5 ± 2.7	5.3 ± 0.5
	280	24.0 ± 3.3	47.9 ± 1.6	11.5 ± 1.4	6.1 ± 1.9
	160	19.6 ± 3.5	48.3 ± 5.3	9.5 ± 2.3	6.6 ± 0.6
	80	20.4 ± 0.5	54.5 ± 2.3	11.1 ± 0.3	5.9 ± 0.3
	40	10.0 ± 1.8	48.0 ± 9.0	4.7 ± 0.3	4.9 ± 0.7
	20	8.7 ± 0.9	41.6 ± 3.7	3.4 ± 0.2	4.3 ± 0.3
	10	7.3 ± 0.7	41.9 ± 4.0	3.1 ± 0.3	3.7 ± 1.0
DMSO	560	22.3 ± 1.9	43.9 ± 4.0	9.7 ± 0.4	5.5 ± 0.6
	400	24.9 ± 1.7	39.8 ± 3.0	9.9 ± 0.2	6.3 ± 0.8
	280	23.9 ± 2.8	35.4 ± 3.6	8.5 ± 1.6	5.6 ± 1.6
	160	21.6 ± 3.3	38.3 ± 4.8	8.2 ± 1.3	3.9 ± 0.7
	80	20.7 ± 2.3	36.8 ± 2.3	7.6 ± 1.0	9.1 ± 0.6
	40	17.3 ± 1.2	32.9 ± 1.8	5.7 ± 0.5	4.2 ± 0.8
	20	14.2 ± 1.2	27.7 ± 3.2	3.9 ± 0.7	3.6 ± 1.0
	10	13.0 ± 0.8	19.7 ± 3.3	2.5 ± 0.3	2.7 ± 0.7

5.4 Effect of base to precursor ratio

Figure S17 summarizes the effect of base to precursor ratio on collection efficiency, fluorination efficiency, and crude RCY. This data has all been previously presented above, but here it is reorganized to show the dependence on base to precursor ratio. **Table S6** tabulates the values of base to precursor ratio used as the x-axis in **Figure S17**.

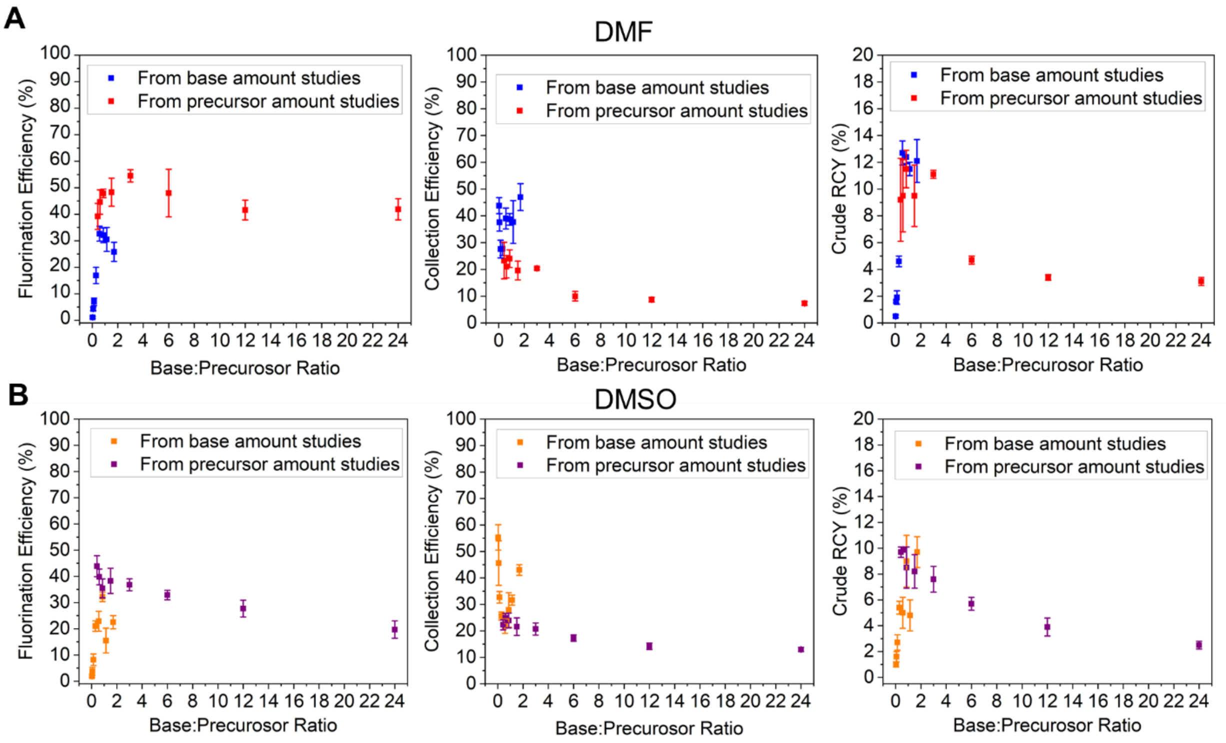

Figure S17. Summary of the impact of the base to precursor molar ratio on the synthesis of [^{18}F]Flumazenil. Data points are derived from other experiments as indicated. Graphs in (A) used DMF as a reaction solvent. Graphs in (B) used DMSO as the reaction solvent.

Table S6. Tabulated values of the base to precursor ratios used in both DMF and DMSO.

	**Base:Precursor (nmol:nmol)**	**Base:Precursor ratio**		**Base:Precursor (nmol:nmol)**	**Base:Precursor Ratio**
**Data from base amount study**	480:280	1.71	**Data from precursor amount study**	240:560	0.43
	320:280	1.14		240:400	0.60
	240:280	0.86		240:280	0.86
	160:280	0.57		240:160	1.50
	80:280	0.29		240:80	3.00
	40:280	0.14		240:40	6.00
	20:280	0.07		240:20	12.0
	10:280	0.04		240:10	24.0

5.5 Reaction time and solvent

The study of the effect of reaction time on the synthesis of [^{18}F]Flumazenil was conducted as shown in **Figure S18A**. First, [^{18}F]fluoride/[^{18}O]H_2O was dried under identical conditions (13-15 MBq [0.35-0.40 mCi], mixed with 240 nmol of $TBAHCO_3$), and then the fluorinations were carried out for different amounts of time and in different solvents. All fluorinations used 280 nmol of precursor in 8 μL of reaction solvent and were carried out at 200 °C. A first batch of experiments used reaction times of 30, 60, 120, and 180 s on heaters 1 – 4, respectively, followed by a second batch with reaction times of 240, 300, 360, and 420 s. Cerenkov images of chips showing residual activity after collection are shown in **Figure S18B** and radio-TLC data are shown in **Figure S19.** Detailed analyses for each individual reaction are tabulated in **Table S7**.

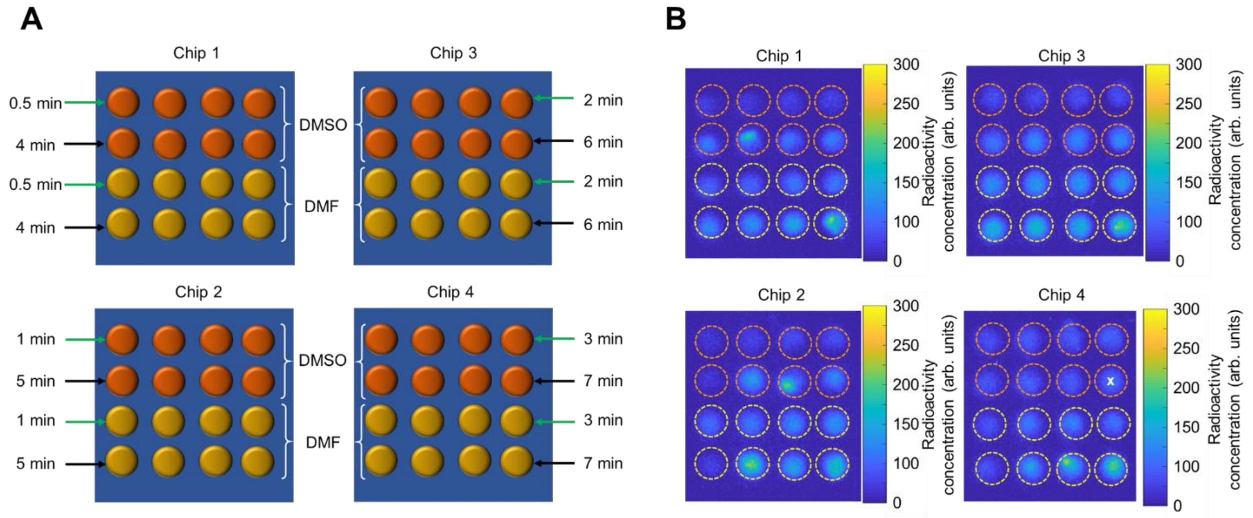

Figure S18 (A) Experimental setup for one batch of experiments that explored the influence of reaction time (8 values) and solvent (2 types) on the synthesis of [^{18}F]Flumazenil. (B) Cerenkov images showing the distribution of the residual activity on each chip after collection of all the crude samples. Brightness is decay-corrected to a common timepoint for all images. The reaction marked with an “X” was not analyzed as a mistake was made in the reaction.

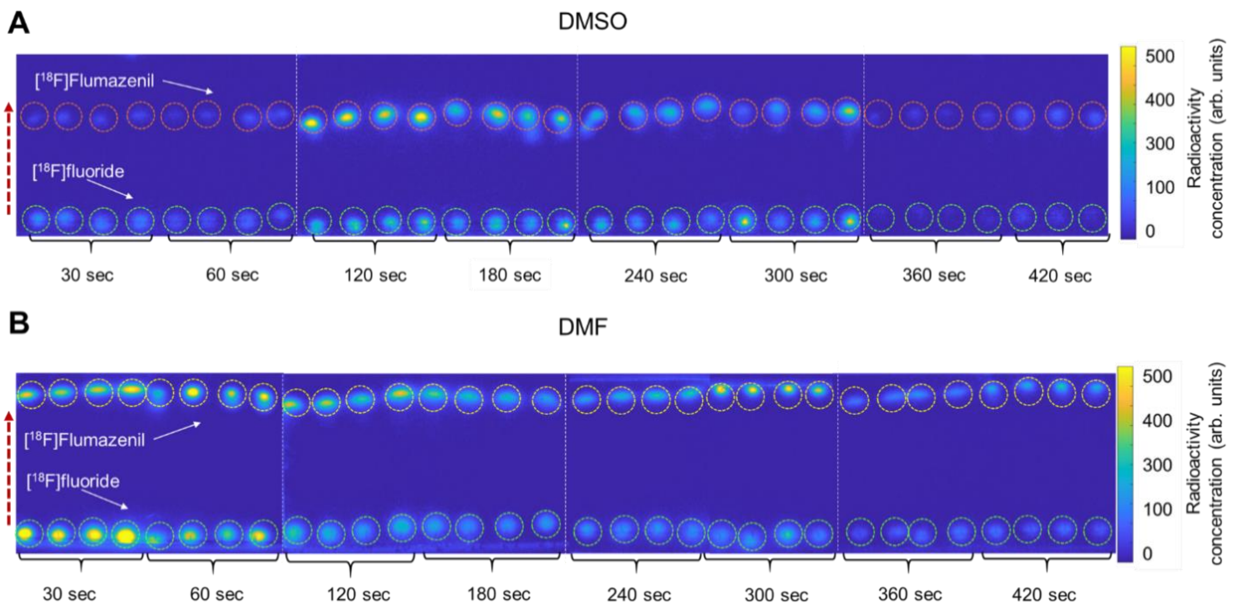

Figure S19: Cerenkov images of TLC plates (each containing 8 samples) after developing in the mobile phase. In this case, each TLC plate contains samples from two different time conditions in one reaction solvent (n=4 replicates each). White dotted lines represent the boundary of each multi-sample plate. (A) Spotted crude samples using DMSO as the reaction solvent. (B) Spotted crude samples using DMF as the reaction solvent. Dashed circles indicate the ROIs used for analysis. The dashed red arrow indicates the direction of solvent movement during development.

Table S7: Summary of data acquired when exploring the effect of reaction time and solvent in the radiosyntheses of [^{18}F]Flumazenil.

**Solvent**	**Time (min)**	**Collection efficiency (%) n=4**	**Fluorination efficiency (%) n=4**	**Crude RCY (%) n=4**	**Activity left on chip (%) n=4**
**DMSO**	0.5	27.1 ± 0.7	26.7 ± 2.7	7.2 ± 0.8	2.5 ± 0.1
	1.0	20.2 ± 0.6	36.4 ± 8.8	7.4 ± 1.8	2.8 ± 0.6
	2.0	14.2 ± 1.9	44.1 ± 4.3	6.2 ± 0.7	4.5 ± 0.4
	3.0	15.8 ± 1.0	65.0 ± 3.4	10.3 ± 0.7	4.5 ± 0.4
	4.0	14.9 ± 1.2	63.4 ± 3.9	9.4 ± 0.8	5.1 ± 0.9
	5.0	12.7 ± 4.4	60.1 ± 4.2	7.6 ± 2.2	7.7 ± 2.9
	6.0	12.5 ±0.8	60.8 ± 4.5	7.5 ± 1.0	5.2 ± 0.6
	7.0	16.8 ± 0.4*	66.7 ± 4.9*	11.2 ± 0.6*	7.2 ± 0.9*
**DMF**	0.5	35.7 ± 1.6	43.1 ± 2.0	15.4 ± 0.9	4.9 ± 0.2
	1.0	29.4 ± 4.4	47.5 ± 3.8	14.0 ± 2.4	5.1 ± 0.9
	2.0	26.8 ± 2.2	51.4 ± 1.6	13.7 ± 1.3	6.2 ± 0.5
	3.0	26 ± 2.1	54.4 ± 5.2	14.4 ± 1.5	6.7 ± 1.5
	4.0	18.3 ± 1.7	55.9 ± 4.0	10.6 ± 1.2	5.8 ± 0.2
	5.0	17.7 ± 0.3	59.8 ± 2.8	10.6 ± 0.4	6.0 ± 0.2
	6.0	18.8 ± 1.3	57.8 ± 2.1	11.0 ± 0.5	5.5 ± 0.3
	7.0	16.3 ± 0.8	61.2 ± 4.4	10.0 ±0.8	6.4 ± 0.4

*Reaction was performed incorrectly and so only n=3 repeats are summarized

5.6 Further studies of reaction solvent and temperature

We further explored the use of different aprotic solvents with a high boiling point for reactions at 200°C. In addition to DMF and DMSO, the solvents tested were N-methyl-2-pyrrolidone (NMP; BP: 202 °C), 1,3-dimethyl-3,4,5,6-tetrahydro-2(1H)-pyrimidinone (DMPU; BP: 247 °C), and ethylene glycol (BP: 197 °C). The experimental design is described in **Figure S20A.** Experiments were performed by drying of [^{18}F]fluoride/[^{18}O]H_2O under identical conditions (13-15 MBq [0.35-0.40 mCi], mixed with 240 nmol of $TBAHCO_3$), and then performing fluorination in different solvents (each replicated n=3 times). Cerenkov image of the chip showing residual activity after collection is shown in **Figure S20B**, and radio-TLC data is shown in **Figure 21.** Detailed analyses for each individual reaction are tabulated in **Table S8**.

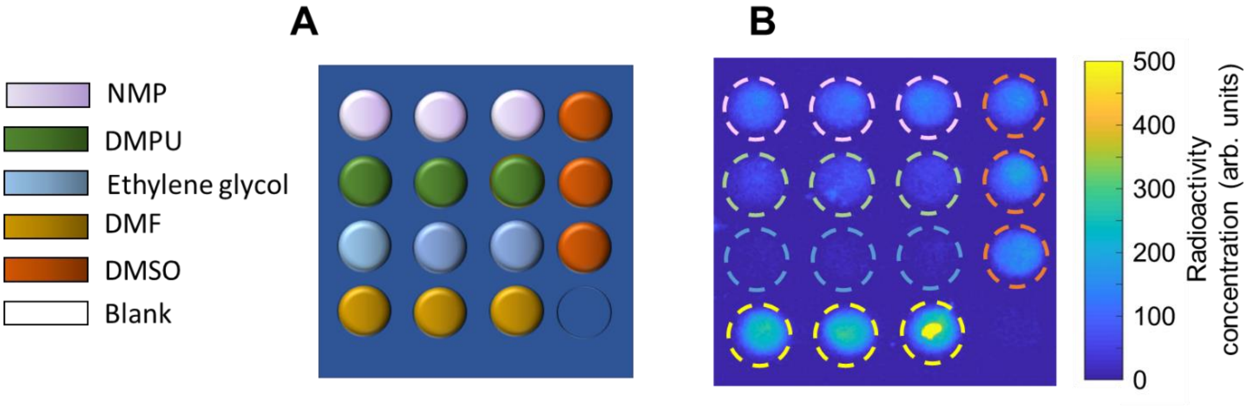

Figure S20. (A) Experimental setup for one batch of experiments that explored the influence of type of solvent (5 types) on the synthesis of [^{18}F]Flumazenil. (B) Cerenkov images showing the distribution of the residual activity on each chip after collection of all of the crude samples.

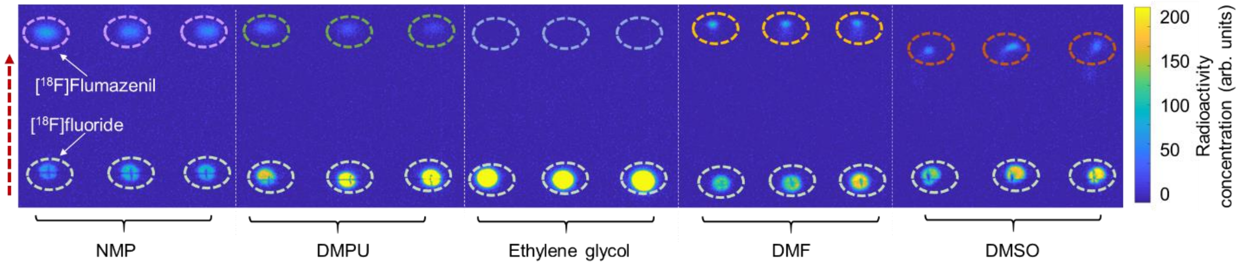

Figure S21: Cerenkov images of TLC plates (each containing 3 samples) after developing in the mobile phase. In this case, each TLC plate contains samples using one of the reaction solvents (n=3 replicates each). Dashed circles indicate the ROIs used for analysis. The dashed red arrow indicates the direction of solvent movement during development.

Table S8. Summary of data acquired when exploring the effect of type of base and reaction solvent in the radiosyntheses of [^{18}F]Flumazenil.

Solvent	Collection efficiency (%) n=3	Fluorination efficiency (%) n=3	Crude RCY (%) n=3	Activity left on chip (%) n=3
**NMP**	37.4 ± 1.1	48.8 ± 6.5	18.2 ± 2.0	5.0 ± 0.3
**DMPU**	49.5 ± 8.2	18.1 ± 3.5	8.8 ± 0.3	3.1 ± 0.8
**Ethylene glycol**	108.5 ± 5.0	2.3 ± 0.5	2.5 ± 0.4	0.6 ± 0.1
**DMF**	46.1 ± 1.8	32.8 ± 2.3	15.1 ± 0.5	10.5 ± 2.9
**DMSO**	35.1 ± 4.5	25.3 ± 0.2	8.9 ± 1.1	5.7 ± 0.6

We then explored the effect of temperature using NMP as reaction solvent for the radiosynthesis of [^{18}F]Flumazenil, as shown in **Figure S22A.** Experiments were performed by drying of [^{18}F]fluoride/[^{18}O]H_2O under identical conditions (13-15 MBq [0.35-0.40 mCi], mixed with 240 nmol of $TBAHCO_3$), and then performing fluorination at different temperatures. Though this experiment could be implemented using 4 chips on 4 heaters, since only 4 reaction sites were needed per temperature value, the experiment was

instead performed using just 2 chips in multiple batches. The first batch of experiments was performed with heaters 1 and 2 set at 100 and 120 °C, respectively, a second batch with temperatures of 140, 160 °C, the third batch with temperatures of 180 and 200 °C, and a final batch with temperatures of 220 and 240 °C. All reactions were performed in 8 µL volume, with 240 nmol base, 280 nmol precursor, and 0.5 min reaction time. Cerenkov images of chips showing residual activity after collection are shown in **Figure S22B**, and radio-TLC data are shown in **Figure S23**. Detailed analyses for each individual reaction are tabulated in **Table S9**, and the results are plotted in **Figure S24**. The optimal temperature was 200 °C, giving a crude RCY of 19.1 ± 0.6% (n=4).

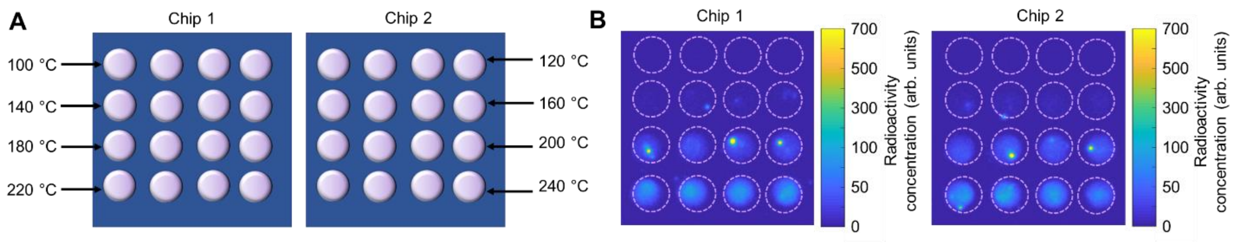

Figure S22. (A) Experimental setup for one batch of experiments that explored the influence of reaction temperature (8 values) in NMP as reaction solvent on the synthesis of [^{18}F]Flumazenil. (B) Cerenkov images showing the distribution of the residual activity on each chip after collection of all the crude samples. Brightness is decay-corrected to a common timepoint for all images.

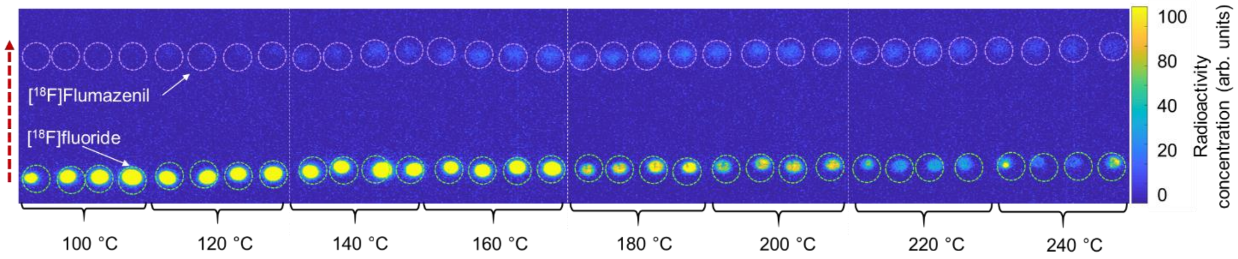

Figure S23. Cerenkov images of TLC plates (each containing 8 samples) after developing in the mobile phase. In this case, each TLC plate contains samples from two different temperatures (n=4 replicates each). White dotted lines show the edges of each separate multi-sample TLC plate. Dashed circles indicate the ROIs used for analysis. The dashed red arrow indicates the direction of solvent movement during development.

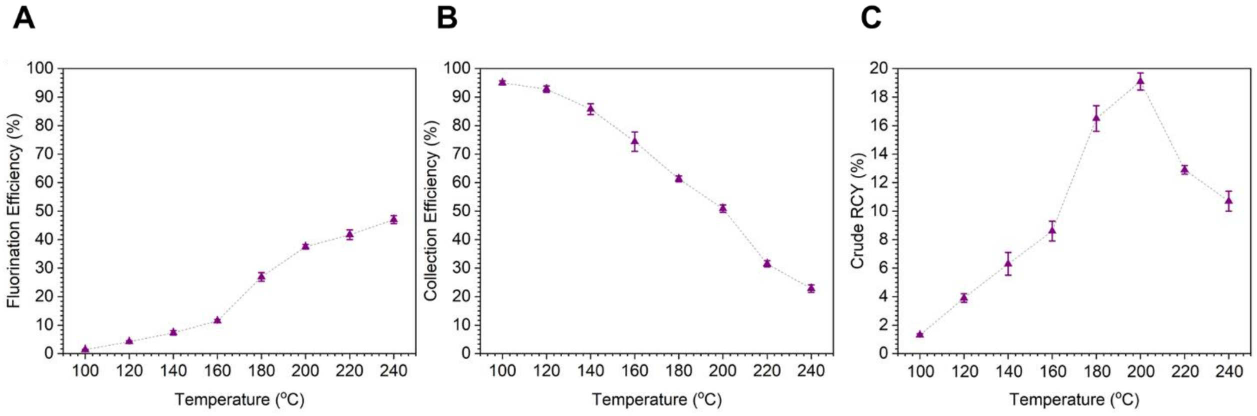

Figure S24. Effect of temperature on the performance of [^{18}F]Flumazenil synthesis with NMP as the reaction solvent. (A) Effect on fluorination efficiency. (B) Effect on collection efficiency. (C) Effect on crude RCY.

Table S9. Summary of data acquired when exploring the effect of temperature (with NMP as reaction solvent) on the radiosyntheses of [^{18}F]Flumazenil.

Temperature (°C)	Collection efficiency (%) n=4	Fluorination efficiency (%) n=4	Crude RCY (%) n=4	Activity left on chip (%) n=4
**100**	95.0 ± 0.7	1.4 ± 0.1	1.3 ± 0.1	0.11 ± 0.01
**120**	92.8 ± 1.1	4.2 ± 0.3	3.9 ± 0.3	0.24 ± 0.03
**140**	85.8 ± 1.9	7.3 ± 0.8	6.3 ± 0.8	0.9 ± 0.2
**160**	74.4 ± 3.4	11.3 ± 0.5	8.6 ± 0.7	1.4 ± 0.4
**180**	61.3 ± 1.0	26.9 ± 1.5	16.5 ± 0.9	7.4 ± 0.9
**200**	50.9 ± 1.3	37.5 ± 0.8	19.1 ± 0.6	8.1 ± 1.7
**220**	31.5 ± 1.1	41.7 ± 1.7	12.9 ± 0.3	12.7 ± 0.1
**240**	22.8 ± 1.3	47.0 ± 1.9	10.7 ± 0.7	11.1 ± 2.1

5.7 Base type and solvent

Finally, we explored the use of different types of base/phase transfer catalyst, comparing Kryptofix (K_{222}) with K_2CO_3, K_{222} with Cs_2CO_3, and $TBAHCO_3$, as well as three different solvents (DMF, DMSO, and NMP). Only 2 chips were used for this study. For the drying step, 13-15 MBq [0.35-0.40 mCi] of [^{18}F]fluoride/[^{18}O]H_2O was loaded to each reaction site. The experiment was organized as shown in **Figure S25A**.–After drying, the subsequent fluorinations were performed with 280 nmol of precursor in 8 µL of DMF, DMSO, or NMP and reacted at 200°C for 0.5 min. Cerenkov image of the chip showing residual activity after collection is shown in **Figure S25B**, and radio-TLC data is shown in **Figure S26**. Detailed analyses for each individual reaction are tabulated in **Table S10**.

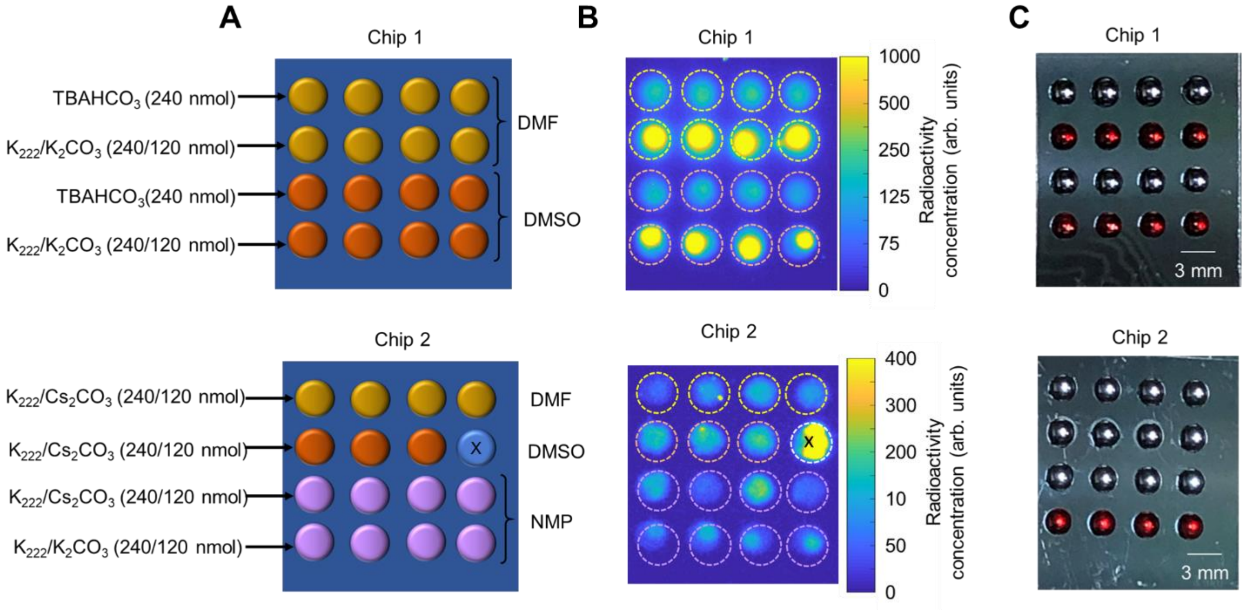

Figure S25. (A) Experimental setup for one batch of experiments that explored the influence of type of base (3 types) and solvent (3 types) on the synthesis of [^{18}F]Flumazenil. (B) Cerenkov images showing the distribution of the residual activity on each chip after the collection of all of the crude samples. (C) Photograph of chips showing the different droplet colors (during fluorination) depending on which base was initially dried on the chip. The reaction marked with an “X” was not analyzed as a mistake was made in the reaction.

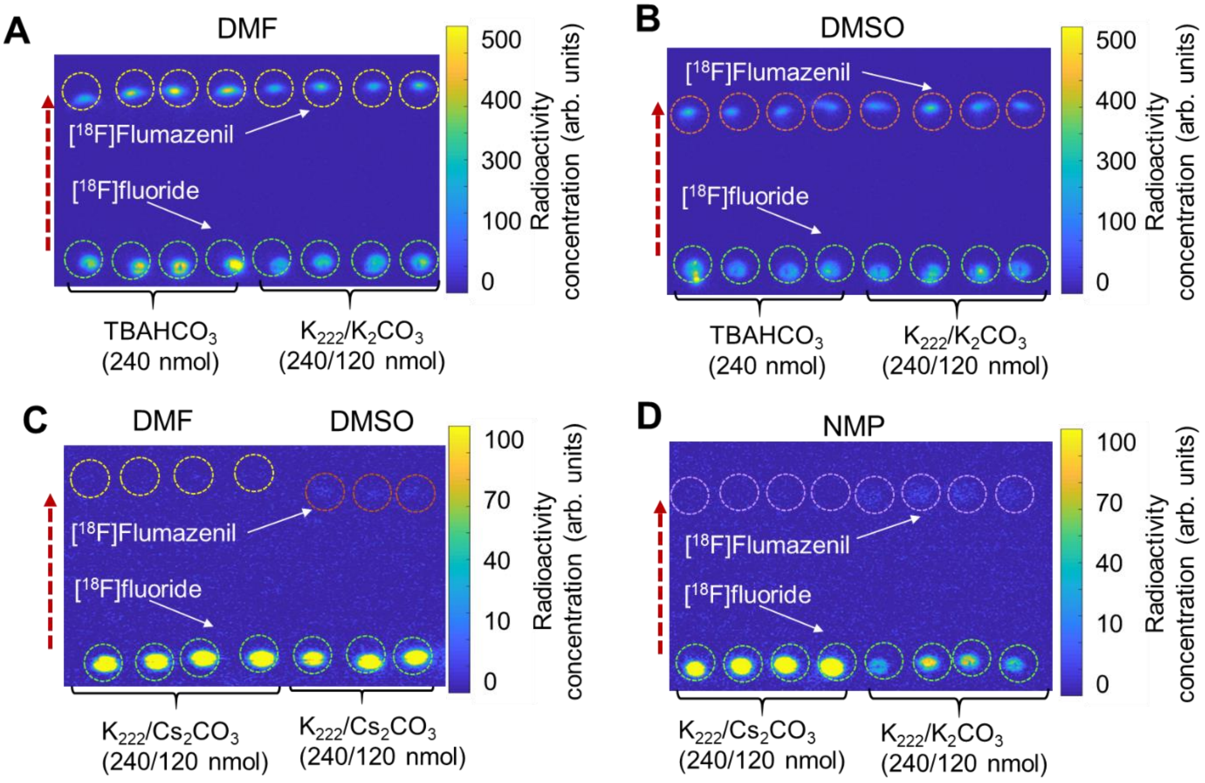

Figure S26: Cerenkov images of TLC plates after developing. Each TLC plate contains data from 2 different combinations of solvent and base/phase transfer catalyst (n=4 replicates each). Dashed circles indicate the ROIs used for analysis. The dashed red arrow indicates the direction of solvent movement during development. (A) Separated crude samples using DMF as the reaction solvent. (B) Separated crude samples using DMSO as the reaction solvent. (C) Separated crude samples using DMF or DMSO as the reaction solvent. (D) Separated crude samples using NMP as the reaction solvent.

Table S10. Summary of data acquired when exploring the effect of type of base and reaction solvent in the radiosyntheses of [^{18}F]Flumazenil.

Solvent	Base type and amount (nmol)	Collection efficiency (%) n=4	Fluorination efficiency (%) n=4	Crude RCY (%) n=4	Activity left on chip (%) n=4
**DMF**	$TBAHCO_3$ (240)	40.5 ± 3.0	38.6 ± 2.6	15.7 ± 1.5	3.3 ± 0.2
	K_{222}/K_2CO_3 (240/120)	28.4 ± 0.8	33.8 ± 5.1	9.6 ± 1.6	8.9 ± 0.9
	K_{222}/Cs_2CO_3 (240/120)	68.1 ± 2.9	2.5 ± 0.6	1.7 ± 0.3	3.7 ± 1.1
**DMSO**	$TBAHCO_3$ (240)	30.8 ± 3.8	33.4 ± 4.7	10.3 ± 2.0	2.8 ± 0.3
	K_{222}/K_2CO_3 (240/120)	30.8 ± 2.2	34.2 ± 2.5	10.4 ± 1.5	6.6 ± 1.4
	K_{222}/Cs_2CO_3 (240/120)	50.3 ± 4.1*	5.1 ± 2.4*	2.5 ± 1.0*	6.1 ± 0.4*
**NMP**	$TBAHCO_3$ (240)	50.9 ± 1.3#	37.5 ± 0.8#	19.1 ± 0.6#	8.1 ± 1.7#
	K_{222}/K_2CO_3 (240/120)	25.8 ± 2.8	21.9 ± 1.7	5.6 ± 0.2	3.1 ± 0.6
	K_{222}/Cs_2CO_3 (240/120)	76.2 ± 5.4	3.4 ± 1.3	2.6 ± 0.9	4.2 ± 1.9

*One reaction was performed incorrectly, and so only n=3 repeats are summarized

Values obtained from the previous experiments of NMP at 200 °C

5.8 Comparison to literature methods

Table S11. Comparison of optimized droplet conditions with literature reports for conventional and flow chemistry synthesis of [^{18}F]flumazenil.

	This work	Wong et al[8]. (2012)	Vaulina et al[2]. (2018)	Nasirzadeh et al[3]. (2016)	Mandap et al[4]. (2009)	Massaweh et al[9]. (2009)	Ryzhikov et al[1]. (2005)
**Synthesizer type**	Microscale (droplet format)	Microscale (flow format)	Macroscale	Macroscale	Macroscale	Macroscale	Macroscale
**Base type**	$TBAHCO_3$	K_{222} / $KHCO_3$	K_{222} / K_2CO_3	K_{222} / K_2CO_3	K_{222} / K_2CO_3	K_{222} / K_2CO_3	K_{222} / K_2CO_3
**Base amount (nmol)**	240	2850 / 2590#	18900 / 10100	25000 / 12000	12100 / 1800	27700 / 1200	25000 / 12000
**Precursor amount (nmol)**	280	1500	4500	3000-6100‡	6100	18000-21000	25000
**Reaction solvent**	NMP	DMF	DMF	DMF	DMF	DMF	DMF
**Reaction volume (mL)**	0.008	0.10	1.5	0.7	0.5-2.0‡	0.6	0.5-1.0
**Temperature (°C)**	200	160	140	150	160	150-160	160
**Reaction time (min)**	0.5	2.5 (residence time)	20	15	5	30	30
**Synthesis time (min)**	35§	N. R.	53	50	55-60	80Δ	75-80Δ
**Starting activity (MBq [mCi])**	13.7 [0.37]	400 [10.8]	10000-2700 [270-730]‡	2000-3000 [54-81]‡	N.R.	50000-56000 [1350-1510]	1800 [49]
**Fluorination efficiency (%)**	42 ± 7 (n=7)	20 (n=1)	23 ± 5 (n=10)	30 ± 7 (n=9)	40 ± 5 (n=7)	27-35 (n=15)	80 (n=1)
**Crude RCY (decay-corrected; %)**	19.1 ± 0.6 (n=4)	–	–	–	–	–	–
**RCY (decay-corrected; %)**	11.6 (n=1)Δ	N.R.	9.0 ± 1.0 (n=6)*	8	26 ± 4	15-20Δ	30Δ

‡ The value corresponding to the optimized condition is not clearly specified, so the whole range reported in the paper is indicated

# Not reported, but amount of $KHCO_3$ was computed based on the amount of precursor and an indicated 1.9:1 molar ratio of base to precursor. The amount of K_{222} was in turn computed based on the reported 1.1:1 molar ratio of K_{222} to $KHCO_3$.

N.R. = Not Reported

* Calculated from shorter and higher-yield SPE purification method instead of HPLC

Δ Isolated yield (i.e., not formulated)

§ 20 min for radiosynthesis and HPLC purification plus an estimated ~15 min additional time for formulation[10]

6 Optimization of [^{18}F]PBR06 synthesis

6.1 Precursor amount and solvent

Effect of precursor amount experiments were conducted as depicted in **Figure S27A**. Drying of [^{18}F]fluoride/[^{18}O]H_2O was performed under identical conditions (13-15 MBq [0.35-0.4 mCi], mixed with 240 nmol of $TBAHCO_3$). The subsequent fluorination reactions were performed with different amounts of precursor dissolved in the 8 μL droplet, and reacted at 100 °C for 5 min. Chips 1 and 2 used thexyl alcohol: MeCN (1:1, v/v) as the solvent while chips 3 and 4 used DMSO. Cerenkov images of chips showing residual activity after collection are shown in **Figure S27B**, and radio-TLC data is shown in **Figure S28**. Detailed analyses for each individual reaction are tabulated in **Table S12**.

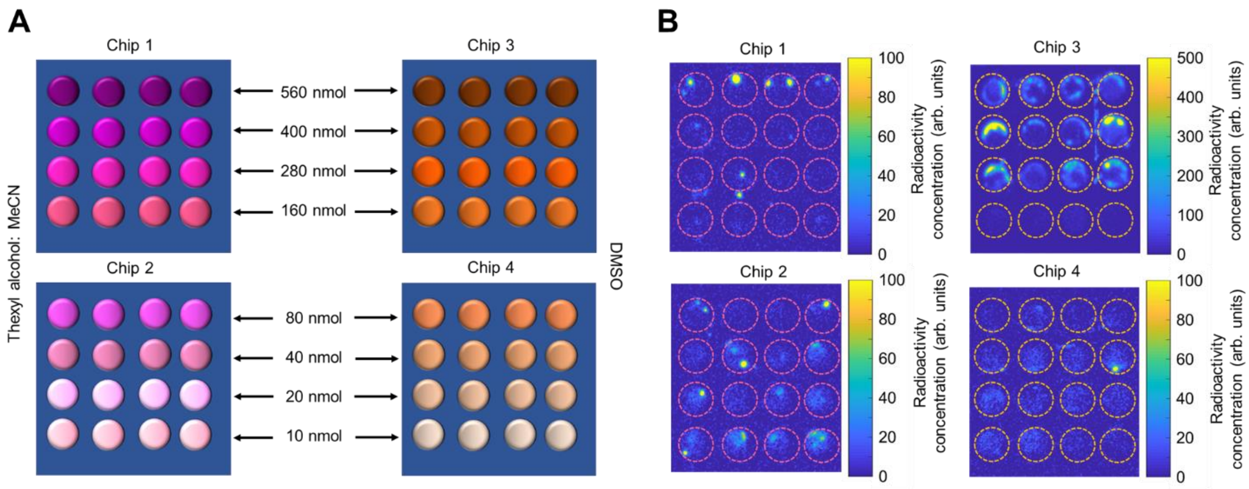

Figure S27. (A) Experimental setup for one batch of experiments that explored the influence of precursor amount (8 values) and solvent (2 types) on the synthesis of [^{18}F]PBR06. All 64 reactions were performed simultaneously. (B) Cerenkov images showing the distribution of the residual activity on each chip after collection of all of the crude samples. Brightness is decay-corrected to a common timepoint for all images.

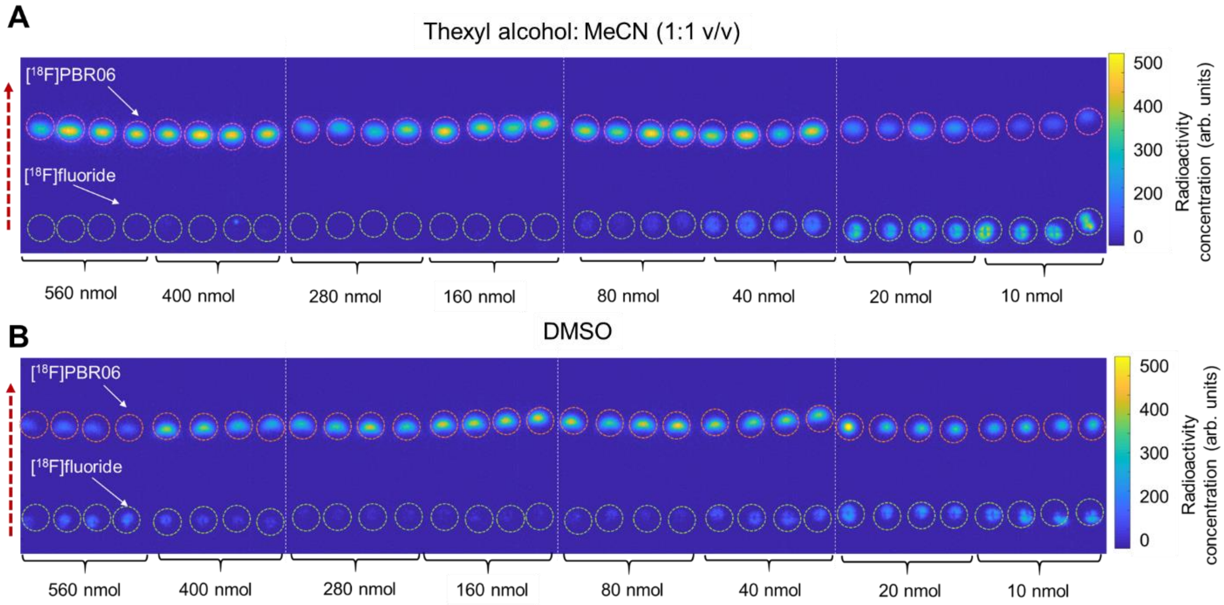

Figure S28. Cerenkov images of TLC plates (each containing 8 samples) after developing in the mobile phase. In this case, each TLC plate contains samples from two different precursor amounts in one reaction solvent (n=4 replicates each). White dotted lines show the edges of each separate multi-sample TLC plate. (A) Image of separated crude samples when using thexyl alcohol:MeCN (1:1 v/v) mixture as the reaction solvent. (B) Image of separated crude samples when using DMSO as the reaction solvent. Dashed circles indicate the ROIs used for analysis. The dashed red arrow indicates the direction of solvent movement during development.

Table S12. Summary of data acquired when exploring the effect of precursor amount in the radiosyntheses of [^{18}F]PBR06.

Solvent	Precursor amount (nmol)	Collection efficiency (%) n=4	Fluorination efficiency (%) n=4	Crude RCY (%) n=4	Activity left on chip (%) n=4
**Thexyl alcohol: MeCN (1:1 v/v)**	560	91.4 ± 1.5	98.0 ± 0.4	91.8 ± 5.2	4.6 ± 1.4
	400	94.9 ± 1.7	96.5 ± 1.5	91.8 ± 3.4	1.2 ± 0.8
	280	91.3 ± 1.6	97.8 ± 0.7	89.3 ± 1.7	2.3 ± 2.1
	160	93.2 ± 4.4	97.8 ± 0.1	91.1 ± 4.3	1.2 ± 0.2
	80	93.1 ± 0.9	92.7 ± 0.8	86.3 ± 1.5	2.4 ± 1.7
	40	94.3 ± 2.9	78.8 ± 1.9	74.4 ± 3.9	2.8 ± 1.2
	20	96.6 ± 2.7	45.4 ± 3.6	43.9 ± 4.7	3.5 ± 1.2
	10	94.6 ± 5.0	24.9 ± 1.6	23.6 ± 2.8	5.0 ± 2.0
**DMSO**	560	48.4 ± 4.2	59.7 ± 6.0	28.8 ± 2.7	4.6 ± 0.7
	400	72.9 ± 7.5	89.2 ± 2.0	65.1 ± 7.5	8.5 ± 6.3
	280	82.5 ± 5.9	93.9 ± 0.4	77.5 ± 5.8	6.7 ± 2.7
	160	90.8 ± 6.1	94.9 ± 0.3	86.3 ± 5.7	0.5 ± 0.2
	80	92.9 ± 0.7	92.8 ± 2.2	86.1 ± 2.2	1.9 ± 1.3
	40	93.5 ± 2.3	81.3 ± 2.1	76.0 ± 0.8	5.3 ± 2.9
	20	91.1 ± 6.4	66.6 ± 0.8	60.7 ± 4.2	4.2 ± 0.8
	10	91.3 ± 3.6	56.5 ± 1.3	51.6 ± 2.5	3.4 ± 1.2

6.2 Base amount and solvent

Investigation of the effect of base amount was conducted by mixing [^{18}F]fluoride/[^{18}O]H_2O (13-15 MBq [0.35-0.40 mCi]) with different amounts of the base $TBAHCO_3$ for the drying step as shown in **Figure S29A**. In the subsequent fluorination, chips 1 and 2 used thexyl alcohol:MeCN (1:1 v/v) mixture as a reaction solvent and chips 3 and 4 used DMSO. All reactions used 160 nmol of precursor and were performed at 100°C for 5 min. Cerenkov images of chips showing residual activity after collection are shown in **Figure S29B**, and radio-TLC data from reactions is shown in **Figure S30**. Detailed analyses for each individual reaction are tabulated in **Table S13**.

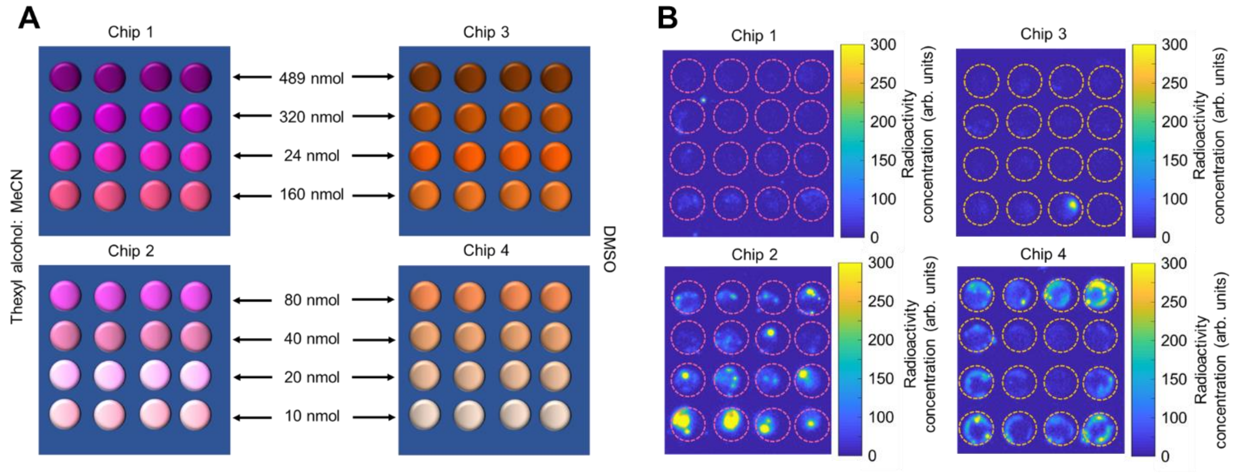

Figure S29. (A) Experimental setup for one set of experiments that explored the influence of base amount (8 values) and solvent (2 types) on the synthesis of [^{18}F]PBR06. All 64 reactions were run simultaneously. (B) Cerenkov images showing the distribution of the residual activity on each chip after collection of all the crude samples. Brightness is decay-corrected to a common timepoint for all images.

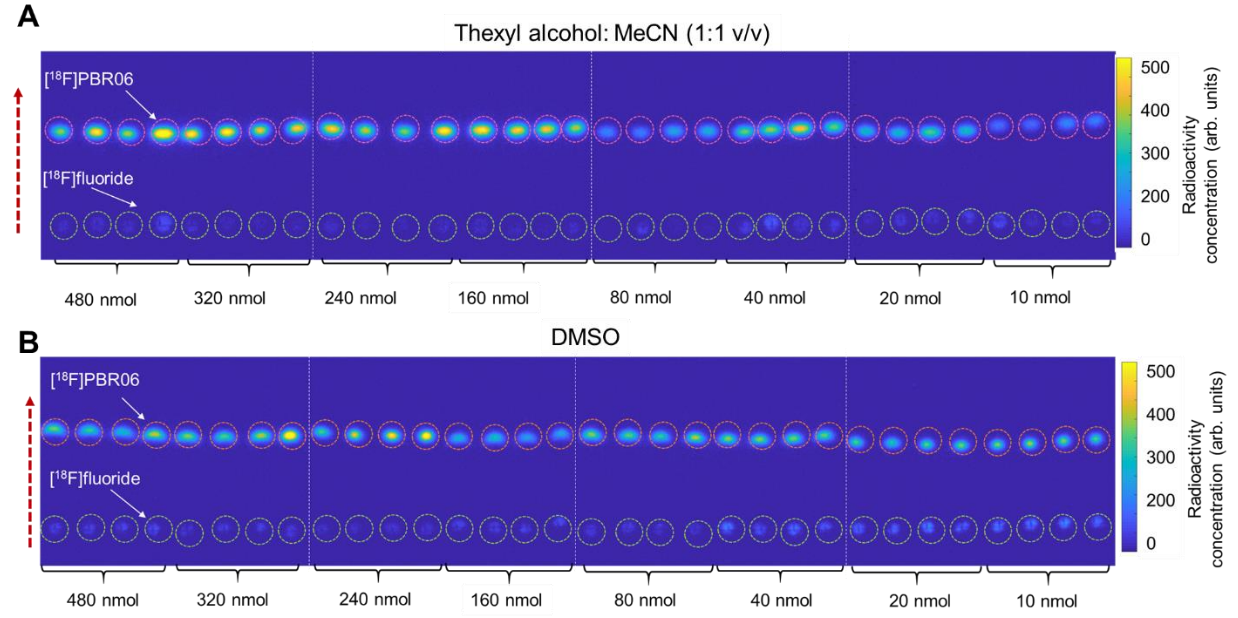

Figure S30. Cerenkov images of TLC plates (each containing 8 samples) after developing in the mobile phase. In this case, each TLC plate contains samples from two different base amount conditions in one reaction solvent (n=4 replicates each). White dotted lines show the edges of each separate multi-sample TLC plate. (A) Image of separated crude samples when using thexyl alcohol:MeCN (1:1 v/v) mixture as the reaction solvent. (B) Image of separated crude samples when using DMSO as the reaction solvent. Dashed circles indicate the ROIs used for analysis. The dashed red arrow indicates the direction of solvent movement during development.

Table S13. Summary of data acquired when exploring the effect of the base amount in the radiosyntheses of [^{18}F]PBR06 in two different solvents.

Solvent	Base amount (nmol)	Collection efficiency (%) n=4	Fluorination efficiency (%) n=4	Crude RCY (%) n=4	Activity left on chip (%) n=4
**Thexyl alcohol:MeCN**	480	94.8 ± 2.4	93.6 ± 2.0	88.7 ± 3.7	1.9 ± 0.7
	320	95.0 ± 1.4	96.8 ± 0.5	91.9 ± 1.4	2.4 ± 2.1
	240	95.5 ± 2.9	95.7 ± 0.6	91.5 ± 3.4	2.0 ± 0.8
	160	92.1 ± 3.9	96.0 ± 1.1	88.5 ± 4.7	5.9 ± 2.0
	80	96.9 ± 3.9	85.3 ± 2.6	98.3 ± 7.5	2.7 ± 1.3
	40	88.2 ± 1.9	89.6 ± 3.9	83.7 ± 4.5	2.1 ± 1.3
	20	88.0 ± 3.7	88.7 ± 3.1	79.4 ± 0.9	4.0 ± 1.6
	10	89.9 ± 6.8	85.2 ± 2.4	75.0 ± 8.5	9.0 ± 4.4
**DMSO**	480	94.4 ± 1.6	89.9 ± 1.5	84.8 ± 1.5	2.7 ± 0.6
	320	94.6 ± 2.4	91.9 ± 2.0	86.9 ± 2.6	2.4 ± 0.6
	240	94.8 ± 4.4	92.6 ± 0.7	87.8 ± 4.2	1.5 ± 0.2
	160	96.8 ± 2.9	85.0 ± 1.7	82.3 ± 4.0	5.0 ± 4.8
	80	86.1 ± 3.4	91.0 ± 0.6	78.4 ± 2.8	7.9 ± 4.5
	40	89.5 ± 1.3	83.2 ± 3.4	74.4 ± 4.1	1.6 ± 0.7
	20	80.7 ± 1.5	77.2 ± 3.8	62.3 ± 2.1	3.0 ± 1.9
	10	74.8 ± 4.0	74.6 ± 1.3	55.8 ± 3.2	5.9 ± 3.0

6.3 Reaction temperature and solvent

The experimental design for exploring temperature and solvent effect on the radiosynthesis of [^{18}F]PBR06 was described in **Figure S31A.** Experiments were performed by drying of [^{18}F]fluoride/[^{18}O]H_2O under identical conditions (13-15 MBq [0.35-0.40 mCi], mixed with 240 nmol of $TBAHCO_3$) , and then performing fluorination at different temperatures and in different solvents. A first batch of experiments was performed with heaters 1 – 4 set at 80, 90, 100, and 110 °C, respectively, and a second batch was performed with heaters 1 – 4 set at 120, 130, 140, and 150 °C, respectively. All fluorinations used 160 nmol of precursor in 8 µL of solvent and were reacted for 5 min. Cerenkov images of chips showing residual activity after collect are shown in **Figure S31B**, and radio-TLC data are shown in **Figure S32**. Detailed analyses for each individual reaction (collection efficiency, fluorination efficiency, crude RCY, and activity left on chip) are tabulated in **Table S14**.

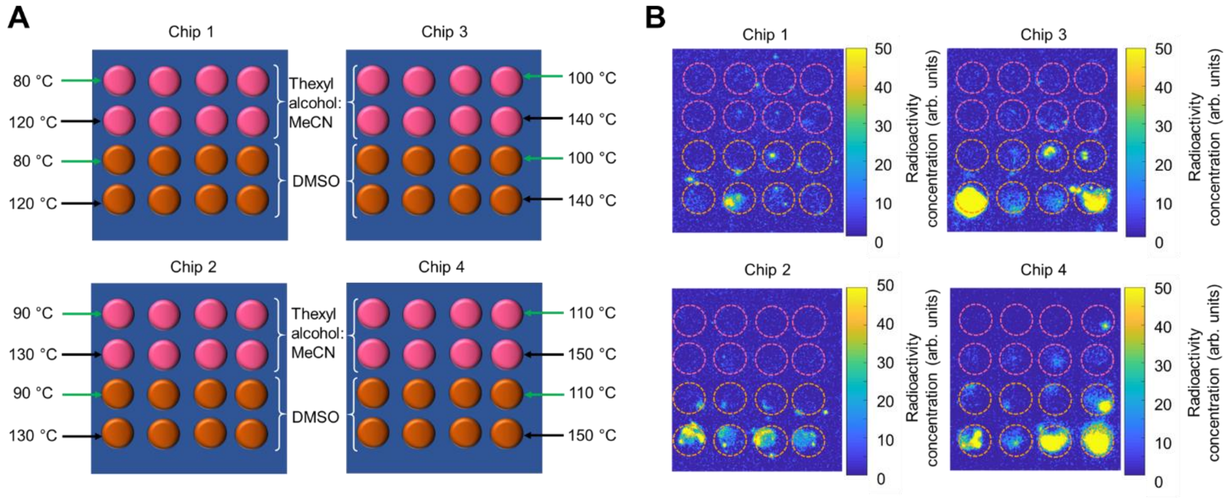

Figure S31. (A) Experimental setup for one batch of experiments that explored the influence of reaction temperature (8 values) and solvent (2 types) on the synthesis of [^{18}F]PBR06. Half of the reaction sites were used first to explore 4 different temperatures in a first set of 32 simultaneous reactions, and then the other half of the sites were used to explore 4 additional temperatures in a second set of 32 simultaneous reactions. (B) Cerenkov images showing the distribution of the residual activity on each chip after collection of all the crude samples. Brightness is decay-corrected to a common timepoint for all images.

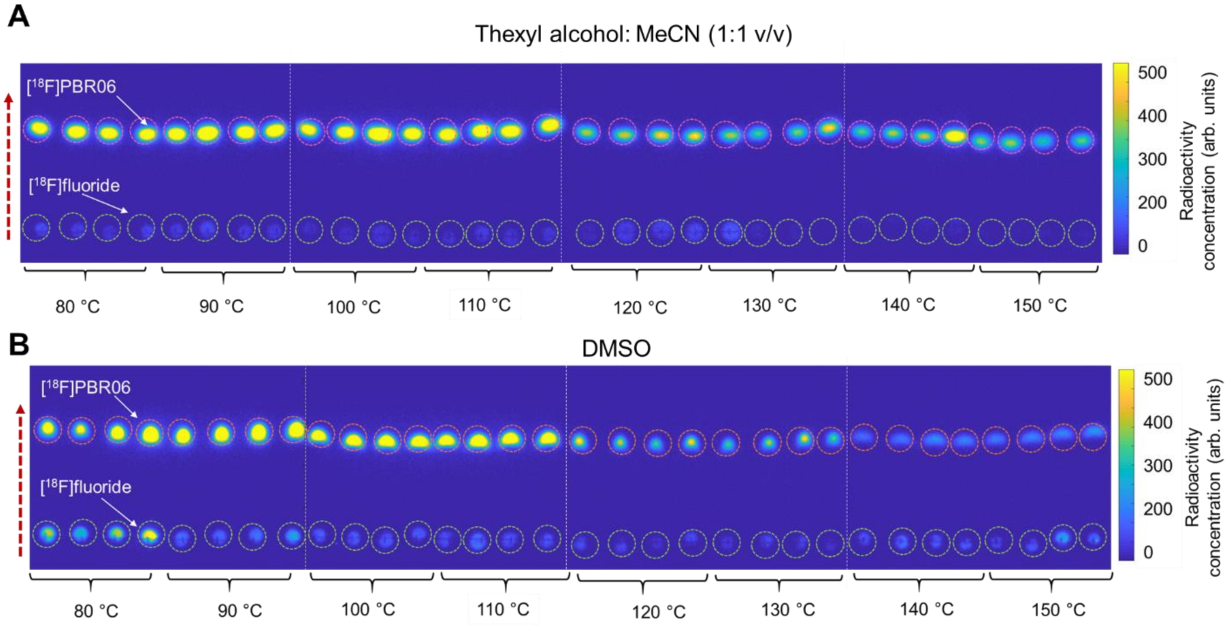

Figure S32. Cerenkov images of TLC plates (each containing 8 samples) after developing in the mobile phase. In this case, each TLC plate contains samples from two different temperature conditions in one reaction solvent (n=4 replicates each). White dotted lines represent the boundary of each multi-sample plate. (A) Image of separated crude samples when using thexyl alcohol:MeCN (1:1 v/v) as the reaction solvent. (B) Image of separated crude samples when using DMSO as the reaction solvent. Dashed circles indicate the ROIs used for analysis. The dashed red arrow indicates the direction of solvent movement during developing.

Table S14. Summary of data acquired when exploring the effect of temperature and solvent in the radiosyntheses of [^{18}F]PBR06.

Solvent	Temperature (°C)	Collection efficiency (%) n=4	Fluorination efficiency (%) n=4	Crude RCY (%) n=4	Activity left on chip (%) n=4
Thexyl alchohol: MeCN	80	93.0 ± 3.0	95.0 ± 1.2	88.4 ± 3.6	1.1 ± 0.4
	90	94.1 ± 1.4	95.7 ± 0.4	90.1 ± 1.6	0.6 ± 0.1
	100	94.2 ± 3.1	95.7 ± 0.6	91.1 ± 2.4	0.4 ± 0.2
	110	94.1 ± 1.2	96.3 ± 0.6	90.6 ± 1.1	0.4 ± 0.5
	120	93.6 ± 0.6	88.2 ± 3.7	82.6 ± 4.0	1.3 ± 0.4
	130	93.8 ± 1.4	90.8 ± 8.3	87.5 ± 10.9	0.5 ± 0.2
	140	91.3 ± 1.4	96.4 ± 0.7	91.7 ± 6.2	0.8 ± 0.3
	150	92.7 ± 0.9	94.7 ± 1.1	87.8 ± 1.2	0.9 ± 0.3
DMSO	80	94.3 ± 3.4	72.4 ± 4.4	68.2 ± 4.4	3.7 ± 1.1
	90	93.5 ± 4.8	91.4 ± 2.1	85.6 ± 5.5	2.0 ± 0.4
	100	93.0 ± 2.4	92.7 ± 0.5	86.2 ± 1.9	1.8 ± 1.1
	110	89.5 ± 2.5	91.9 ± 1.8	82.3 ± 1.9	2.5 ± 2.8
	120	89.1 ± 1.7	87.6 ± 1.0	78.7 ± 1.3	5.0 ± 4.1
	130	93.1 ± 3.3	87.6 ± 3.3	81.6 ± 3.5	9.6 ± 2.4
	140	90.6 ± 2.6	71.3 ± 5.9	64.9 ± 5.9	8.5 ± 6.4
	150	88.6 ± 2.4	75.3 ± 4.2	66.8 ± 4.9	8.4 ± 5.0

6.4 Reaction time and solvent

The study of the effect of reaction time on the synthesis of [^{18}F]PBR06 was conducted as shown in **Figure S33A**. First, [^{18}F]fluoride/[^{18}O]H_2O was dried under identical conditions (13-15 MBq [0.35-0.40 mCi], mixed with 240 nmol of $TBAHCO_3$), and then the fluorinations were carried out for different amounts of time and in different solvents. All fluorinations used 160 nmol of precursor in 8 µL of reaction solvent of thexyl alcohol:MeCN (1:1 v/v) mixture or DMSO and were carried out at 100 °C. A first batch of experiments used reaction times of 30, 60, 120, and 180 s on heaters 1 – 4, respectively, followed by a second batch of experiments with reaction times of 240, 300, 360, and 420 s on heaters 1 – 4, respectively. Cerenkov images of chips showing residual activity after collection are shown in **Figure S32B** and radio-TLC data are shown in **Figure S34.** Detailed analyses for each individual reaction are tabulated in **Table S15**.

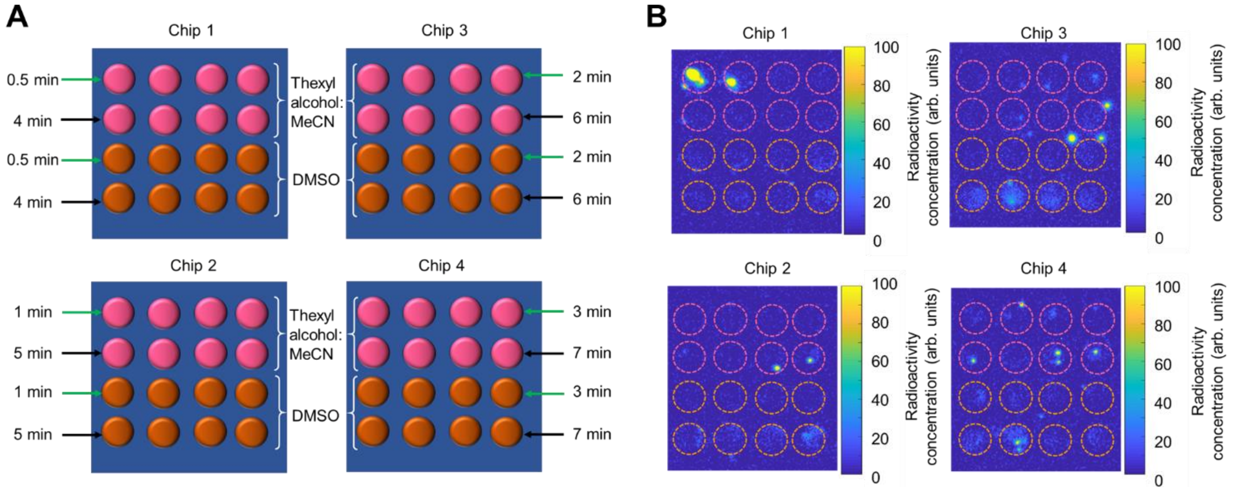

Figure S33. (A) Experimental setup for one batch of experiments that explored the influence of reaction time (8 values) and solvent (2 types) on the synthesis of [^{18}F]PBR06. Half of the reaction sites were used first to explore 4 different times in 32 simultaneous reactions, and then the other half of the sites were used to explore 4 additional times in 32 simultaneous reactions. (B) Cerenkov images showing the distribution of the residual activity on each chip after collection of all the crude samples. Brightness is decay-corrected to a common timepoint for all images.

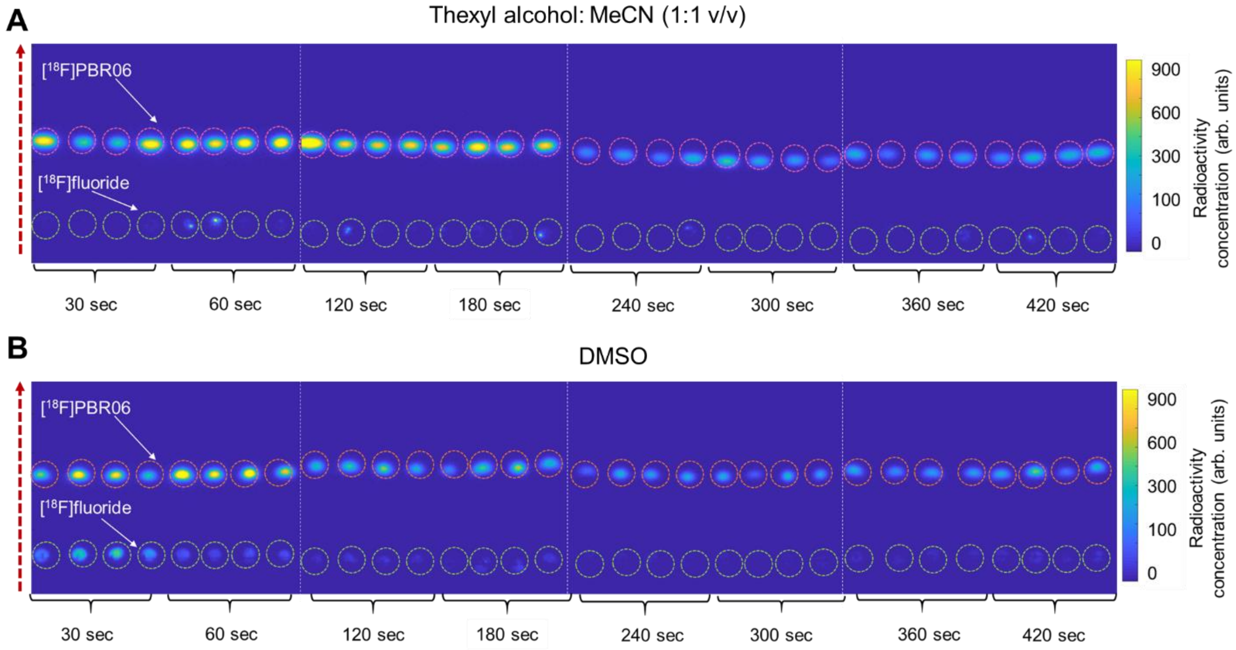

Figure S34: Cerenkov images of TLC plates (each containing 8 samples) after developing in the mobile phase. In this case, each TLC plate contains samples from two different time conditions in one reaction solvent (n=4 replicates each). White dotted lines represent the boundary of each multi-sample plate. (A) Image of separated crude samples when using thexyl alcohol:MeCN (1:1 v/v) mixture as the reaction solvent. (B) Image of separated crude samples using DMSO as the reaction solvent. Dashed circles indicate the ROIs used for analysis. The dashed red arrow indicates the direction of solvent movement during

developing. Red dashed circle represents an area of contamination that was inadvertently on the cover plate when imaging the TLC plates.

Table S15: Summary of data acquired when exploring the effect of reaction time and solvent in the radiosyntheses of [^{18}F]PBR06.

Solvent	Time (min)	Collection efficiency (%) n=4	Fluorination efficiency (%) n=4	Crude RCY (%) n=4	Activity left on chip (%) n=4
**Thexyl alcohol: MeCN**	0.5	92.9 ± 1.6	98.0 ± 0.1	91.0 ± 1.5	2.2 ± 2.0
	1.0	94.1 ± 1.4	94.1 ± 4.4	88.6 ± 4.6	0.5 ± 0.1
	2.0	91.5 ± 2.5	96.5 ± 2.7	88.3 ± 2.2	0.9 ± 0.2
	3.0	94.1 ± 0.9	96.1 ± 2.4	90.4 ± 2.1	0.8 ± 0.2
	4.0	88.7 ± 2.7	95.1 ± 2.7	84.3 ±1.7	0.5 ± 0.2
	5.0	93.3 ± 2.0	97.5 ± 1.0	91.0 ± 1.9	2.6 ± 2.1
	6.0	89.0 ± 5.4	92.6 ± 3.5	82.3 ± 9.1	0.8 ± 0.2
	7.0	94.1 ± 1.2	94.7 ± 2.8	89.1 ± 3.4	2.7 ± 1.7
**DMSO**	0.5	94.2 ± 3.0	66.5 ± 3.1	62.6 ± 3.1	0.6 ± 0.3
	1.0	94.5 ± 3.6	90.4 ± 0.6	85.3 ± 3.0	3.7 ± 2.6
	2.0	92.7 ± 2.0	91.9 ± 1.0	85.2 ± 2.5	0.9 ± 0.4
	3.0	93.8 ± 0.6	90.2 ± 0.9	84.6 ± 0.8	3.8 ± 2.0
	4.0	92.0 ± 2.4	92.8 ± 1.2	85.3 ± 2.6	1.7 ± 0.7
	5.0	91.0 ± 4.3	90.8 ± 1.5	82.7 ± 5.2	3.4 ± 1.8
	6.0	90.5 ± 8.9	90.3 ± 0.6	81.8 ± 8.6	1.5 ± 0.2
	7.0	94.2 ± 4.8	89.9 ± 1.1	84.7 ± 3.5	3.7± 3.3

6.5 Base type and solvent

Finally, we explored the use of different type of base / phase transfer catalyst, comparing Kryptofix (K_{222}) and K_2CO_3 versus $TBAHCO_3$ as shown in **Figure S35A**. Only one chip was used for this study. For the drying step, 13-15 MBq [0.35-0.40 mCi] of [^{18}F]fluoride/[^{18}O]H_2O was loaded to each reaction site. For half of the spots, the fluoride was mixed with 240 nmol of TBAHCO3. For the other half of the spots, the fluoride was mixed with 240 nmol of K_{222} and 120 nmol of K_2CO_3. After drying, the subsequent fluorinations were performed with 280 nmol of precursor in 8 µL of either thexyl alcohol:MeCN (1:1 v/v) mixture or DMSO, and reacted at 100°C for 0.5 min. Cerenkov image of the chip showing residual activity after collection are shown in **Figure S35B**, and radio-TLC data is shown in **Figure S36**. Detailed analyses for each individual reaction are tabulated in **Table S16**.

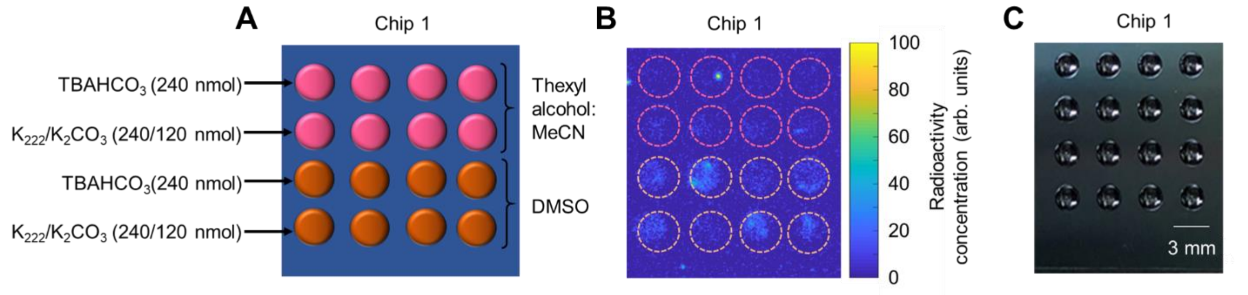

Figure S35. (A) Experimental setup for one batch of experiments that explored the influence of type of base (2 values) and solvent (2 types) on the synthesis of [^{18}F]PBR06. (B) Cerenkov images showing the distribution of the residual activity on each chip after collection of all the crude samples. (C) Photograph of chip after precursor is loaded, showing absence of base-dependent color difference (seen for [^{18}F]Flumazenil).

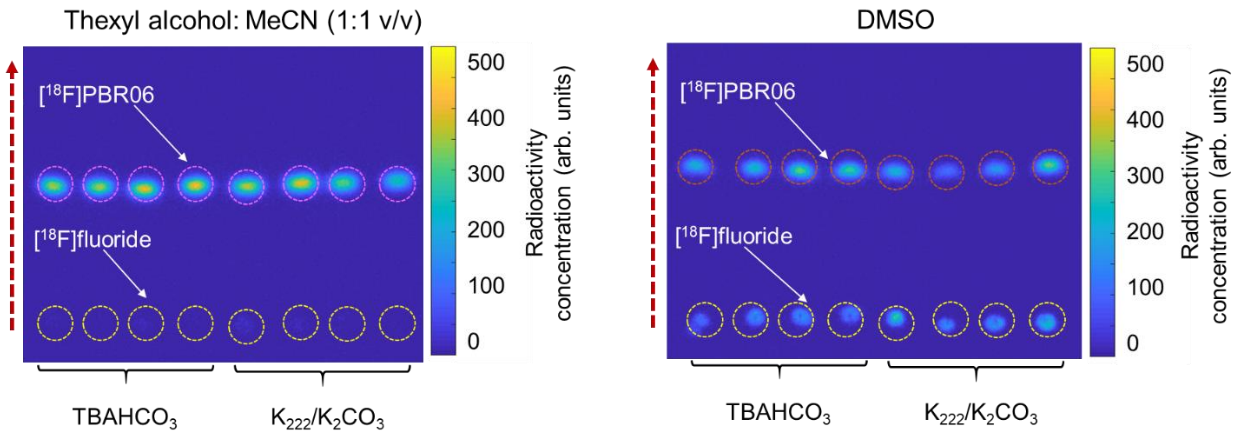

Figure S36: Cerenkov images of TLC plates (each containing 8 samples) after developing in the mobile phase. In this case, each TLC plate contains samples from two different base type conditions in one reaction solvent (n=4 replicates each). Dashed circles indicate the ROIs used for analysis. The dashed red arrow indicates the direction of solvent movement during developing. (A) Image of separated crude samples when using thexyl alcohol:MeCN (1:1 v/v) mixture as the reaction solvent. (B) Image of separated crude samples when using DMSO as the reaction solvent.

Table S16. Summary of data acquired when exploring the effect of type of base and reaction solvent in the radiosyntheses of [^{18}F]flumazenil.

Solvent	Base type and amount (nmol)	Collection efficiency (%) n=4	Fluorination efficiency (%) n=4	Crude RCY (%) n=4	Activity left on chip (%) n=4
**Thexyl alcohol: MeCN**	$TBAHCO_3$ (240)	96.4 ± 2.2	97.4 ± 0.2	93.9 ± 2.0	0.7 ± 0.1
	K_{222}/K_2CO_3 (240/120)	95.8 ± 3.5	96.5 ± 0.9	92.4 ± 3.0	1.63 ± 0.03
**DMSO**	$TBAHCO_3$ (240)	94.7 ± 4.1	70.7 ± 3.5	66.9 ± 2.9	4.4 ± 1.1
	K_{222}/K_2CO_3 (240/120)	93.5 ± 5.3	60.5 ± 3.2	56.5 ± 2.0	4.3± 1.9

6.6 Additional studies of reaction temperature

Using thexyl alcohol: MeCN (1:1 v/v) as a reaction solvent exhibited high crude RCY at 80 °C in a 5 min reaction, and high crude RCY at 100 °C in a 0.5 min reaction. We were interested whether short reactions (0.5 min) at lower temperatures would also give good performance. Experiments were performed by drying of [^{18}F]fluoride/[^{18}O]H_2O under identical conditions (13-15 MBq [0.35-0.40 mCi] with 240 nmol of $TBAHCO_3$), followed by fluorination with 160 nmol of precursor in 8 µL of solvent for 0.5 min at different temperatures. To conserve chips, temperatures were explored sequentially, using 4 fresh reaction sites each time. (Two chips were needed in total.) Radio-TLC data are shown in **Figure S37**. Detailed analyses for each individual reaction are tabulated in **Table S16** and the results are plotted in **Figure S38**. We observed that the temperature could be lowered to 90 °C without compromising performance. **Table S17** compares our approach with other literature macroscale reports.

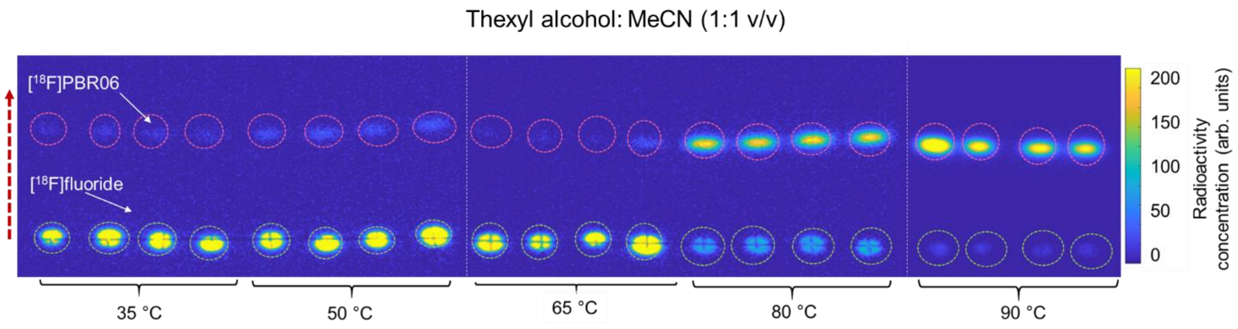

Figure S37. Cerenkov images of TLC plates (two containing 8 samples and one containing 4 samples) after developing in the mobile phase. In this case, each TLC plate contains samples from up to two different temperatures (n=4 replicates each). White dotted lines show the edges of each multi-lane TLC plate. Dashed circles indicate the ROIs used for analysis. The dashed red arrow indicates the direction of solvent movement during developing.

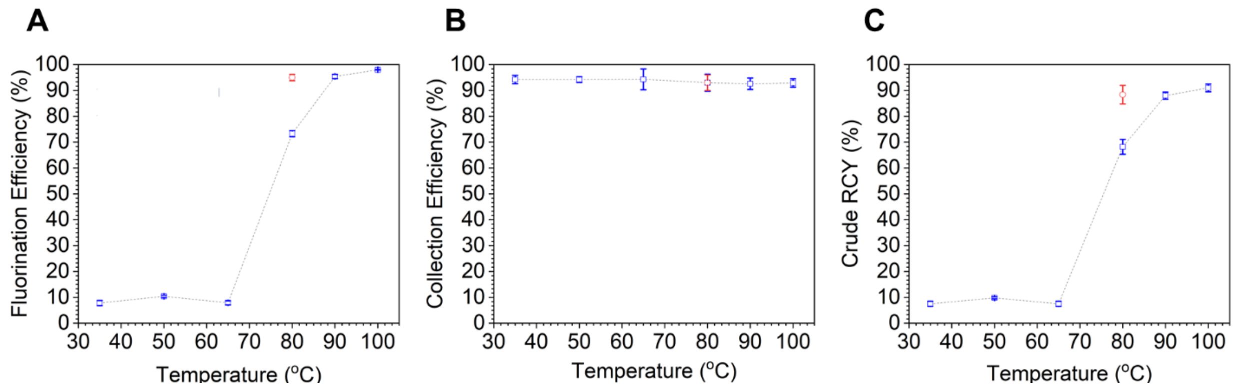

Figure S38. Temperature studies of [^{18}F]PBR06. (A) Fluorination efficiency. (B) Collection efficiency. (C) Crude RCY. The blue markers represent 0.5 min reactions, and the red data points represent 5 min reactions.

Table S17. Summary of data acquired when exploring the effect of temperature in the radiosyntheses of [^{18}F]PBR06 (for 0.5 min reactions).

Solvent	Temperature (°C)	Collection efficiency (%) n=4	Fluorination efficiency (%) n=4	Crude RCY (%) n=4	Activity left on chip (%) n=4
Thexyl alchohol: MeCN	35	94.2 ± 1.6	7.8 ± 1.0	7.5 ± 1.0	2.2 ± 0.2
	50	94.2 ± 1.1	10.4 ± 0.4	9.8 ± 0.4	3.5 ± 1.0
	65	94.3 ± 4.0	7.9 ± 0.8	7.5 ± 1.0	1.5 ± 0.8
	80	93.0 ± 3.3	73.3 ± 1.2	68.2 ± 2.9	1.0 ± 0.6
	90	92.6 ± 2.2	95.4 ± 0.8	88.0 ± 1.4	2.5± 0.7

6.7 Comparison to literature methods

Table S18. Comparison of optimized droplet conditions with literature reports for conventional synthesis of [^{18}F]PBR06.

	This work (2021)	Wang et al.[11] (2011)	Zhang et al.[12] (2019)
**Synthesizer type**	Microscale (droplet format)	Macroscale	Macroscale
**Base type**	$TBAHCO_3$	K_{222} / K_2CO_3	K_{222} / K_2CO_3
**Base amount (nmol)**	240	27000/ 12000	40000/10000
**Precursor amount (nmol)**	160	1800	5500
**Reaction solvent**	Thexyl alcohol: MeCN (1:1 v/v)	DMSO	DMSO
**Reaction volume (mL)**	0.008	1	1
**Temperature (°C)**	100	140	140
**Reaction time (min)**	0.5	15	15
**Synthesis time (min)**	35 #	50-60	50
**Starting activity (MBq [mCi])**	13.8 [0.375]	N.R.	N.R.
**Fluorination efficiency (%)**	98.0 ± 0.1 (n=4)	N.R.	N.R.
**Crude RCY (decay-corrected; %)**	93.9 ± 2.0 (n=4)	N.R.	N.R.
**RCY (decay-corrected; %)**	75.8 (n=1)*	30-60	40-60

* Isolated yield (i.e., not formulated)

20 min for radiosynthesis and purification plus an estimated ~15 min additional time for formulation[10]

N.R. = Not reported

7 Optimization of [^{18}F]Fallypride synthesis

7.1 Precursor concentration and reaction temperature

A set of experiments to explore the effect of precursor concentration and temperatures were conducted as depicted in **Figure S39A**. Drying of [^{18}F]fluoride/[^{18}O]H_2O was performed under identical conditions (13-15 MBq [0.35-0.4 mCi], mixed with 240 nmol of $TBAHCO_3$), and the subsequent fluorination reactions were performed with different amounts of precursor dissolved in the 6 μL droplet, for 7 min. Chip 1 was run at 80 °C, chip 2 at 95 °C chip 3 at 110°C, and chip 4 at 125 °C. All reactions were conducted using thexyl alcohol:MeCN (1:1 v/v) mixture as solvent. Cerenkov images of chips showing residual activity after collected are shown in **Figure S39B**, and graphical representation is shown in **Figure S41A** in the main paper. Detailed analyses for each individual reaction are tabulated in **Table S18**.

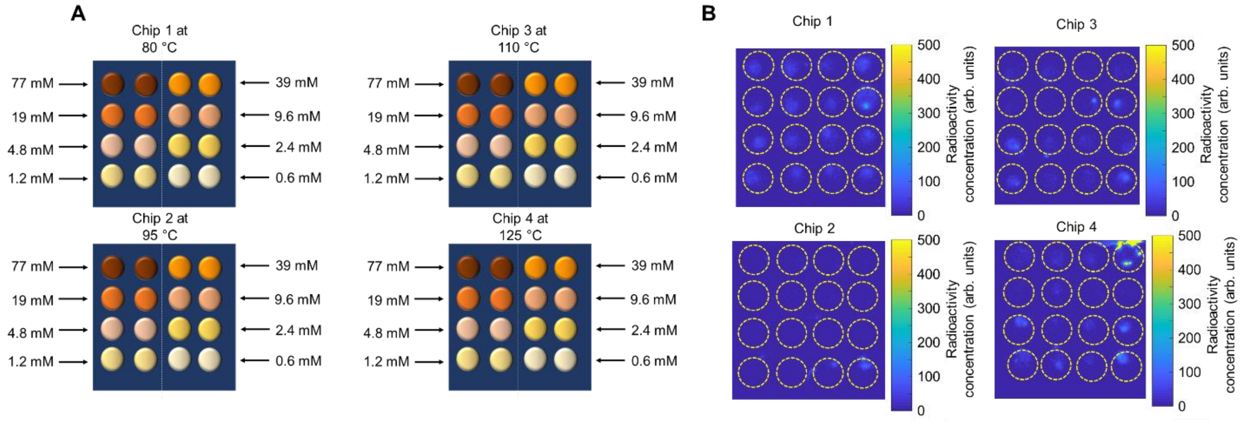

Figure S39. (A) Experimental setup for one batch of experiments that explored the influence of precursor concentration (8 values) and temperature (4 types) on the synthesis of [^{18}F]Fallypride. Each chip explored 8 different precursor concentrations at one particular temperature. (B) Cerenkov images showing the distribution of the residual activity on each chip after collection of all the crude samples. Brightness is decay-corrected to a common timepoint for all images.

Table S19. Summary of data acquired when exploring the effect of precursor concentration and temperature on the radiosyntheses of [^{18}F]Fallypride.

Temperature (°C)	Precursor concentration (mM)	Collection efficiency (%) n=2	Fluorination efficiency (%) n=2	Crude RCY (%) n=2	Activity left on chip (%) n=2
**80**	77	93.64 ± 0.05	94.7 ± 1.3	88.6 ± 1.2	4.1 ± 0.4
	39	93.1 ± 1.6	91.9 ± 4.0	85.7 ± 5.2	4.2 ± 2.4
	19	93.2 ± 0.8	61.7 ± 3.6	57.5 ± 2.9	3.1 ± 1.2
	9.6	91.2 ± 8.3	33.4 ± 3.8	30.6 ± 6.2	5.8 ± 4.4
	4.8	94.9 ± 3.6	24.4 ± 1.7	23.1 ± 0.8	3.4 ± 1.2
	2.4	96.1 ± 2.0	13.0 ± 1.1	12.5 ± 0.7	4.3 ± 0.1
	1.2	93.9 ± 7.4	12.7 ± 0.8	12.0 ± 1.7	3.4 ± 0.7
	0.6	92.5 ± 5.7	6.7 ± 0.7	6.2 ± 0.3	3.8 ± 0.8
**95**	77	88.2 ± 3.0	95.5 ± 0.1	84.3 ± 3.0	4.6 ± 0.7
	39	89.8 ± 3.8	95.1 ± 1.0	85.3 ± 2.7	2.1 ± 1.0
	19	94.4 ± 3.1	85.0 ± 3.6	80.3 ± 6.0	1.4 ± 0.2
	9.6	93.6 ± 4.5	59.7 ± 0.5	55.9 ± 2.2	0.75 ± 0.02
	4.8	92.0 ± 1.2	34.9 ± 2.3	32.1 ± 2.5	1.3 ± 0.2
	2.4	90.3 ± 8.6	19.4 ± 0.4	17.2 ± 2.5	1.0 ± 0.3
	1.2	87.4 ± 9.1	15.9 ± 0.5	13.6 ± 1.4	1.0 ± 0.3
	0.6	89.3 ± 0.8	7.4 ± 0.8	6.6 ± 0.8	8.6 ± 5.5
**110**	77	91.7 ± 2.1	96.1 ± 0.5	88.1 ± 1.5	3.6 ± 0.2
	39	98.7 ± 1.7	96.0 ± 0.2	94.7 ± 1.8	2.1 ± 0.4
	19	94.0 ± 0.8	89.6 ± 0.4	84.2 ± 1.1	1.3 ± 0.4
	9.6	93.5 ± 2.8	61.0 ± 0.4	57.1 ± 2.1	4.6 ± 0.3
	4.8	86.0 ± 3.5	34.0 ± 2.4	29.2 ± 0.8	6.4 ± 3.1
	2.4	88.2 ± 7.4	18.6 ± 0.3	16.3 ± 1.1	3.4 ± 0.5
	1.2	88.6 ± 3.4	15.7 ± 1.1	13.9 ± 0.4	3.9 ± 1.6
	0.6	88.1 ± 6.1	7.1 ± 0.4	6.2 ± 0.1	5.0 ± 3.3
**125**	77	90.6 ± 0.5	95.3 ± 0.6	86.3 ± 1.0	1.34 ± 0.01
	39	90.0 ± 5.6	95.1 ± 0.3	85.5 ± 5.1	3.1 ± 2.8
	19	86.7 ± 3.8	90.2 ± 0.5	78.2 ± 3.0	0.6 ± 0.1
	9.6	85.6 ± 4.3	64.0 ± 3.8	54.7 ± 0.5	0.4 ± 0.1
	4.8	82.8 ± 6.2	37.5 ± 4.0	30.9 ± 0.9	1.7 ± 1.4
	2.4	78.6 ± 4.0	21.8 ± 0.2	17.1 ± 1.0	2.0 ± 1.3
	1.2	85.6 ± 0.7	21.3 ± 2.3	18.2 ± 2.1	2.6 ± 0.4
	0.6	82.6 ± 3.5	7.8 ± 1.5	6.4 ± 1.0	2.6 ± 2.3

7.2 Precursor concentration and reaction time

Another study of the impact of precursor concentration and reaction time was conducted as depicted in **Figure S40A**. Drying of [^{18}F]fluoride/[^{18}O]H_2O was performed under identical conditions (13-15 MBq [0.35-0.4 mCi], mixed with 240 nmol of $TBAHCO_3$), and the subsequent fluorination reactions were performed with different amounts of precursor dissolved in the 6 μL droplet, at 110 °C. All reactions were conducted using thexyl alcohol:MeCN (1:1 v/v) mixture as solvent. Samples on each chip were reacted for different times. Cerenkov images of chips showing residual activity after collection are shown in **Figure S40B**. Detailed analyses for each individual reaction are tabulated in **Table S20**, and the results are plotted in **Figure S41B**.

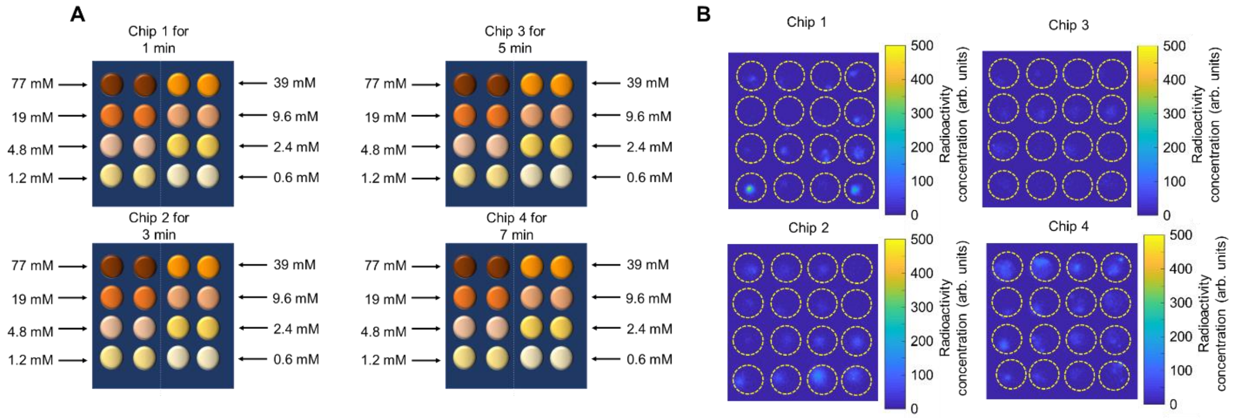

Figure S40. (A) Experimental setup for one set of experiments that explored the influence of reaction precursor amount (8 values) and reaction time (4 types) on the synthesis of [^{18}F]Fallypride. (B) Cerenkov images showing the distribution of the residual activity on each chip after collection of all the crude samples. Brightness is decay-corrected to a common timepoint for all images.

Table S20. Summary of data acquired when exploring the effect of precursor amount and reaction time on the radiosyntheses of [^{18}F]Fallypride.

Time (min)	Precursor concentration (mM)	Collection efficiency (%) n=2	Fluorination efficiency (%) n=2	Crude RCY (%) n=2	Activity left on chip (%) n=2
**1.0**	77	93.4 ± 0.7	80.5 ± 2.8	75.2± 2.1	4.1 ± 1.0
	39	99.2 ± 4.1	93.3 ± 1.1	92.7 ± 5.0	2.8 ± 0.5
	19	92. ± 6.9	93.4 ± 0.1	86.7 ± 6.4	1.7 ± 0.5
	9.6	90.1 ± 0.5	84.3 ± 2.8	75.9 ± 2.1	3.8 ± 1.0
	4.8	92.9 ± 2.3	72.2 ± 0.9	67.0 ± 2.5	3.6 ± 3.2
	2.4	95.9 ± 2.7	31.0 ± 0.6	29.7 ± 1.4	5.7 ± 2.6
	1.2	93.4 ± 4.7	18.4 ± 0.1	17.2 ± 1.3	5.8 ± 5.4
	0.6	95.9 ± 0.4	11.2 ± 1.3	10.7 ± 1.3	5.6 ± 4.9
**3.0**	77	93.8 ± 1.2	90.0 ± 0.1	84.4 ± 1.2	3.7 ± 0.5
	39	94.1 ± 1.5	93.9 ± 0.8	88.4 ± 2.2	1.7 ± 1.0
	19	92.9 ± 1.2	92.8 ± 2.5	86.2 ± 1.2	1.9 ± 0.2
	9.6	91.8 ± 2.7	61.3 ± 1.9	56.3 ± 3.4	1.4 ± 0.8
	4.8	93.4 ± 7.4	31.9 ± 3.3	29.9 ± 5.4	2.3 ± 1.3
	2.4	93.1 ± 4.8	15.4 ± 1.3	14.4 ± 2.0	3.6 ± 0.3
	1.2	92.3 ± 1.1	7.9 ± 1.3	7.3 ± 1.1	4.5 ± 0.3
	0.6	101.7 ± 2.1	5.5 ± 0.2	5.6 ± 0.1	8.7 ± 2.4
**5.0**	77	91.61 ± 0.01	87.0 ± 0.7	79.7 ± 0.7	2.4 ± 0.7
	39	91.0 ± 5.4	89.0 ± 0.2	81.0 ± 5.0	2.4 ± 0.7
	19	94.8 ± 0.4	89.2 ± 1.7	84.5 ± 1.9	3.4 ± 0.7
	9.6	91.6 ± 2.5	63.0 ± 1.5	57.7 ± 3.0	3.7 ± 1.3
	4.8	94.4 ± 7.8	59.2 ± 1.7	55.8 ± 3.0	3.5 ± 2.9
	2.4	93.5 ± 4.6	31.1 ± 5.0	29.2 ± 6.1	2.5 ± 1.2
	1.2	90.8 ± 6.7	16.7 ± 1.6	15.1 ± 0.3	2.1 ± 0.8
	0.6	90.2 ± 8.4	8.2 ± 1.0	7.4 ± 1.6	2.5 ± 1.1
**7.0**	77	89.7 ± 0.5	88.0 ± 1.1	78.9 ± 1.5	8.0 ± 1.0
	39	93.4 ± 2.1	90.7 ± 0.6	84.7 ± 1.3	5.2 ± 0.6
	19	93.5 ± 6.0	88.5 ± 1.2	82.8 ± 6.5	2.1 ± 0.8
	9.6	93.6 ± 3.7	64.8 ± 2.8	61.5 ± 0.2	2.85 ± 0.04
	4.8	93.8 ± 0.8	39.4 ± 1.1	35.9 ± 1.3	3.31 ± 0.03
	2.4	92.8 ± 8.4	17.5 ± 0.4	10.2 ± 1.9	2.9 ± 0.6
	1.2	93.3 ± 3.3	9.0 ± 0.7	8.4 ± 0.9	2.27 ± 0.05
	0.6	91.2 ± 9.9	5.2 ± 0.6	4.8 ± 1.0	2.7 ± 1.3

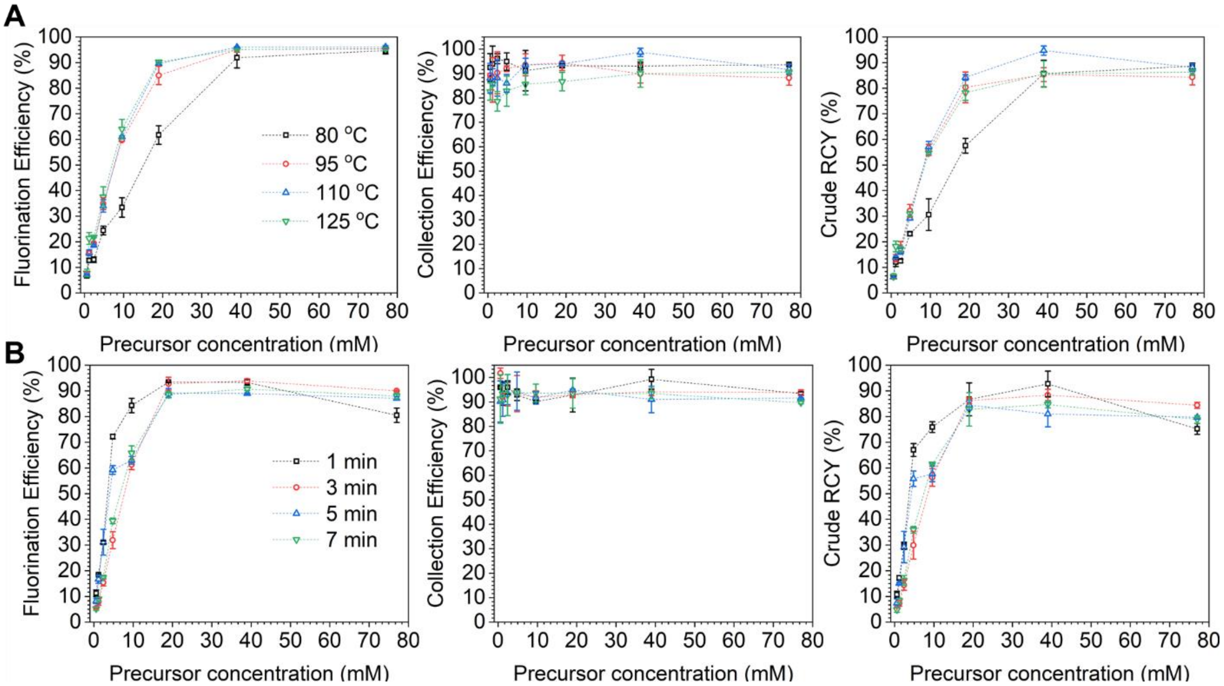

Figure S41. (A) Investigation of [^{18}F]Fallypride synthesis performance reaction performance as a function of reaction temperature and precursor concentration. Reaction volume: 6 µL. Base amount: 240 nmol. Reaction time: 7 min. Reaction solvent: thexyl alcohol and MeCN (1:1 v/v) (B) Investigation of reaction performance as a function of reaction time and precursor concentration. Reaction volume: 6 µL. Base amount: 240 nmol. Temperature: 110°C. Reaction solvent: thexyl alcohol and MeCN (1:1 v/v).

8 Optimization of [^{18}F]FEPPA synthesis

8.1 Reaction temperature

Experiments to explore the effect of reaction temperature were conducted as shown in **Figure S42A**. Drying of [^{18}F]fluoride/[^{18}O]H_2O was performed under identical conditions (13-15 MBq [0.35-0.4 mCi], mixed with 240 nmol of $TBAHCO_3$), and the subsequent fluorination reactions were performed at 8 different temperatures for 2 min using 240 nmol of precursor dissolved in the 8 µL droplet in thexyl alcohol: MeCN (1:1, v/v). Though this experiment could be implemented using 4 chips on 4 heaters, since only 4 reaction sites were needed per temperature value, the experiment was instead performed using just 2 chips as follows. A first batch of experiments was performed with heaters 1 and 2 set at 60 and 100 °C, respectively, a second batch with temperatures of 70, 110 °C, a third batch with temperatures of 80 and 120 °C, and a final batch with temperatures of 90 and 130 °C. Cerenkov images of chips showing residual activity after collection are shown in **Figure S42B**, and radio-TLC data is shown in **Figure S43**. Detailed analyses for each individual reaction are tabulated in **Table S21.**

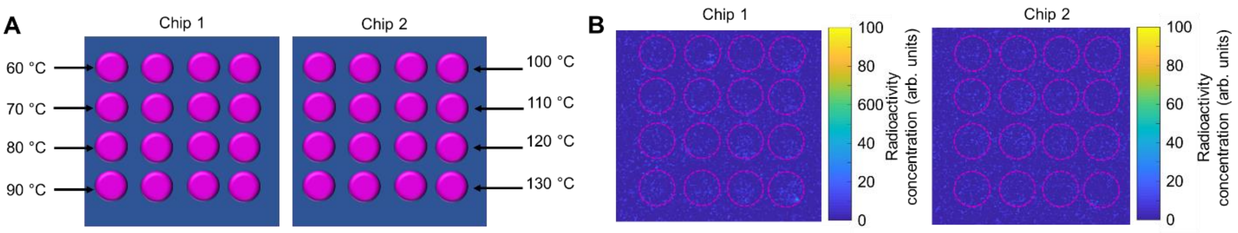

Figure S42. (A) Experimental setup for one batch of experiments that explored the influence of reaction temperature (8 values) on the synthesis of [^{18}F]FEPPA. (B) Cerenkov images showing the distribution of the residual activity on each chip after collection of all the crude samples. Brightness is decay-corrected to a common timepoint for all images.

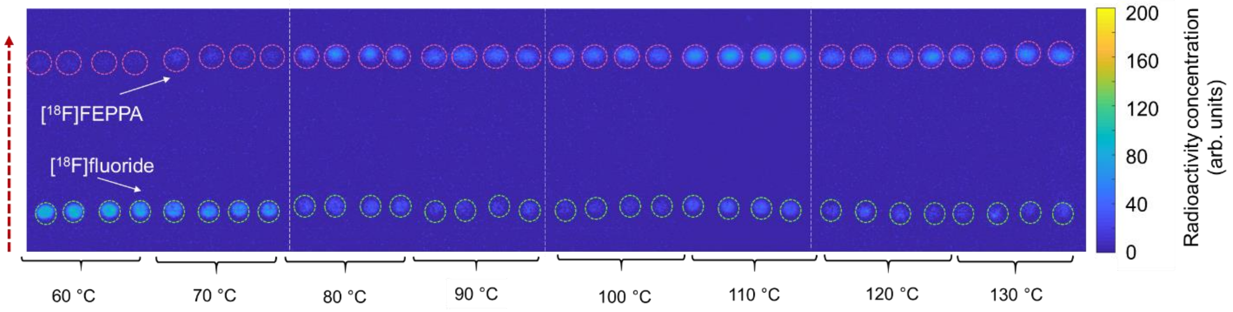

Figure S43. Cerenkov images of TLC plates (each containing 8 samples) after developing in the mobile phase. In this case, each TLC plate contains samples from two different temperatures (n=4 replicates each). White dotted lines show the edges of each separate multi-sample TLC plate. Dashed circles indicate the ROIs used for analysis. The dashed red arrow indicates the direction of solvent movement during developing.

Table S21. Summary of data acquired when exploring the effect of temperature on the radiosyntheses of [^{18}F]FEPPA.

Temperature (°C)	Collection efficiency (%) n=4	Fluorination efficiency (%) n=4	Crude RCY (%) n=4	Activity left on chip (%) n=4
60	94.1 ± 1.6	11.9 ± 1.8	11.2 ± 1.5	2.5 ± 0.4
70	91.6 ± 1.7	22.8 ± 2.0	20.9 ± 1.7	2.1 ± 0.3
80	91.6 ± 0.9	65.1 ± 1.1	59.6 ± 1.2	1.8 ± 0.2
90	92.7 ± 1.5	77.5 ± 1.6	71.8 ± 1.8	1.7 ± 0.2
100	93.8 ± 1.5	81.3 ± 1.5	76.3 ± 2.6	1.0 ± 0.2
110	92.9 ± 1.9	83.0 ± 1.4	77.1 ± 2.0	1.0 ± 0.3
120	91.4 ± 1.0	75.5 ± 1.8	69.0 ± 1.5	1.2 ± 0.2
130	90.6 ± 1.1	75.2 ± 2.7	68.1 ± 3.0	1.6 ± 0.1

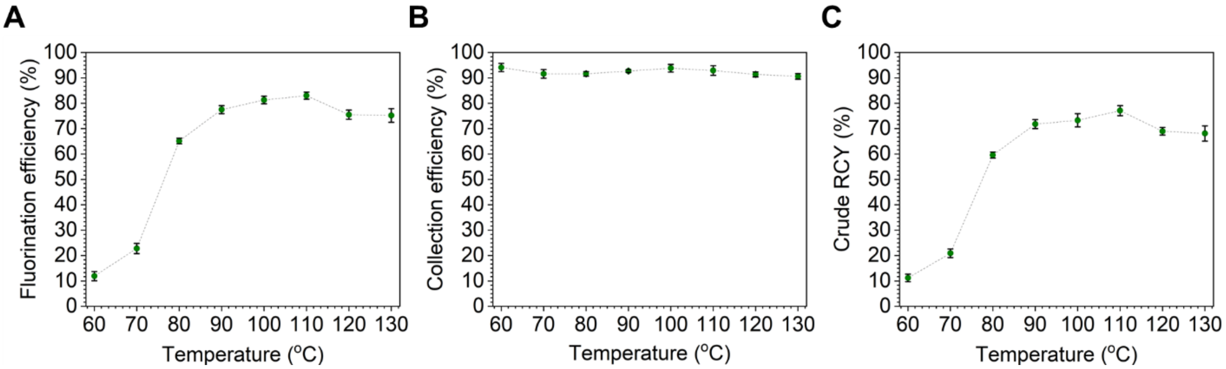

Figure 44. Effect of temperature on the performance of [^{18}F]FEPPA synthesis. Reaction volume: 8 µL. Base amount: 240 nmol. Precursor amount: 240 nmol. Reaction time: 2 min. Reaction solvent: thexyl alcohol and MeCN (1:1 v/v) (A) Effect on fluorination efficiency. (B) Effect on collection efficiency. (C) Effect on crude RCY.

8.2 Comparison to literature methods

Table S22. Comparison of optimized droplet conditions to literature reports for conventional and flow-chemistry synthesis of [^{18}F]FEPPA.

	**This work (2021)**	**Chang et al.[13] (2021)**	**Dahl et al.[14] (2019)**	**Vignal et al.[15] (2018)**	**Berroteran-Infante et al.[16] (2018)**
**Synthesizer type**	Microscale (droplet format)	Macroscale	Microscale (flow format)	Macroscale	Macroscale
**Base type**	$TBAHCO_3$	K_{222} / K_2CO_3	K_{222} / $KHCO_3$	K_{222} / K_2CO_3	K_{222} / K_2CO_3
**Base amount (nmol)**	240	28000/ 20000	2000/2000	40000/22000	58000/32000
**Precursor amount (nmol)**	240	9000	9000	9000	12000
**Reaction solvent**	thexyl alcohol: MeCN (1:1 v/v)	MeCN	MeCN	MeCN	MeCN
**Reaction volume (mL)**	0.008	0.6	0.2	1	0.5
**Temperature (°C)**	90	90	80	90	90
**Reaction time (min)**	2	10	10	10	10
**Starting activity (MBq [mCi])**	13.8 [0.375]	N.R.	3700 [100]	N.R.	N.R.
**Fluorination efficiency (%)**	88.5 ± 0.9 (n=4)	N.R.	N.R.	N.R.	N.R.
**Crude RCY (decay-corrected; %)**	80.7 ± 1.2 (n=4)	N.R.	N.R.	N.R.	N.R.
**Activity yield (%)**	53 (n=1)	30 ± 2 (n=8)	29*	34 ± 2 (n=17)	38 ± 3 (n=15)
**RCY (decay-corrected; %)**	67 (n=1)	50 ± 2 (n=8)	51 ± 6 (n=3)*	48 ± 2 (n=17)	46 ± 3 (n=15)
**Synthesis time (min)**	30	80	55*	55	30

* Isolated yield without formulation

N.R. = not reported.

9 Representative chromatograms

9.1 [^{18}F]Flumazenil

Crude [^{18}F]Flumazenil was injected in HPLC to isolate the product (**Figure S45A**). A subsequent injection was performed to confirm purity (**Figure S45B**), and a co-injection with Flumazenil reference standard was performed to confirm product identity (**Figure S45C**). The crude product showed minimal UV impurities.

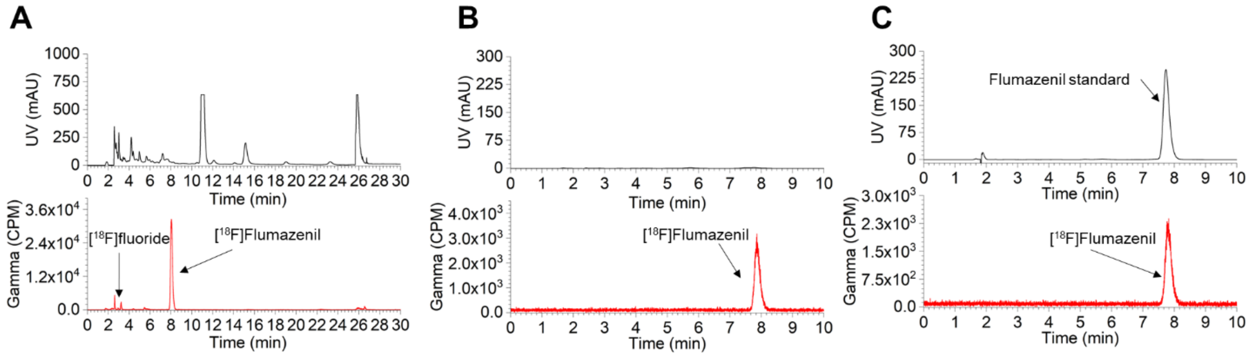

Figure S45. HPLC chromatograms of [^{18}F]Flumazenil. (A) Crude product. (B) Isolated product. (C) Co-injection with reference standard.

9.2 [^{18}F]PBR06

Crude [^{18}F]PBR06 was injected in HPLC to isolate the product (**Figure S46A**). A subsequent injection was performed to confirm purity (**Figure S46B**), and a co-injection with PBR06 reference standard was performed to confirm product identity (**Figure S46C**). The crude product showed minimal UV impurities.

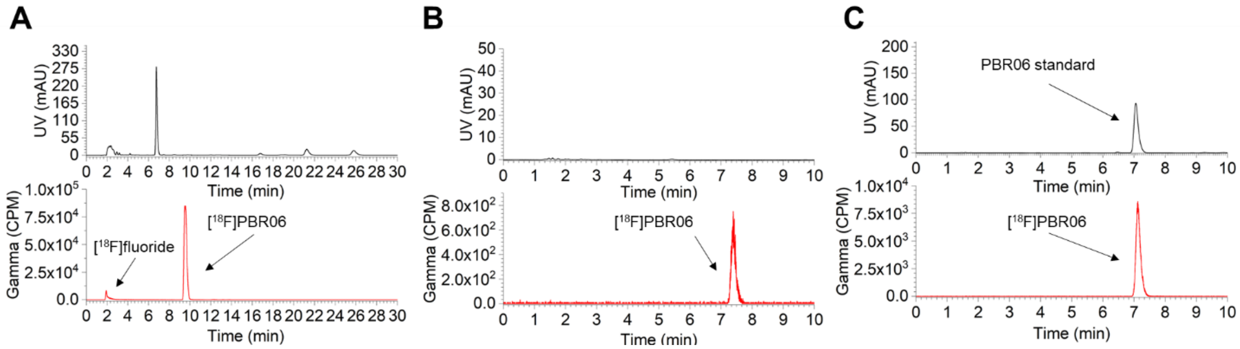

Figure S46. HPLC chromatograms of [^{18}F]PBR06. (A) Crude product. (B) Isolated product. (C) Co-injection with reference standard.

9.3 [18F]Fallypride

Crude [18F]Fallypride was injected in HPLC to isolate the product (**Figure S47A**). A subsequent injection was performed to confirm purity (**Figure S47B**), and a co-injection with Fallypride reference standard was performed to confirm product identity (**Figure S47C**). The crude product showed minimal UV impurities.

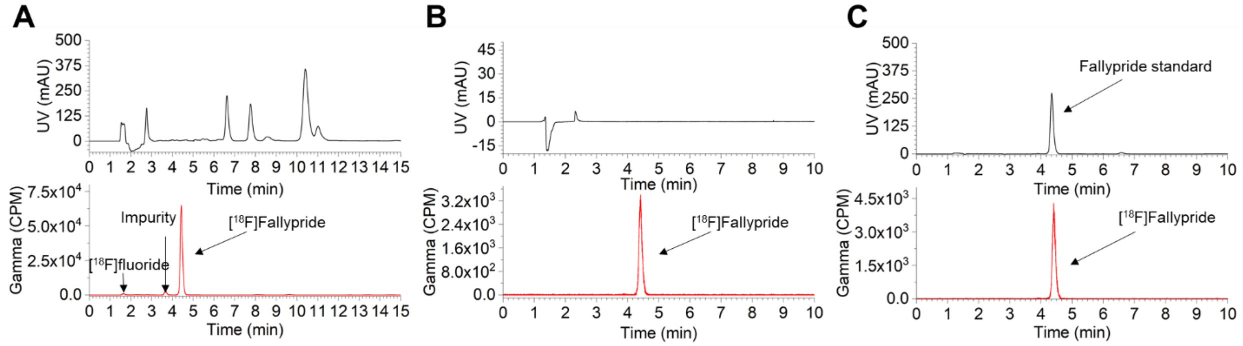

Figure S47. HPLC chromatograms of [18F]Fallypride. (A) Crude product. (B) Isolated product. (C) Co-injection with reference standard.

9.4 [18F]FEPPA

Crude [18F]FEPPA was injected in HPLC to isolate the product (**Figure S48A**). A subsequent injection was performed to confirm purity (**Figure S48B**), and a co-injection with FEPPA reference standard was performed to confirm product identity (**Figure S48C**). The crude product showed minimal UV impurities.

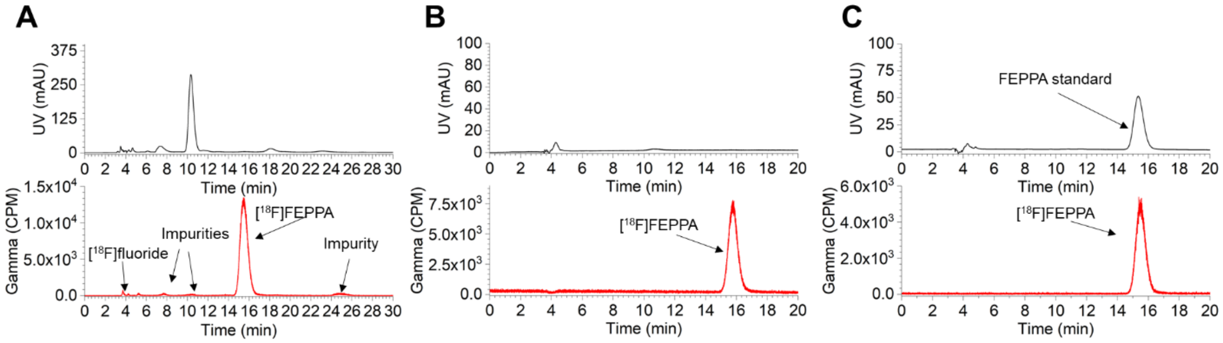

Figure S48. HPLC chromatograms of [18F]FEPPA. (A) Crude product. (B) Isolated formulated product. (C) Co-injection with reference standard.

10 Clinical-scale radiosynthesis

Summaries of high activity droplet syntheses of [^{18}F]PBR06 carried out with different starting activity are shown in **Table S23** and **Figure S49.**

Table S23. Synthesis performance of [^{18}F]PBR06 at increased activity levels.

Starting activity (MBq [mCi])	Number of replicates	Collection efficiency (%)	Fluorination efficiency (%)	Crude RCY (%)
14.1 [0.38]	n=4	95.8 ± 3.5	96.5 ± 0.9	92.4 ± 3.0
651 [17.6]	n=1	94.0	87.1	81.0
1120 [30.3]	n=1	90.3	80.9	73.0
2200 [59.7]	n=1	90.9	68.6	62.4
2390 [64.7]	n=1	89.5	88.7	71.5
2960 [80.8]	n=1	91.0	79.3	72.1
3170 [85.6]	n=1	90.3	65.6	59.2

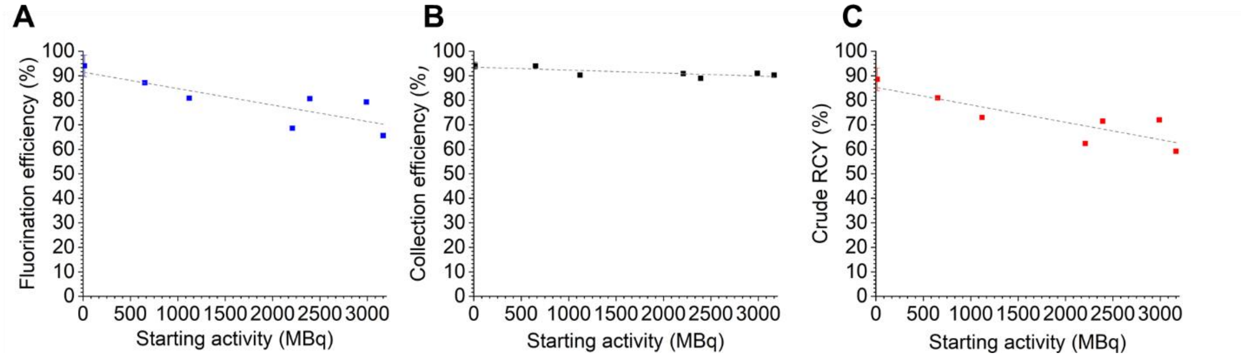

Figure S49. Comparison of [^{18}F]PBR06 synthesis performance for different starting activities. (A) Fluorination efficiency. (B) Collection efficiency. (C) Crude RCY.

11 References

1. Ryzhikov, N. N. *et al.* Preparation of highly specific radioactivity [18F]flumazenil and its evaluation in cynomolgus monkey by positron emission tomography. *Nuclear Medicine and Biology* **32**, 109–116 (2005).
2. Vaulina, D., Nasirzadeh, M. & Gomzina, N. Automated radiosynthesis and purification of [18F]flumazenil with solid phase extraction. *Applied Radiation and Isotopes* **135**, 110–114 (2018).
3. Nasirzadeh, M., Vaulina, D. D., Kuznetsova, O. F. & Gomzina, N. A. A novel approach to the synthesis of [18F]flumazenil, a radioligand for PET imaging of central benzodiazepine receptors. *Russ Chem Bull* **65**, 794–800 (2016).
4. Mandap, K. S. *et al.* Development of microwave-based automated nucleophilic [18F]fluorination system and its application to the production of [18F]flumazenil. *Nuclear Medicine and Biology* **36**, 403–409 (2009).
5. Rios, A., Wang, J., Chao, P. H. & Dam, R. M. van. A novel multi-reaction microdroplet platform for rapid radiochemistry optimization. *RSC Adv.* **9**, 20370–20374 (2019).
6. Wang, J., Holloway, T., Lisova, K. & Dam, R. M. van. Green and efficient synthesis of the radiopharmaceutical [18F]FDOPA using a microdroplet reactor. *React. Chem. Eng.* (2019) doi:10.1039/C9RE00354A.
7. Lisova, K. *et al.* Rapid, efficient, and economical synthesis of PET tracers in a droplet microreactor: application to O-(2-[18F]fluoroethyl)-L-tyrosine ([18F]FET). *EJNMMI radiopharm. chem.* **5**, 1 (2019).
8. Wong, R. *et al.* Reactivity of electrochemically concentrated anhydrous [18F]fluoride for microfluidic radiosynthesis of 18F-labeled compounds. *Applied Radiation and Isotopes* **70**, 193–199 (2012).
9. Massaweh, G. *et al.* Improved work-up procedure for the production of [18F]flumazenil and first results of its use with a high-resolution research tomograph in human stroke. *Nuclear Medicine and Biology* **36**, 721–727 (2009).

10. Lisova, K., Wang, J., Chao, P. H. & van Dam, R. M. A simple and efficient automated microvolume radiosynthesis of [18F]Florbetaben. *EJNMMI Radiopharmacy and Chemistry* **5**, 30 (2020).

11. Wang, M., Gao, M., Miller, K. D. & Zheng, Q.-H. Synthesis of [11C]PBR06 and [18F]PBR06 as agents for positron emission tomographic (PET) imaging of the translocator protein (TSPO). *Steroids* **76**, 1331–1340 (2011).

12. Zhang, H. *et al.* 18F-PBR06 PET/CT imaging for evaluating atherosclerotic plaques linked to macrophage infiltration. *Nucl Med Commun* **40**, 370–376 (2019).

13. Chang, C.-W. *et al.* GMP-compliant fully automated radiosynthesis of [18F]FEPPA for PET/MRI imaging of regional brain TSPO expression. *EJNMMI Res* **11**, 26 (2021).

14. Dahl, K., Garcia, A., Stephenson, N. A. & Vasdev, N. “In-loop” 18F-fluorination: A proof-of-concept study. *Journal of Labelled Compounds and Radiopharmaceuticals* **62**, 292–297 (2019).

15. Vignal, N. *et al.* [18F]FEPPA a TSPO Radioligand: Optimized Radiosynthesis and Evaluation as a PET Radiotracer for Brain Inflammation in a Peripheral LPS-Injected Mouse Model. *Molecules* **23**, 1375 (2018).

16. Berroterán-Infante, N. *et al.* [18F]FEPPA: Improved Automated Radiosynthesis, Binding Affinity, and Preliminary in Vitro Evaluation in Colorectal Cancer. *ACS Med. Chem. Lett.* **9**, 177–181 (2018).
